# Supplementary material for: Reviving collapsed plant–pollinator networks from a single species
Source: PLoS Biol. 2024 Oct 4;22(10):e3002826. doi: 10.1371/journal.pbio.3002826 (PMC11482677; doi:10.1371/journal.pbio.3002826)
Supplement: S1 Text — (PDF) [file pbio.3002826.s001.pdf]

S1 text: Reviving collapsed plant-pollinator networks from a single species

Gaurav Baruah[1*], Meike Wittmann[1]
[1]Faculty of Biology, Theoretical Biology, University of Bielefeld, Bielefeld, Germany

---

---

1. Mutualistic eco-evolutionary model

1.1. Quantitative Genetics and Lotka-Volterra dynamics

We model the dynamics of pollinators and plants in an ecologically relevant quantitative trait z. Each individual belonging to a guild of pollinators or plants can be described with its trait z, but each of the species belonging to the guild of pollinators or plants are comprised of individuals with different trait values. Now the number of individuals within species i at time t for pollinators (animals) will be $N_i^{(A)}(t)$ and for the plants it will be $N_i^{(P)}(t)$, and the distribution of their traits within each species i can be given by a function $p_i^{(A,P)}(z,t)$ and by definition this function satisfies

$$\int p_i^{(A,P)}(z,t)dz = 1$$

at every time t; the limits of integration encompass the whole trait axis, which for simplicity we take to go between minus and plus infinity unless otherwise noted. $N_i^{(P)}(t)p_i^{(P)}(z,t)dz$ is then the population density of species i's individuals with phenotype value between z and $z+dz$ for the plants. Analogously, for the pollinators we can write $N_i^{(A)}(t)p_i^{(A)}(z',t)dz'$ which is the population density with phenotype value between z' and $z'+dz$. Here z' distinguishes from an individual phenotype of a plant species, z.

We work in the quantitative genetic limit, i.e., the trait in question is determined by many independent loci. Due to this, the following important results hold (Bulmer 1980, Falconer 1981): 1) the trait is normally distributed and 2) the variance of the trait does not change in response to any kind of selection pressure. Thus, the trait distribution than can be written as:

$$p_i^{(A,P)}(z,t) = \frac{1}{\sqrt{2\pi(\sigma_i^{(A,P)})^2}} \exp\frac{-(z-u_i^{(A,P)}(t))^2}{2(\sigma_i^{(A,P)})^2},$$

where $u_i^{(A,P)}$ is the mean trait value for the species i (could be plants or pollinators) and $(\sigma_i^{(A,P)})^2$ is the trait variance. Here, we assume there are no environmental effects, i.e., no plasticity in the trait. Thus, the trait variance $(\sigma_i^{(A,P)})^2$ can also be called as the genetic variance. In this scenario, only the mean of the trait responds to selection pressure and the trait variance remains constant and fixed in time. Moreover, the distribution of the mean trait remains normal.

The governing dynamical equations of population dynamics can be written with a slightly modified Lotka-Volterra equations. From [1, 2], the per-capita growth rate can be written as (for both plants and pollinators) shown here is the per-capita growth rate of pollinator species i:

$$r^{(A)}(z,t) = b - \sum_j \alpha_{ij}^{(A)} N_j^{(A)} + \int \sum_k A_{ki} \frac{\gamma(z,z')N_k^{(P)}}{1 + H\gamma(z,z')N_k^{(P)}} p_k^{(P)}(z',t)dz' \quad (1)$$

where b is the growth rate independent of competition or mutualistic benefits; $a_{ij}^{(A,P)}$ is the pairwise competition term among species belonging to each own guild; H is the handling time. For simplicity, we are going to assume that competition is not influenced by our trait of interest z; $\gamma(z, z')$ is the function that captures the mutualistic interactions among individuals belonging to two different guilds: plants and pollinators. Here, z is the trait of an individual of a species belonging to a guild say the pollinators, and z' is the trait of an individual belonging to the plants. We can take this function to be a Gaussian:

$$\gamma(z, z') = \frac{\gamma_0}{d_i^{(A,P)}} \exp \frac{-(z - z')^2}{w^2}$$

where, γ_0 is the average strength of mutualistic interactions and w is the width that controls how strongly two individuals interact. The more similar traits of two individuals belonging to two different species, namely plants and pollinators, the stronger is the mutualistic benefit.

Equation 1 in this S1 text represents the per-capita growth rate of an individual with phenotype z interacting facilitatively with another individual with phenotype z' belonging to a species of another guild and $p_k^{(A,P)}(z', t)$ is the distribution of the trait z'. The integration goes over the entire trait space and summed for all the species belonging to a guild. This formulation of the model is special in the sense that growth and mutualistic interactions only depend on the phenotype z but not on species identity. Also note, that mutualistic interaction follows here as a type 2 functional curve. If $H = 0$, mutualistic benefits follows a linear type 1 functional curve.

Now the population dynamics of species i (could be represented similarly for both plants or pollinators, but shown here for pollinators) over all trait space z can be written as:

$$\frac{dN_i^{(A)}}{dt} = N_i^{(A)}(t) \int r(z) p_i^{(A)}(z, t) dz \tag{2}$$

Substituting equation 1 into 2 we get

$$\frac{dN_i^{(A)}}{dt} = N_i^{(A)}(t) \int \left(b - \sum_j \alpha_{ij}^{(A)} N_j^{(A)} + \int \sum_k A_{ki} \frac{\gamma(z, z') N_k^{(P)}}{1 + H\gamma(z, z') N_k^{(P)}} p_k^{(P)}(z', t) dz' \right) p_i^{(A)}(z, t) dz \tag{3}$$

We can further solve equation 3 as:

$$\frac{dN_i^{(A)}}{dt} = N_i^{(A)}(t) \left(b - \sum_j \alpha_{ij}^{(A)} N_j^{(A)} + \int \int \sum_k A_{ki} \frac{\gamma(z, z') N_k^{(P)}}{1 + H\gamma(z, z') N_k^{(P)}} p_k^{(P)}(z', t) p_i^{(A)}(z, t) dz dz' \right) \tag{4}$$

In our model, we fix intrinsic growth b to be constant at zero, and independent of trait z. $b = 0$ for all species means species are considered obligate mutualists.

Since the competition among species within a guild is independent of the phenotype we modelled and from $\int p_i^{(A)}(z, t) dz = 1$, we get,

$$\int \sum_j \alpha_{ij}^{(A)} N_j p_i(z, t) dz = \sum_j \alpha_{ij}^{(A)} N_j$$

Assuming the quantitative genetic limit, the dynamics of the mean phenotype $u_i^{(A)}(t)$ of interest

can then be written as, shown here for the pollinators from [1, 2],

$$\frac{du_i^{(A)}}{dt} = h^2 \int (z - u_i^{(A)}) \left(b - \sum_j \alpha_{ij}^{(A)} N_j + \int \sum_k A_{ki} \frac{\gamma(z, z') N_k^{(P)}}{1 + H\gamma(z, z') N_k^{(P)}} p_k^{(P)}(z', t) dz' \right) p_i^{(A)}(z, t) dz \quad (5)$$

where h^2 is the broad sense heritability of the mean trait.

2. Modularity, and betweenness centrality of networks

Modularity captures how structured or how clustered a mutualistic network is. Plant-pollinator networks with high modularity will have modules or groups of species that have more interactions between them but have sparse interactions with species outside of these modules or groups of species. Biological networks such as mutualistic networks show a high degree of modularity. Previous studies have shown that high degree of modularity in spatial arrangement of patches, could buffer a population from extinction by alleviating the propagation of perturbation in the population [3]. On the other hand, we expect that plant-pollinator networks with high modularity recover less or recover at higher γ_0 than less modular networks. Indeed that is what we observe in this S10 Fig, particularly for low trait variation, and slightly for high trait variation. Networks which had high modularity i.e., modularity > 0.7 recovered at higher γ_0 (in S10 Fig and in S12 Fig).

There are many measures of centrality of a network, one such is betweenness centrality, the other is degree centrality. Degree centrality is just the degree of a species in a network, which we already consider in our study. We thus calculated betweenness centrality for each species in a plant-pollinator network. betweenness centrality is a measure of centrality in a network based on a measure of shortest paths that pass through each species in a network. For instance, betweenness centrality of a species in a network is the number of short paths/interactions/edges that connects to that species. Thus, a species with high betweenness centrality would mean that the species would be a central species to that network in the sense that there are a lot of short paths that goes through that species. We measured median betweenness centrality of a network after normalising the betweenness centrality measure. Betweenness centrality of a species i in a network is given as:

$$B_i = \sum_{a,b} \frac{s_{aij}}{s_{aj}}$$

where, s_{aj} is the number of shortest paths from species a and species j and s_{aij} is the number of shortest paths from a to j that goes through species i. We then normalise B_i to have maximum value of 1 and minimum value of 0, by this transformation: $\frac{B_i - min(B_i)}{max(B_i) - min(B_i)}$ such that 1 is the highest betweenness centrality of a species, and 0 is the lowest betweenness centrality of a species. With this, we estimated median network centrality by taking the median value of B_i for a network. Overall, a network with high median network centrality would mean that in that network there are more number of species that are central to that network in terms of interactions. Thus, a network with high median betweenness centrality might recover early at lower γ_0 thresholds as we observe in this S1 text and this S13 Fig and in S10B Fig.

3. Relaxing the assumption of Gaussian interaction kernel

Our model includes various parameters and functions that can be altered. One key assumption is that mutualistic interactions follow a Gaussian interaction kernel, meaning similar phenotypes have stronger symmetric mutualistic benefits than dissimilar ones. This assumption is based on empirical studies showing that plant-pollinator interactions are strongest when phenotypes, such as an insect's proboscis and a flower's corolla length, match. Additionally, Gaussian kernels are commonly used in theoretical studies due to their ease in analytical integration.

To test the robustness of our model, we also relaxed this Gaussian assumption. Although biologically motivated and supported by some studies, symmetric interactions may not fully capture the diversity of natural phenomena. Thus, we implemented a variant by replacing the Gaussian kernel with an asymmetric one, described by a scaled and shifted Gamma distribution. This particular distribution does not lead to the highest mutualistic benefits when phenotypes are very similar, but if the difference in their phenotypic values takes a certain value as shown in S16 Fig.

We evaluated the impact of such an asymmetric interaction kernel by numerical integration and show the results in this S17-19 Fig. As seen in S17 Fig and S18 Fig, asymmetric interaction kernel does lead to slight differences in the results in contrast to when species interaction in a symmetric Gaussian kernel. Particularly, in S17 Fig we observed that when species interacted in an asymmetric manner, network recovery was still achieved but only at higher γ_0 thresholds in comparison to when species interacted in a symmetric Gaussian manner. Thus, as shown in S18 Fig, network recovery was further slightly constrained due to the manner in which species interacted.

4. Contribution of indirect effects to network recovery

Our modelling framework used a modified version of the generalised Lotka-Volterra model with type-2 functional curve for plant-pollinator interactions. In a community of multiple species, there are several ways species can interact. One of them would be direct interactions such as a pollinator competiting with another pollinator for pollen. Another direct interaction would be a pollinator collecting nectar from a flower. However, there could be also indirect interactions in such a community where a species i could have an impact on species k through another species j in the community. This is a second-order indirect interaction between species i and species k. If a_{ij} is a direct interaction between species i and species j, then indirect interaction of order 3 between species j and species i would then be $a_{il} \times a_{lk} \times a_{kj}$. Thus, as the number of species in a community grows, and a_{ij} values are less than 1, then the magnitude of indirect interactions will decay exponentially as number of species grows. This is because indirect interactions are defined according to [4] as the product of the direct interaction of species j on species k with the direct interaction of species k on species i. Nevertheless, one can quantify under certain approximations the net indirect interactions in a community by first calculating the net total interactions, which is a culmination of direct and indirect interactions. Following [4, 5, 6], a simple generalised Lotka-Volterra equation can be written as:

$$\frac{dN_i}{dt} = N_i(b_i + \sum_{j=1}^{S} B_{ij}N_j) \tag{6}$$

Here, $N_i \epsilon (N_i^A, N_i^P)$

$$B = \begin{pmatrix} \alpha_{S_A \times S_A} & \gamma_{S_A \times S_P} \\ \gamma_{S_P \times S_A} & \alpha_{S_P \times S_P} \end{pmatrix},$$

where $\alpha_{S_A \times S_A}$ is the matrix of competition coefficients of dimension of $S_A \times S_A$, where S_A is the number of animal species, and S_P is the number of plant species. $\gamma_{S_A \times S_P}$ is the matrix of mutualistic interaction coefficients of dimension $S_A \times S_P$. While quantifying both net and indirect interactions in our model, the assumption that some sort of ecological equilibrium is reached by the system is needed. This would make formalism easier and concurrently account for long-term effects. So our following formulation will be based on the assumption that our plant-pollinator system is at some kind of equilibrium. To be realistic, this is not possible, as we use a time-dependent forcing of a system that changes over time and shifts from one state to another. Hence, we have to make some assumptions following our estimation of indirect effects on the recovery of the plant-pollinator systems. Also to be noted here is that, although [5] quantified indirect effects on evolutionary dynamics, here, the goal is not to quantify indirect effects on evolutionary dynamics, but rather on recovery biomass of the plant-pollinator systems. The first assumption is that we use a type-1 functional curve for estimation of the net indirect effects in our system. This means we are assuming that handling time is zero, such that the type-2 functional form collapses to a type-1 functional form. Even if handling time is not necessarily zero, we still could be fine in assuming a type-1 functional form provided we calculate net indirect effects at the start of our perturbation regime and when all densities are very low. Our starting point of the modelling framework considers the plant-pollinator systems to be at a stable although undesirable state i.e., all species i $N_i < 0.005$ density. At such low densities, interactions between plants and pollinators can be considered to be at the type-1 functional form, so our assumption of using a linear Lotka-Volterra approach makes sense. We then relate the calculation of indirect effects at the starting point of the dynamics to the final recovery biomass of the plant-pollinator systems. This is not ideal, but the best possible outcome given our modelling framework, where a time-dependent species-specific forcing was used.

From equation 6, **B** has both within-guild competition and between-guild plant-pollinator interactions. The competition coefficients will be in the block diagonal matrix, whereas the plant-pollinator interaction coefficients will be in the off-diagonal block matrix. In our model, b_i was considered zero. With a linear functional curve, the double integral from equation 3 can be easily solve to get an exact term for mutualistic interaction coefficients. Thus, from equation 3, because $N_i < 0.005$ are very low, and with low handling time, we can safely assume that $1 + H\gamma(z, z')N_k^{(P)} \approx 1$. Hence, the exact coefficients for mutualistic coefficients of matrix **B** as:

$$\gamma_{ik} = \int \int \sum_k A_i \gamma(z, z') N_k^{(P)} p_k^{(P)}(z', t) p_i^{(A)}(z, t) dz dz' = \tag{7}$$

$$A_{ki}\gamma_0 \frac{\omega}{\sqrt{2\sigma_i^2 + 2\sigma_k^2 + \omega^2}} \exp \frac{-(u_i - u_k)^2}{2\sigma_i^2 + 2\sigma_k^2 + \omega^2}. \tag{8}$$

Here, σ_i^2 is the variance in trait of species i, and u_i and u_k are the mean trait values of species i and k at initial time point before the start of the perturbation.

Having now the information of the coefficients of $\mathbf{B}$, we can finally derive the net indirect effects. From [6], the direct interaction between species quantifies the change in growth rate of a species to a change in the density of the other. If $r_i = b_i + \sum_{j=1}^{S} B_{ij} N_j$ quantifies the growth rate of species i, then direct interactions can be quantified in matrix formulation as:

$$\frac{\partial \mathbf{r}}{\partial \mathbf{N}} \tag{9}$$

In addition, following [6], the net interaction then is the inverse of the direct interactions and can be written as:

$$\frac{\partial \mathbf{N}^*}{\partial \mathbf{r}} = \Big(- \frac{\partial \mathbf{r}}{\partial \mathbf{N}} \Big)^{-1} \tag{10}$$

To compare direct, net, and indirect interactions with each other, it is necessary to have the dimensions of these three to be the same. Direct and net interactions have reciprocal units following the equation 9 and equation 10. For this, defining the direct interactions in relation to intraspecific interaction, we can define interactions i.e., B_{ij} as

$$B_{ij} = \frac{\partial r_i}{\partial N_j} / \Big(- \frac{\partial r_i}{\partial N_i} \Big)$$

With this, the non-dimensional direct interactions can be then written as [6]:

$$\frac{\partial \mathbf{r}}{\partial \mathbf{N}} = \Big(- \mathbf{I} + \mathbf{B} \Big) \tag{11}$$

And from equation 10, we can get

$$\frac{\partial \mathbf{N}^*}{\partial \mathbf{r}} = \Big(- \frac{\partial \mathbf{r}}{\partial \mathbf{N}} \Big)^{-1} = \Big(\mathbf{I} - \mathbf{B} \Big)^{-1}, \tag{12}$$

where $(\boldsymbol{I} - \mathbf{B})^{-1}$ is the matrix of net interactions that contains all interactions ranging from direct to indirect interactions. Now $(\boldsymbol{I} - \mathbf{B})^{-1}$ is a Neumann's series [5, 6, 7]

$$(\boldsymbol{I} - \mathbf{B})^{-1} = \boldsymbol{I} + \mathbf{B} + \mathbf{B}^2 + \mathbf{B}^3 + ..., \tag{13}$$

where $\mathbf{B}$ is the matrix of direct indirections and $\mathbf{B}^2$ is a matrix of second-order indirect interactions, $\mathbf{B}^3$ is the matrix of third-order indirect interactions. From this, equation 13, can be further separated to give us the net indirect interactions, which is:

$$(\boldsymbol{I} - \mathbf{B})^{-1} - (\boldsymbol{I} + \mathbf{B}) = \mathbf{B}^2 + \mathbf{B}^3 + ... \tag{14}$$

$$\mathbf{B}^2 (\boldsymbol{I} - \mathbf{B})^{-1} = \mathbf{B}^2 + \mathbf{B}^3 + ... \tag{15}$$

Thus, the L.H.S of equation 15 captures all the indirect interactions present in a system that follows equation 6 and is at a equilibria.

With this, we further calculated mean indirect effects faced by a species j i.e., B_j, per network as:

$$\beta = \frac{\sum_{i,j}^{S} B_{ij}}{S} = \frac{\sum_{i,j}^{S} B_{ij}^2 (1 - B_{ij})^{-1}}{S} \tag{16}$$

Finally, we further quantified mean indirect effects of order 3 and order 4 i.e., $\mathbf{B}^3$ and $\mathbf{B}^4$ as β^3 and β^4 respectively. Finally, quantifying indirect effects will only make sense if the spectral radius, i.e., the absolute maximum of the eigenvalue of the matrix $\mathbf{B}$ is less than 1 [6]. If spectral radius becomes greater than 1, then net interactions is not equal to the sum of direct and indirect interactions or equation 13 does not converge. In that sense, estimating β will not make sense.

In S14 Fig and S15 Fig, we observe that indirect interaction effects are mostly negative, indicating that at low γ_0 values, indirect interactions promotes hysteresis. Indirect effects being negative is indicative of competitive effects that we modelled within each guild of species. At higher values of $\gamma_0 > 1$, we observe that spectral radius, i.e., the absolute maximum of the eigenvalue of the matrix $\mathbf{B}$ becomes greater than 1, indicating that our linearity assumption with type-1 functional curve might have failed, as at higher γ_0 values species densities slightly increase, which might have led to spectral radius being greater than 1. Note that [5] did not have competitive interactions modeled in contrast to our study.

5. Creating Nestedness matrices

To disentangle the true impact of nestedness on recovery of networks from perturbing a single species, we would need to ensure that only nestedness in the network varies, with the size of the network and connectance remaining constant. For that, we start with a network size of 30 species with 15 plants and 15 animals and create nestedness matrices by arranging species adjacency matrix. While maintaining a connectance of 0.42, we shuffle the elements of the adjacency matrices to create matrices of varying nestedness. Doing that we created sixty different nestedness matrices that varied from as high as 1 to as low as 0.38. See in this S34 Fig for impact of only varying nestedness on recovery richness from perturbing the most generalist species. Overall, we observed that nestedness does have a positive impact, but its impact was weaker. This demonstrated that it was the combination of nestedness, connectance and network size that impacted the revival of networks from perturbing the generalist species.

Table A: Table for results of linear mixed model of Seychelles data with mean pollinator visits as the response variable

Predictors	Slope	Standard error	p-value
Intercept	5.63	1.676	0.001
Nestedness	12.32	4.493	0.008*
Treatment (restored)	1.000	1.377	0.498
**Random effects**	**Variance**		
Site	3.10		
month	2.44		

Table B: Table for results of linear mixed model of Seychelles data with mean visitation rate per network as the response variable

Predictors	Slope	Standard error	p-value
Intercept	-0.1	1.196	0.93
Nestedness	8.17	3.84	0.039*
Treatment (restored)	-0.14	0.723	0.843
**Random effects**	**Variance**		
Site	0.32		
month	0.24		

References

[1] Baruah G. The impact of individual variation on abrupt collapses in mutualistic networks. Ecology Letters. 2022;25(1):26–37. doi:10.1111/ele.13895.

[2] Barabas G, D'Andrea R. The effect of intraspecific variation and heritability on community pattern and robustness. Ecology Letters. 2016;19(8):977–986.

[3] Gilarranz LJ, Rayfield B, Liñán-Cembrano G, Bascompte J, Gonzalez A. Effects of network modularity on the spread of perturbation impact in experimental metapopulations. Science. 2017;357(6347):199–201. doi:10.1126/science.aal4122.

[4] Neutel AM, Heesterbeek JAP, de Ruiter PC. Stability in Real Food Webs: Weak Links in Long Loops. Science. 2002;296(5570):1120–1123. doi:10.1126/science.1068326.

[5] Guimarães PR, Pires MM, Jordano P, Bascompte J, Thompson JN. Indirect effects drive coevolution in mutualistic networks. Nature. 2017;550(7677):511–514. doi:10.1038/nature24273.

[6] Zelnik YR, Galiana N, Barbier M, Loreau M, Galbraith E, Arnoldi JF. How collectively integrated are ecological communities? Ecology Letters. 2024;27(1):e14358. doi:10.1111/ele.14358.

[7] Sasane A. A Friendly Approach to Functional Analysis | Mathematical Association of America. vol. 1; 2017. Available from: https://maa.org/press/maa-reviews/a-friendly-approach-to-functional-analysis.
