# Supplementary figures and images for: Reviving collapsed plant–pollinator networks from a single species
Source: PLoS Biol. 2024 Oct 4;22(10):e3002826. doi: 10.1371/journal.pbio.3002826 (PMC11482677; doi:10.1371/journal.pbio.3002826)

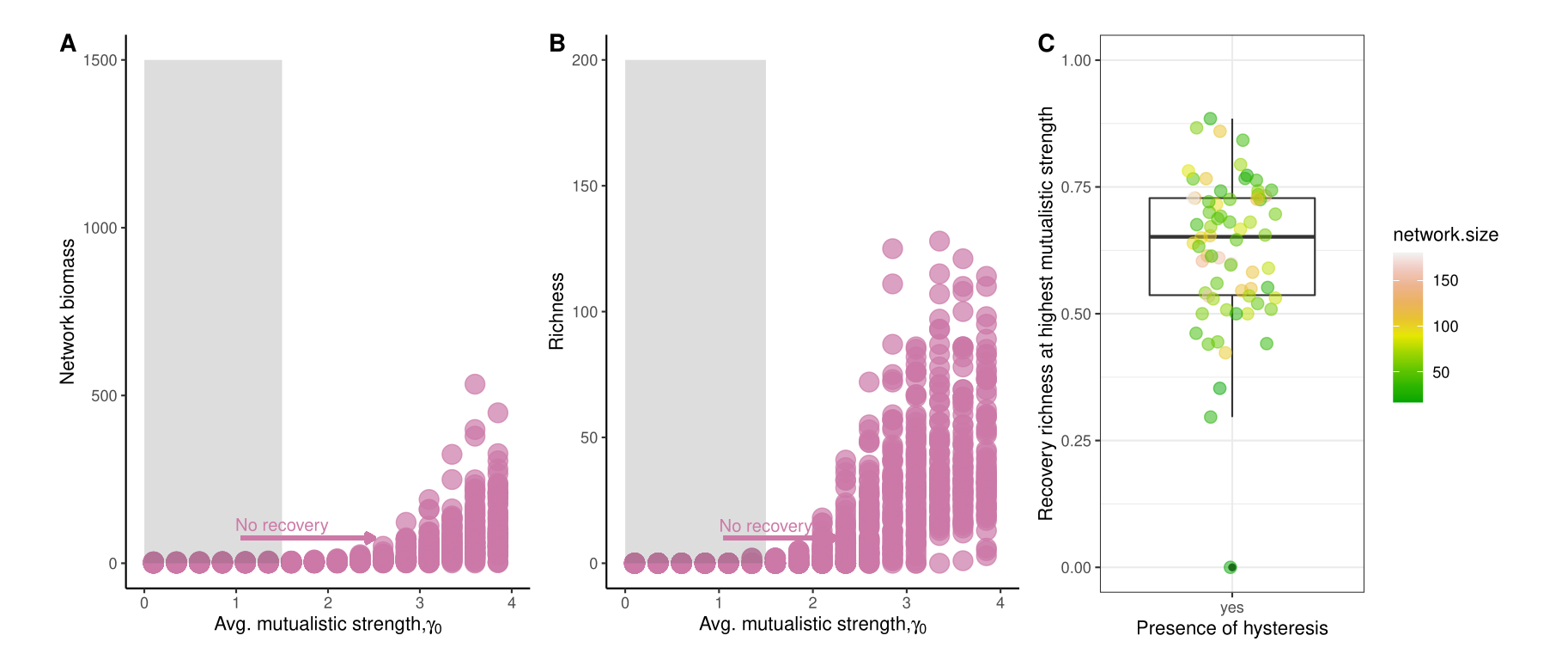

Supplement: S1 Fig — (A, B) None of the 115 networks recovered to its original high functional stable state despite reverting to the original environmental conditions. (C) Recovery richness of networks at average mutualistic γ0 = 4.5. Here, recovery richness of 1 means all species have recovered, i.e., Ni(A,P)>0.5. Underlying data and R scripts for reproducing this figure can be found in https://doi.org/10.5281/zenodo.13598906. (TIF) [file pbio.3002826.s002.tif]

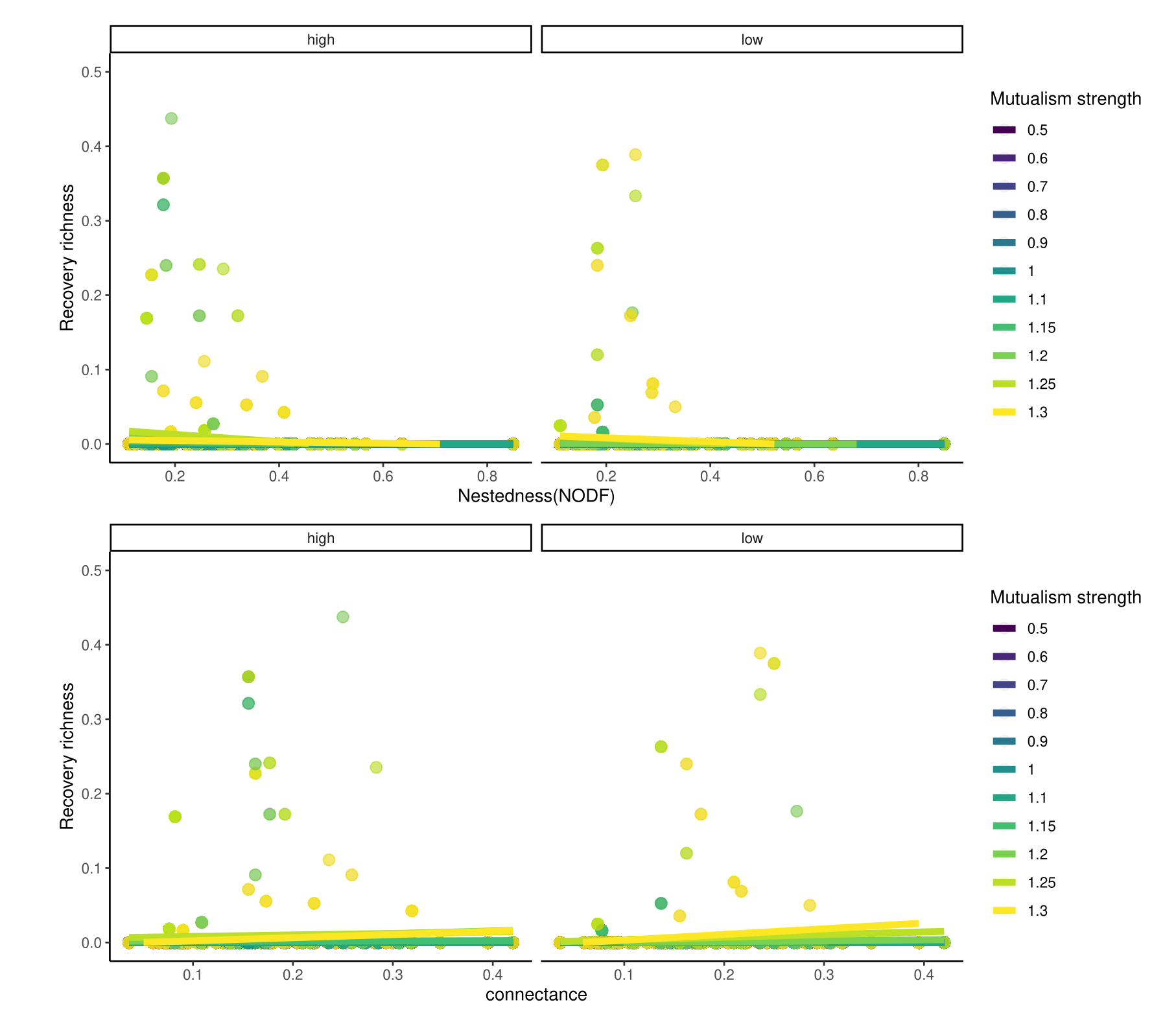

Supplement: S2 Fig — Recovery richness of networks as a function of Nestedness (top) and connectance (bottom) for a forcing strength ν of 0.5 for species that had degrees di ≤ 3. Note that when a specialist species is chosen to be perturbed, i.e., a species with low degree, networks did not resurrect. In the figure the faceted “high” and “low” meant high trait variation and low trait variation. Underlying data and R scripts for reproducing this figure can be found in https://doi.org/10.5281/zenodo.13598906. (TIF) [file pbio.3002826.s003.tif]

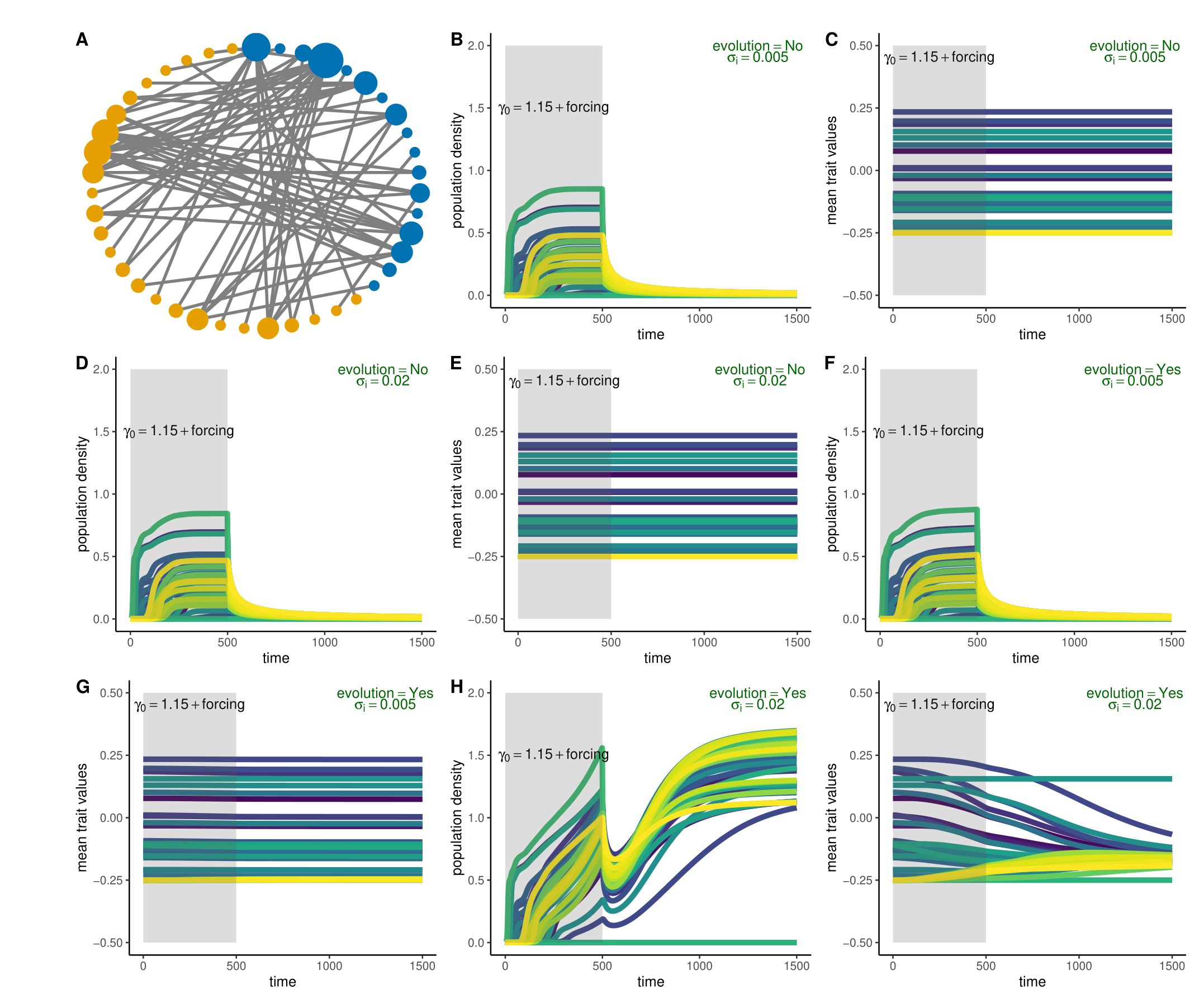

Supplement: S3 Fig — (A) A plant–pollinator network of 46 species. (B, C) Population dynamics and evolutionary trait dynamics of the 46 species network in the collapse regime of γ0 = 1.15, when trait variation σi = 0.005 was low, and evolution was turned off, i.e., hi2=0 for all species. Here in B and C, at γ0 = 1.15 only the most dominant species in terms of interactions was perturbed positively for a duration T of 500 time points with a strength, v = 0.5. Network recovery fails here. (D, E) When trait variation σi = 0.02 was high but evolution was still turned off, i.e., hi2=0, network resurrection fails. (F, G) When evolution was turned on i.e., hi2=0.4 but trait variation was low (σi = 0.005), network resurrection still fails. (H, I) Only when trait variation was moderate, i.e., σi = 0.02 and evolution turned on hi2=0.4, network resurrection in the collapse-regime from perturbing the most generalist species succeeds. Here in all the simulations, initial densities of all species Ni were sampled from a uniform random distribution of U[0, 0.005]. Forcing strength v = 0.5, duration of perturbation T = 500. Underlying data and R scripts for reproducing this figure can be found in https://doi.org/10.5281/zenodo.13598906. (TIF) [file pbio.3002826.s004.tif]

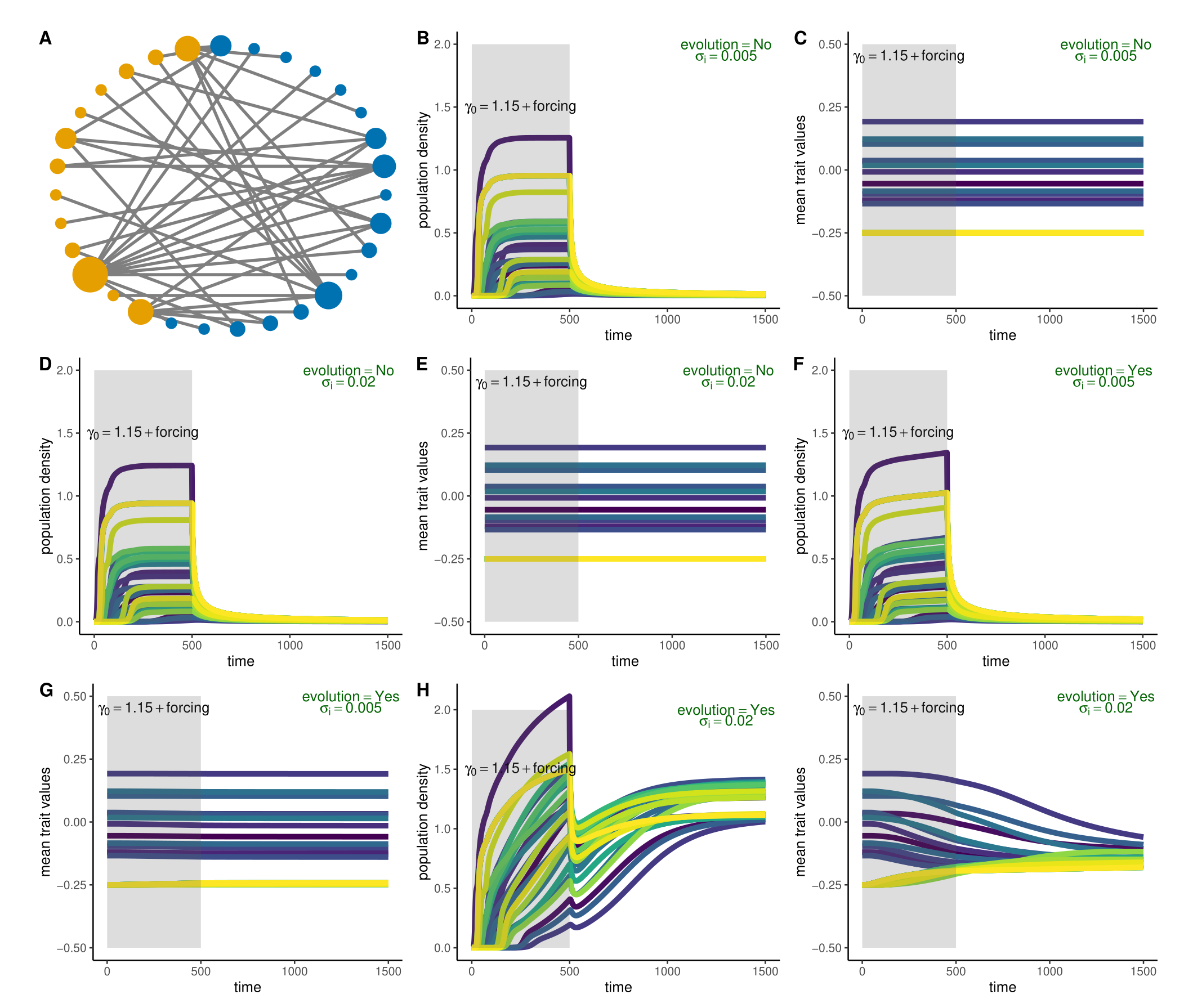

Supplement: S4 Fig — (A) A plant–pollinator network of 31 species. (B, C) Population dynamics and evolutionary trait dynamics of the 31 species network in the collapse regime of γ0 =1.15, when trait variation σi = 0.005 was low, and evolution was turned off, i.e., hi2=0 for all species. Here in B and C, at γ0 = 1.15 only the most dominant species in terms of interactions was perturbed positively for a duration T of 500 time points with a strength, v = 0.5. Network recovery fails here. (D, E) When trait variation σi = 0.02 was high but evolution was still turned off, i.e., hi2=0. Here, network resurrection fails. (F, G) When evolution was turned on, i.e., hi2=0.4 but trait variation was low (σi = 0.005), network resurrection still fails. (H, I) Only when trait variation was moderate, i.e., σi = 0.02 and evolution turned on hi2=0.4, network resurrection in the collapse-regime from perturbing the most generalist species succeeds. Here in all the simulations, initial densities of all species Ni were sampled from a uniform random distribution of U[0, 0.005]. Forcing strength v = 0.5, duration of perturbation T = 500. Underlying data and R scripts for reproducing this figure can be found in https://doi.org/10.5281/zenodo.13598906. (TIF) [file pbio.3002826.s005.tif]

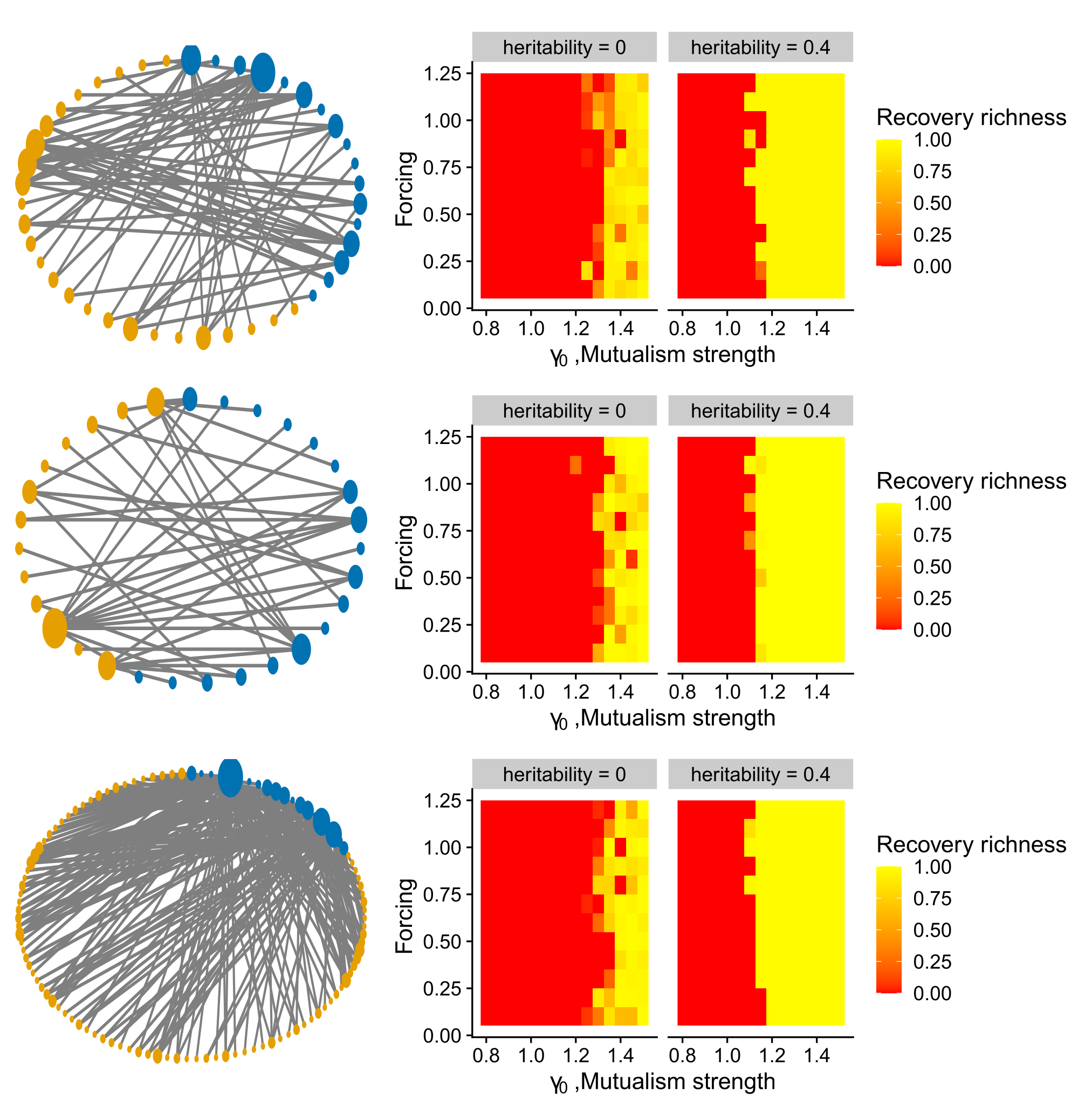

Supplement: S5 Fig — (Top) For a network with 49 species: Network revival when forcing the species with the highest number of interactions for a wide range of forcing strength, ν, and average mutualistic strength for 2 levels of heritability, hi2=0 and hi2=0.4. Even in the absence of heritable variation, i.e., the absence of evolutionary dynamics, network revival from a single species at unfavorable conditions could still be a possibility. (Middle) For a network with 34 species shown for a wide range of forcing strength ν and average mutualistic strength and for 2 levels of heritability, hi2=0 and hi2=0.4. (Bottom) For a network with 105 species shown here for a wide range of forcing strength, ν, and average mutualistic strength and for 2 levels of heritability, hi2=0 and hi2=0.4. Here in all the simulations, initial densities of all species Ni were sampled from a uniform random distribution of U[0, 0.005]. Duration of perturbation T = 500, and trait variation was fixed for all species at σi = 0.02. Underlying data and R scripts for reproducing this figure can be found in https://doi.org/10.5281/zenodo.13598906. (TIF) [file pbio.3002826.s006.tif]

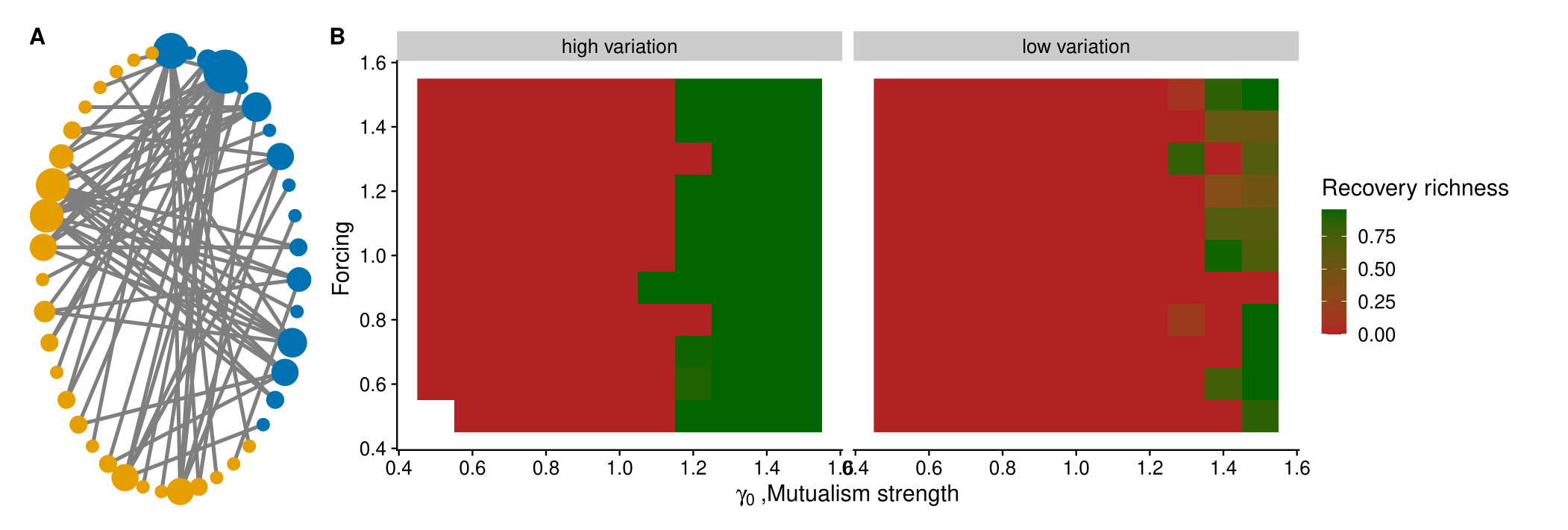

Supplement: S6 Fig — Here constant addition of density of 1.2 (forcing on y-axis) would mean that at constant rate a density of 1.2 of the most generalist species is added for a duration of 500 time points. High trait variation: networks recover fully at low γ0, whereas for low variation, networks recover only at higher γ0 values. Initial species density was below 0.005, mean trait values were sampled from Table 1 in the main text. Low variation σi = 0.005, and high variation σi = 0.02. Underlying data and R scripts for reproducing this figure can be found in https://doi.org/10.5281/zenodo.13598906. (TIF) [file pbio.3002826.s007.tif]

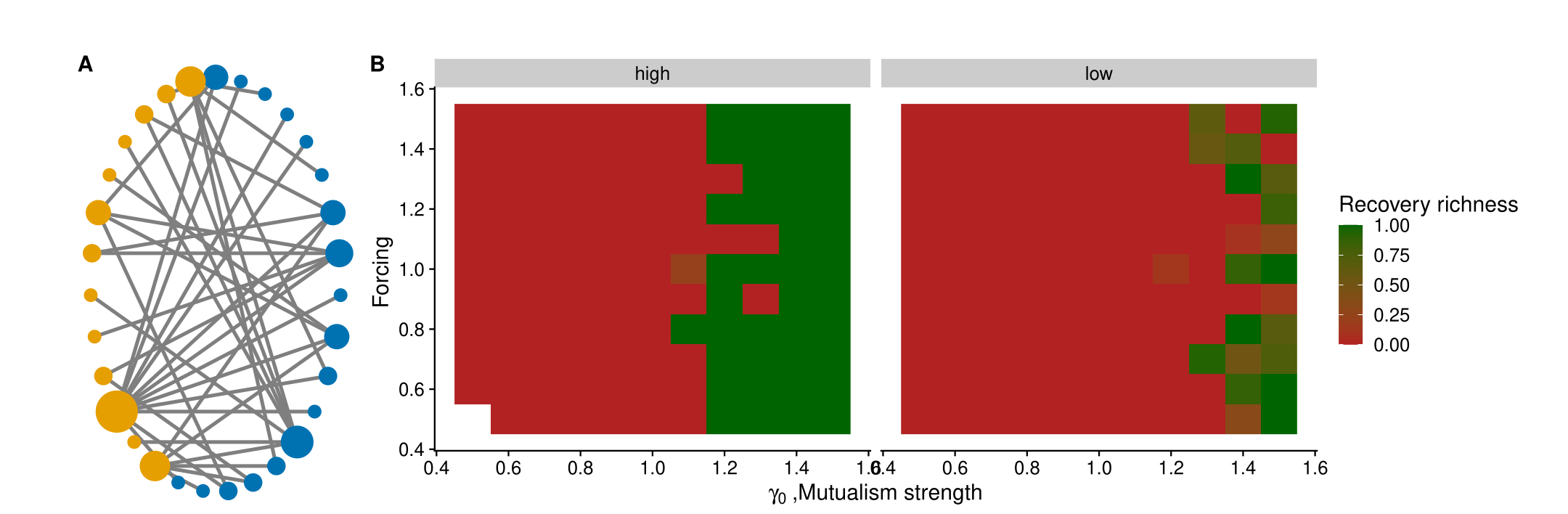

Supplement: S7 Fig — Here forcing of 1.2 would mean that at consecutive time points a density of 1.2 of the most generalist species is added for a duration of 500 time points. High trait variation: networks recover fully even at low γ0, whereas for low variation, networks recover only at higher γ0 values. Initial species density was below 0.005, mean trait values were sampled as from Table 1 in the main text. Low variation σi = 0.005 and high variation σi = 0.02. Underlying data and R scripts for reproducing this figure can be found in https://doi.org/10.5281/zenodo.13598906. (TIF) [file pbio.3002826.s008.tif]

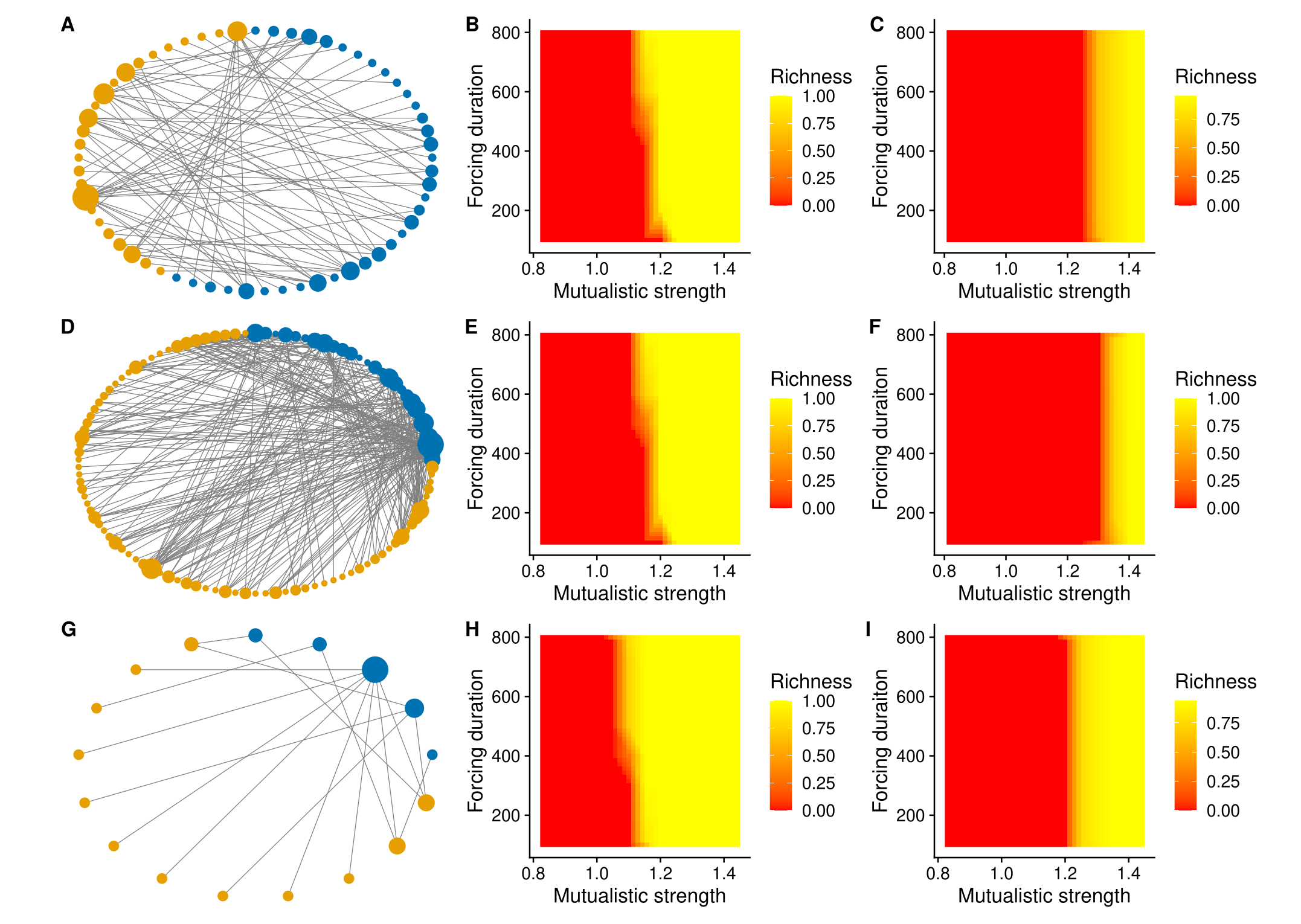

Supplement: S8 Fig — (A) A network of 61 species, connectance of 0.09, nestedness of 0.19, (D) a network of 101 species with connectance of 0.108 and nestedness of 0.221, and (G) a network of 17 species with a connectance of 0.288, nestedness of 0.292. In (B-E-H) high trait variation: networks recover readily as forcing duration increases even at low levels of γ0. (C-F-I) Low trait variation: same networks with low trait variation. Forcing strength was fixed at 0.5, initial species density was below 0.005, mean trait values were sample as from Table 1 in the main text. Low variance σi = 0.005 and high variance σi = 0.02. Underlying data and R scripts for reproducing this figure can be found in https://doi.org/10.5281/zenodo.13598906. (TIF) [file pbio.3002826.s009.tif]

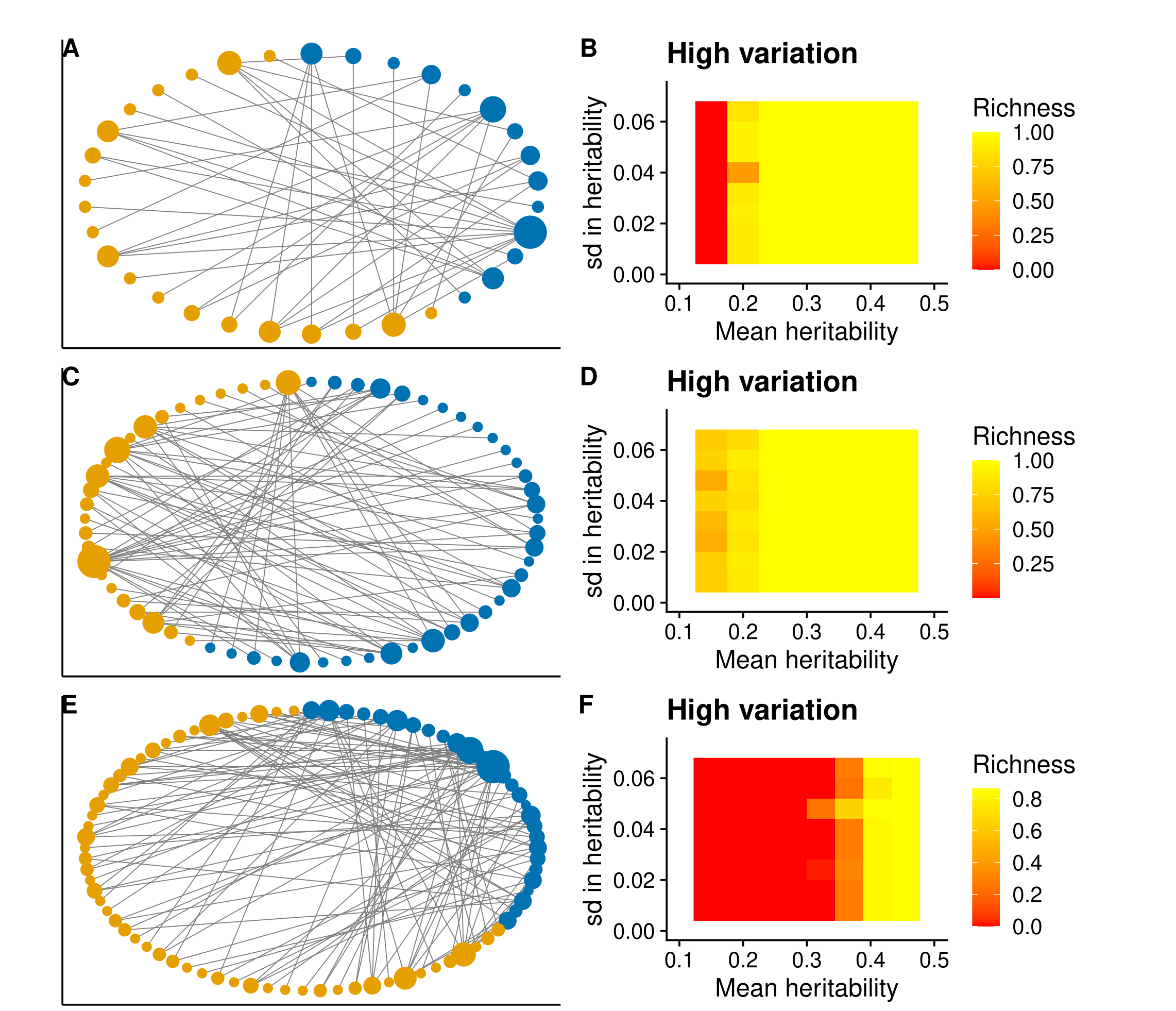

Supplement: S9 Fig — (A) A plant–pollinator network of 35 species. (B) Mean h2 had a larger impact than variance on recovery richness, when the most generalist species is perturbed for a certain duration. (C) A plant–pollinator network of 61 species. (D) A slightly higher h2 leads to full recovery of all species with variance in h2 having very small impact on the outcome. (E, F) Similarly for 101 species network, we observed similar outcomes, with variance in h2 having little impact. Forcing strength was fixed at 0.5, initial species density was below 0.005, mean trait values were sample as from Table 1 in the main text. High variance σi = 0.02. Underlying data and R scripts for reproducing this figure can be found in https://doi.org/10.5281/zenodo.13598906. (TIF) [file pbio.3002826.s010.tif]

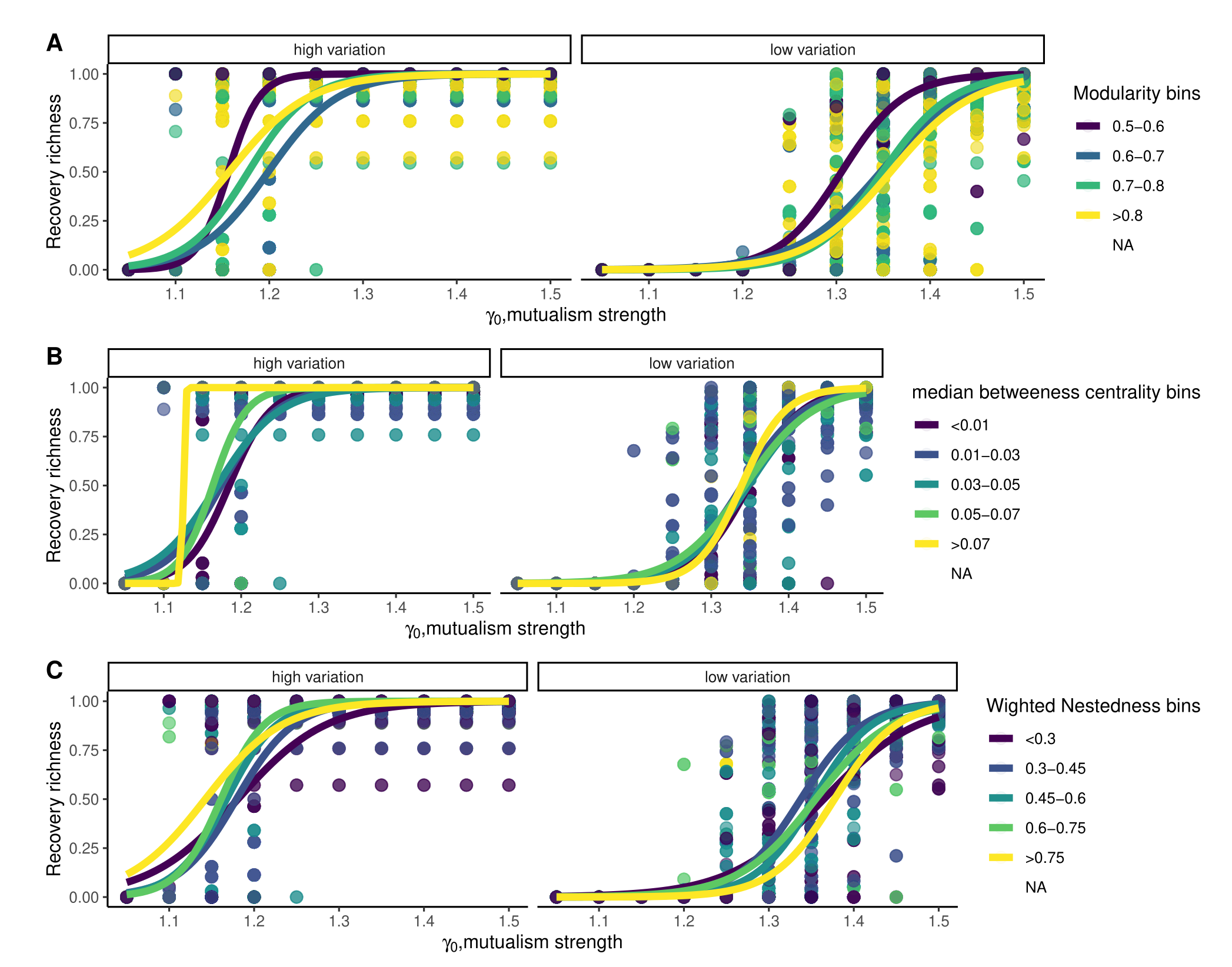

Supplement: S10 Fig — Network recovery from perturbing a single species was impacted moderately by network modularity (A), positively impacted by mean betweenness centrality in a network (B), and positively by weighted nestedness (C), particularly when species had higher trait variation. In each of these networks, only the species with the highest degree, was positively perturbed from a very low density, Ni < 0.005, for a duration of 500 time points with a forcing strength of 0.5, while the rest of the species remained unperturbed. Shown here are data from 115 networks. σi was fixed at 0.02 for the high trait variation case and 0.005 for the low trait variation case, respectively. Initial mean trait values were sampled according to parameter values given in Table 1. Different colored lines represent generalized linear model fitting with quasibinomial error distributions. Underlying data and R scripts for reproducing this figure can be found in https://doi.org/10.5281/zenodo.13598906. (TIF) [file pbio.3002826.s011.tif]

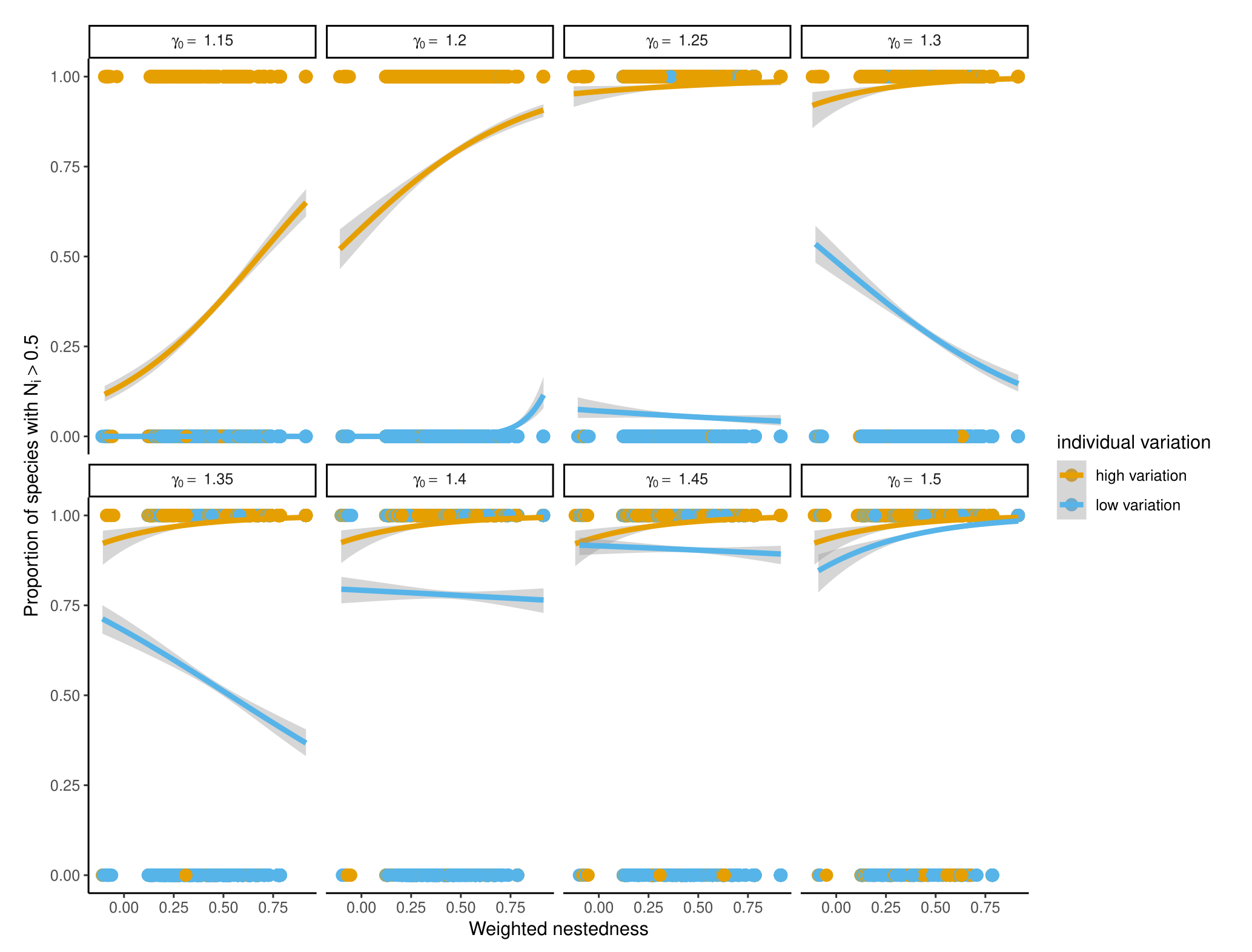

Supplement: S11 Fig — As nestedness increased proportion of species with density greater than 0.5 increased after species-specific perturbation was stopped and more so when species had high trait variation. To start with, the networks were collated from web-of-life database and species had an initial density of Ni < 0.005, and perturbation/forcing strength of 0.5 was applied to the species with the highest degree for a duration of 500 time points. For high trait variation σi for all species was fixed at 0.02. Initial mean trait values were sampled as given in Table 1. Different colored lines with confidence interval represent generalized linear model fitting with quasibinomial error distributions. Underlying data and R scripts for reproducing this figure can be found in https://doi.org/10.5281/zenodo.13598906. (TIF) [file pbio.3002826.s012.tif]

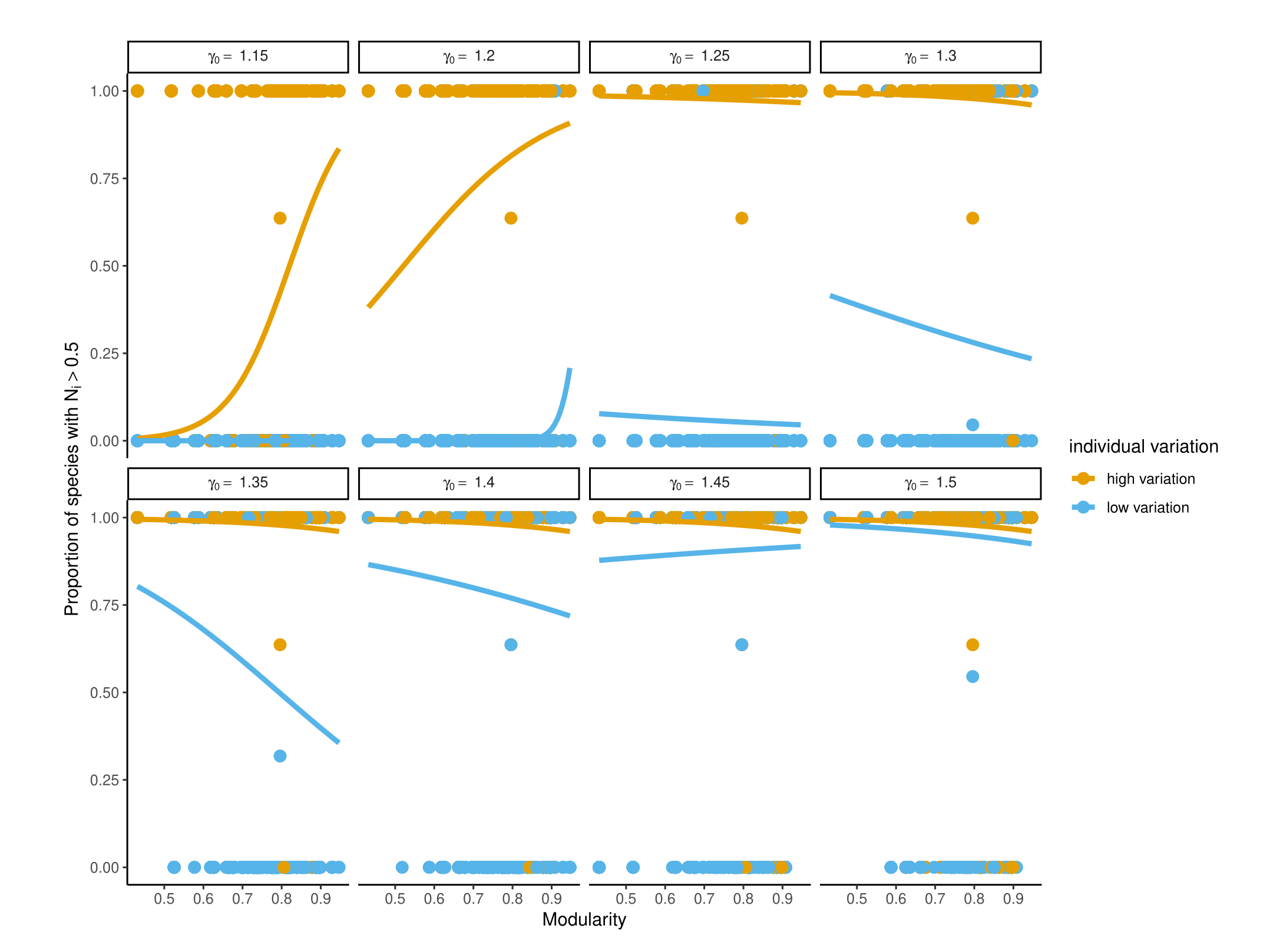

Supplement: S12 Fig — There was no specific relationship of network modularity and proportion of species with density greater than 0.5 after species-specific perturbation was stopped for 2 levels of trait variation. To start with, the networks were collated from web-of-life database and species had an initial density of Ni < 0.005, and perturbation/forcing strength of 0.5 was applied to the species with the highest degree for a duration of 500 time points. For high trait variation σi for all species was fixed at 0.02. Initial mean trait values were sampled as given in Table 1. Different colored lines with confidence interval represent generalized linear model fitting with quasibinomial error distributions. Underlying data and R scripts for reproducing this figure can be found in https://doi.org/10.5281/zenodo.13598906. (TIF) [file pbio.3002826.s013.tif]

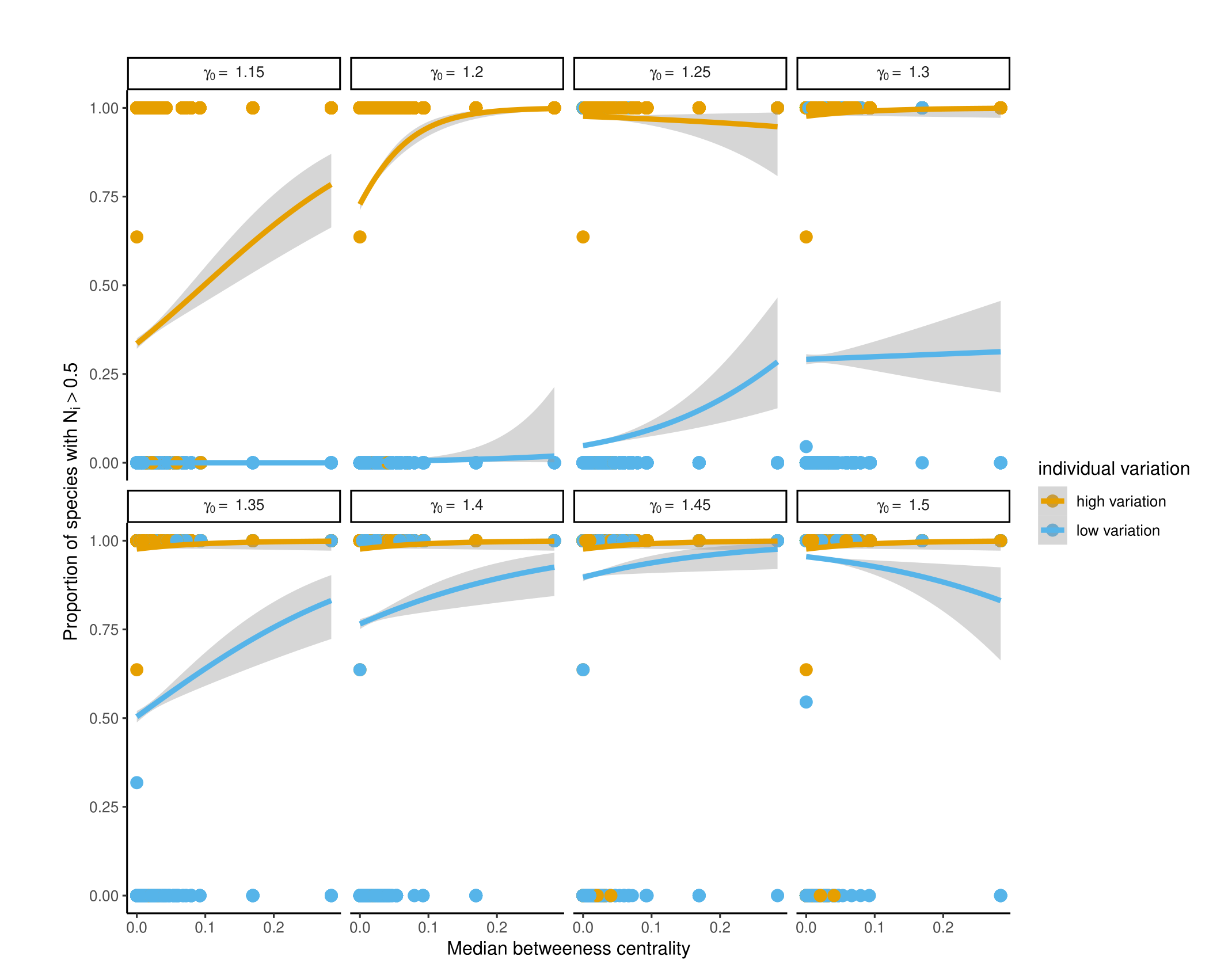

Supplement: S13 Fig — At low levels of γ0<1.25, particularly for high trait variation, low median betweenness centrality would lead to low proportion of species with density greater than 0.5 after species-specific perturbation was stopped. We observe similar results for low trait variation but for γ0 of 1.24, 1.3, and 1.35. At levels of γ0>1.2, networks are already in the recovery parameter range with species-specific perturbation. To start with, the networks were collated from web-of-life database and species had an initial density of Ni < 0.005, and perturbation/forcing strength of 0.5 was applied to the species with the highest degree for a duration of 500 time points. For high trait variation σi for all species was fixed at 0.02. Initial mean trait values were sampled as given in Table 1. Different colored lines with confidence interval represent generalized linear model fitting with quasibinomial error distributions. Underlying data and R scripts for reproducing this figure can be found in https://doi.org/10.5281/zenodo.13598906. (TIF) [file pbio.3002826.s014.tif]

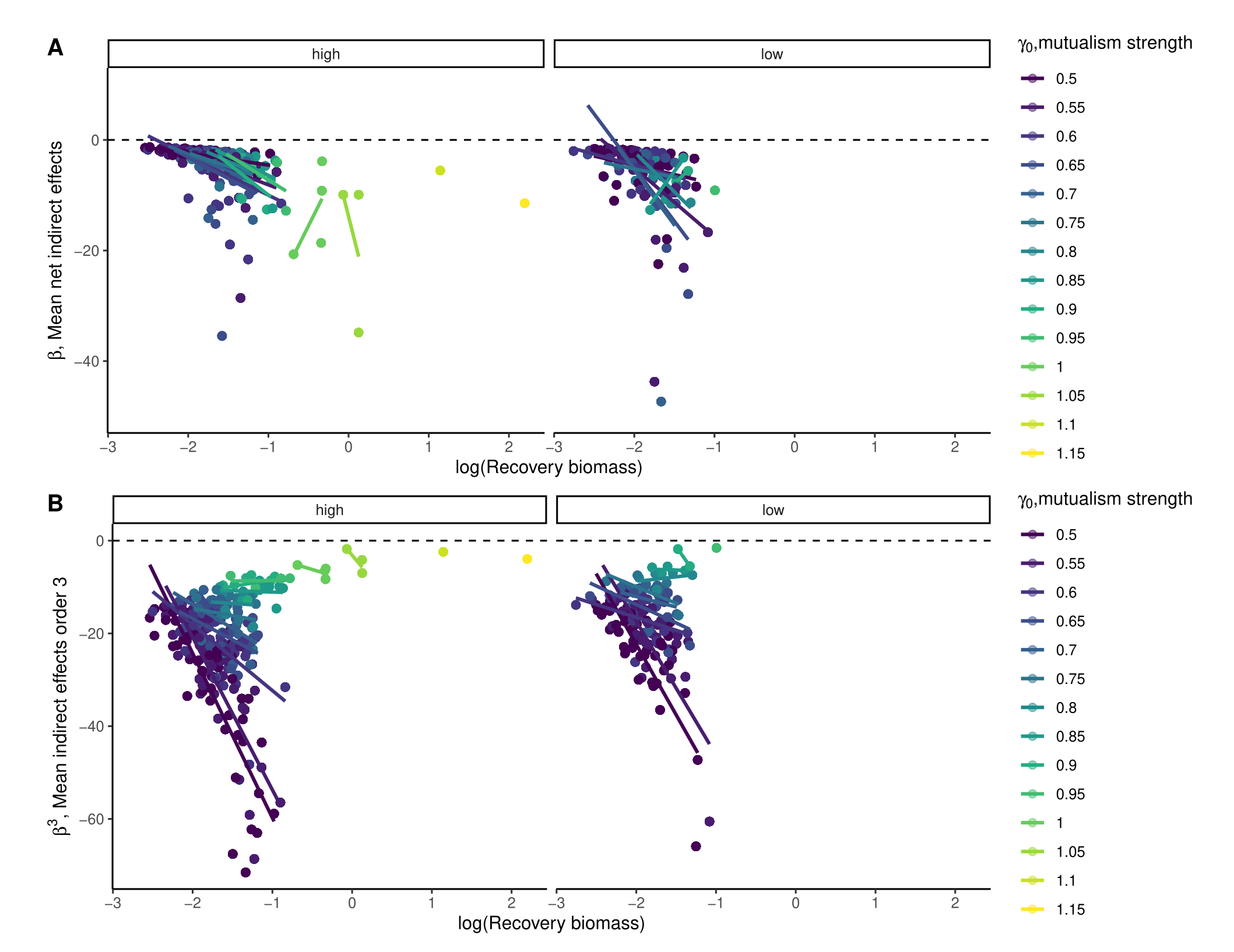

Supplement: S14 Fig — (A) Mean net indirect effects, i.e., β, was below zero across different γ0 and for all matrices with spectral radius less than 1. (B) Similarly, mean indirect effects of order 3, β3, was also negative across different levels of γ0, and decreased slightly as recovery biomass increased. As recovery biomass slightly increased, β3, became less negative. Underlying data and R scripts for reproducing this figure can be found in https://doi.org/10.5281/zenodo.13598906. (TIF) [file pbio.3002826.s015.tif]

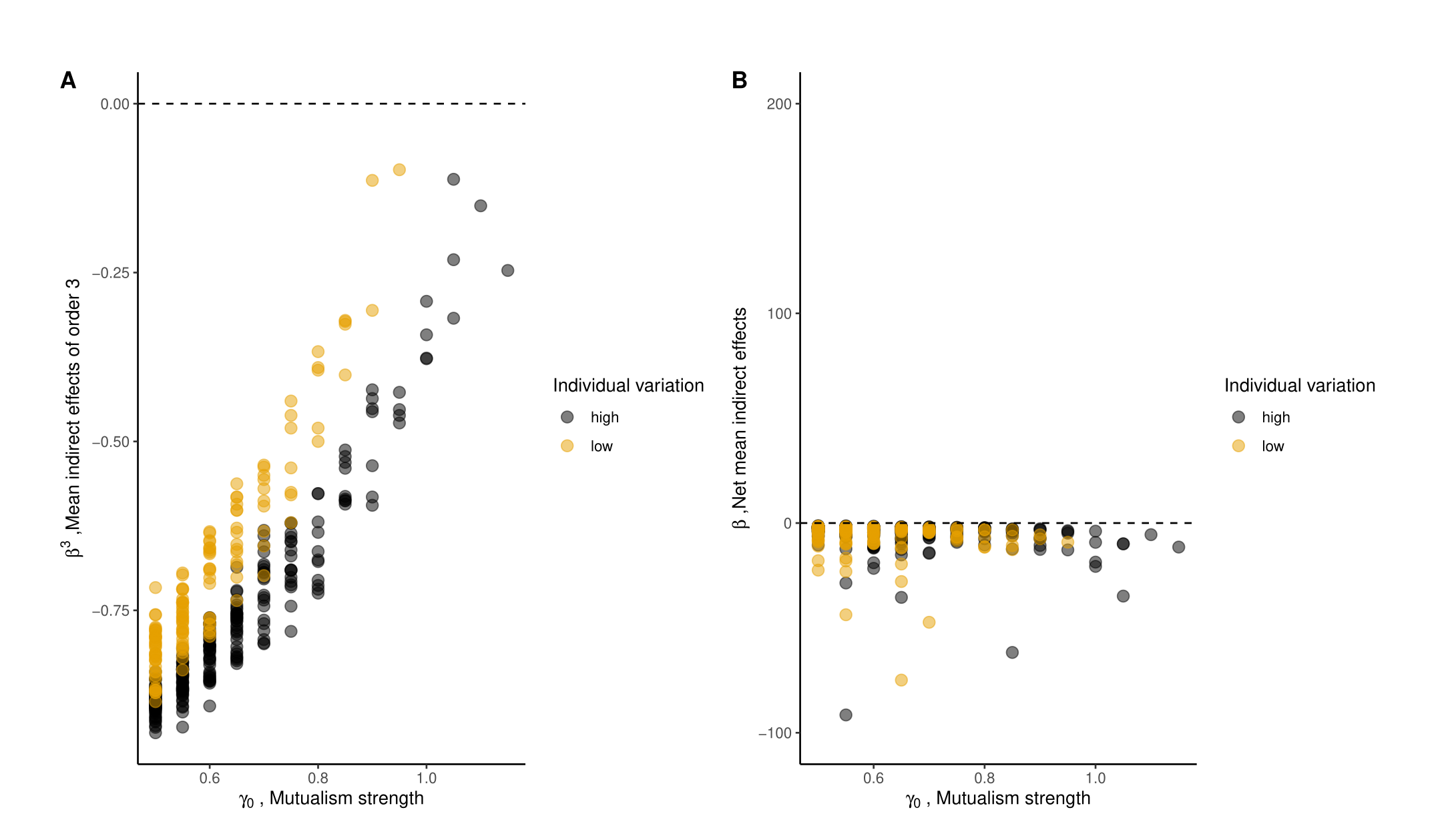

Supplement: S15 Fig — (A) Mean indirect effects of order 3, β3. At low γ0, mean indirect effects are mostly negative and remains below 0, but became less negative as γ0 increased indicating a positive impact of higher γ0. (B) Similarly, net mean indirect effects of all orders, β, in relation to γ0. Net indirect effects are mostly below zero. To be noted that indirect effects shown here fulfills the condition of equation 11 in S1 Text, and it happens when spectral radius of net interactions are less than 1. Underlying data and R scripts for reproducing this figure can be found in https://doi.org/10.5281/zenodo.13598906. (TIF) [file pbio.3002826.s016.tif]

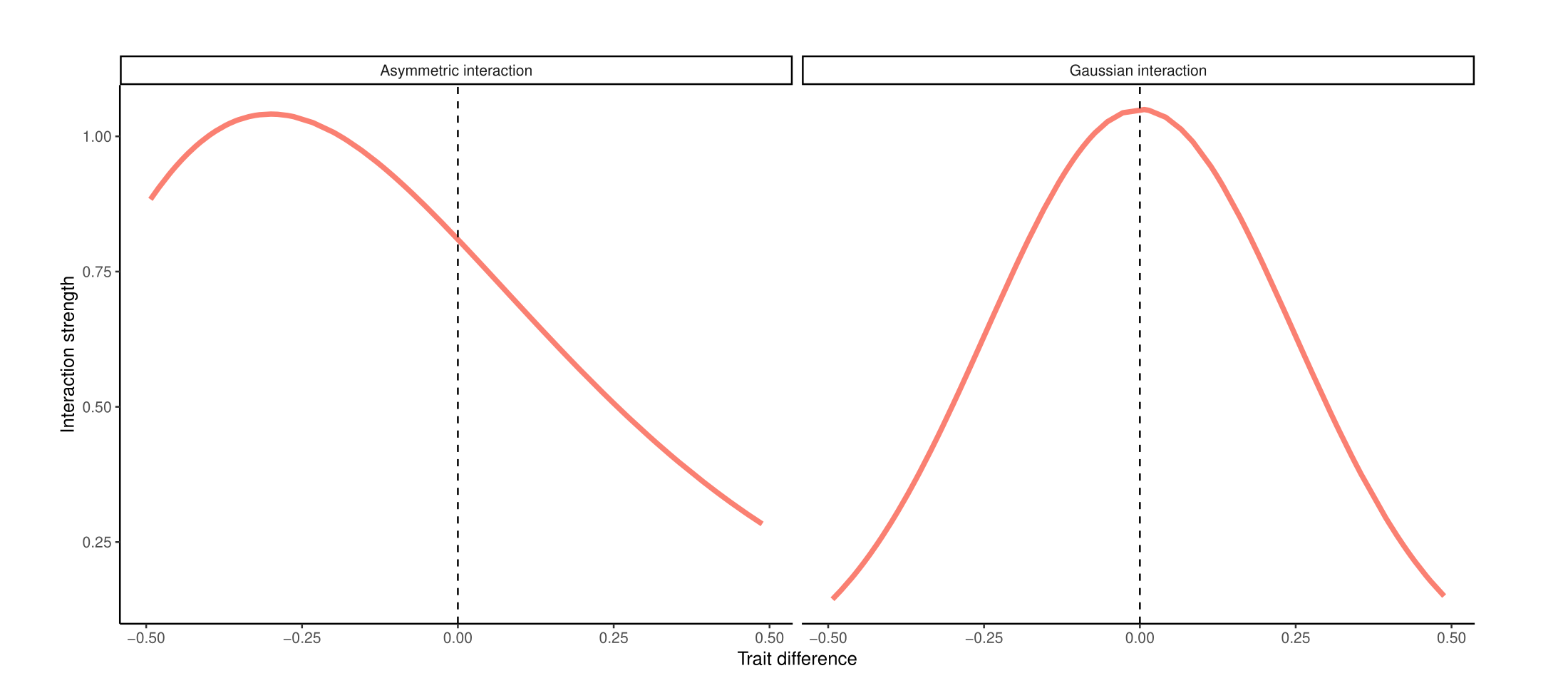

Supplement: S16 Fig — Left: γ(z,z′)=10Γ(((z−z′)/W+10),4.5,0.5), where an asymmetric function was given as Γ(Z,α1,β1) is the probability density function of a gamma distribution with shape parameter given by α1, and rate parameter given by β1. Here W = 0.1. Right: the Gaussian interactioin kernel given in the main text. Underlying data and R scripts for reproducing this figure can be found in https://doi.org/10.5281/zenodo.13598906. (TIF) [file pbio.3002826.s017.tif]

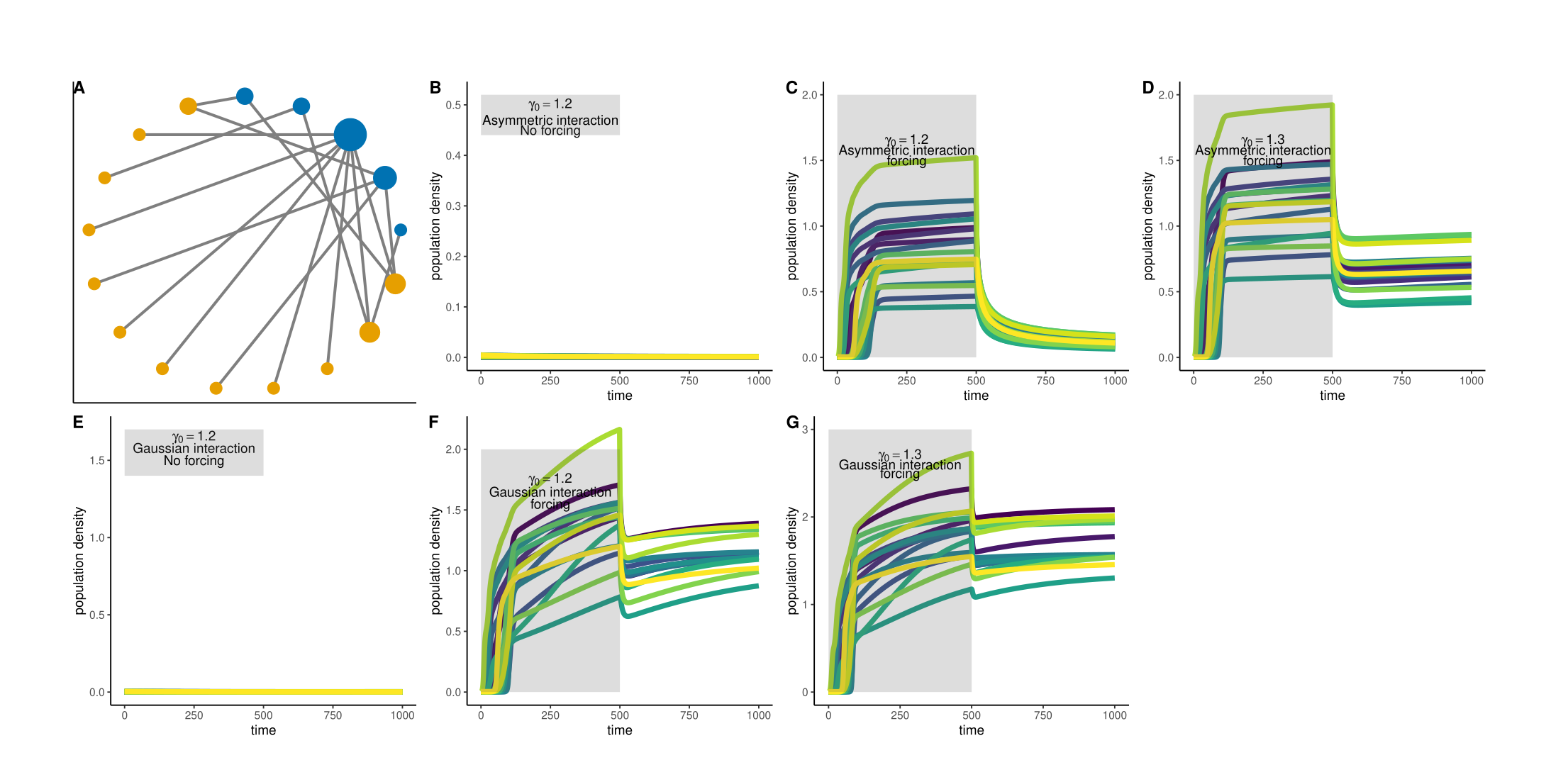

Supplement: S17 Fig — (B) When there is no species specific forcing, at γ0 = 1.2, the network remains at the undesirable state. (C) At forcing strength of 0.5 for the species with the highest degree, and with species interaction dictated by a asymmetric kernel, we do not observe network recovery after the perturbation was stopped at 500 time points. (D) However, at γ0 = 1.3, we do see a network recovery, but the overall biomass achieved was low. (E) Same network, with Gaussian kernel, without forcing, the network remains at the undesirable state. (F) However, at γ0 = 1.2, and species specific forcing for a duration of 500 time points, we see the network recover, while achieving slightly higher biomass than the case of asymmetric species interaction. (G) At higher γ0 if 1.3, the same network with Gaussian interaction kernel readily recovers with an overall higher biomass on average. In all these simulations, we considered initial starting species densities to be Ni < 0.005 and trait variation for all species to be at a moderate level of σi = 0.02. Underlying data and R scripts for reproducing this figure can be found in https://doi.org/10.5281/zenodo.13598906. (TIF) [file pbio.3002826.s018.tif]

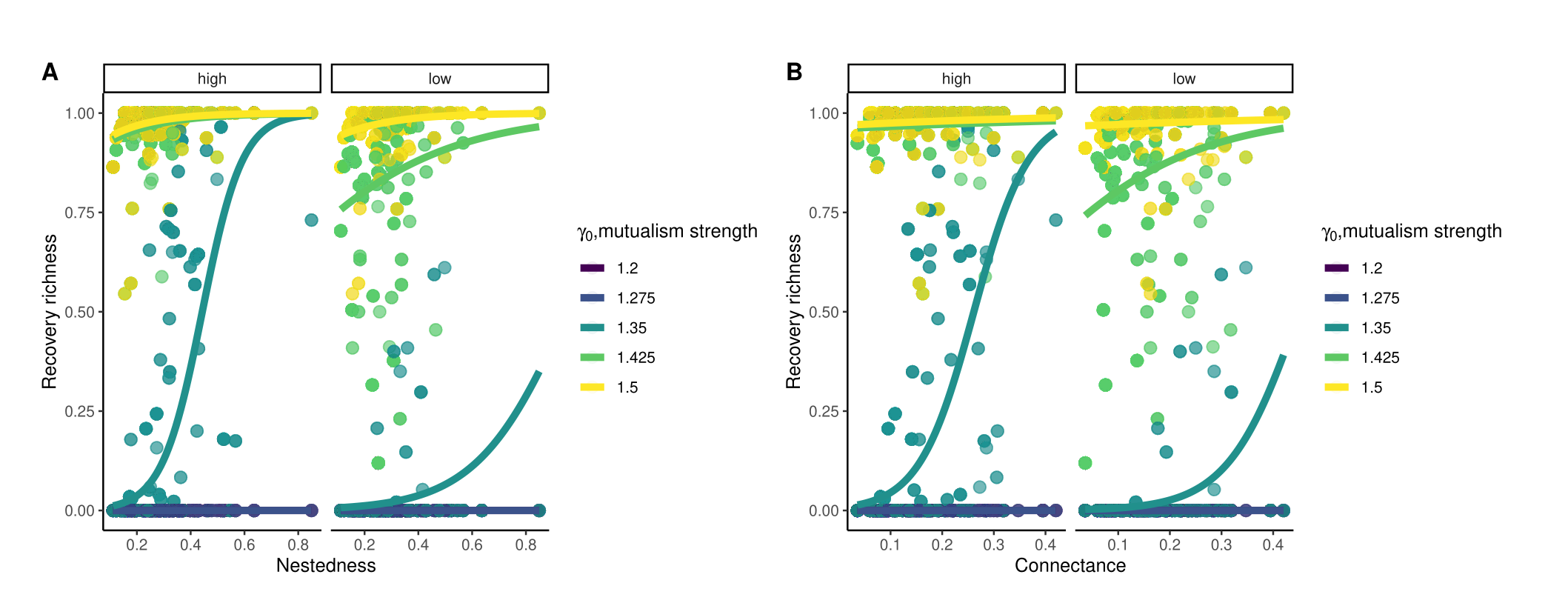

Supplement: S18 Fig — Network recovery from perturbing a single species was impacted positively by positively by nestedness (A), and connectance (B) shown when species interactions were asymmetric in nature, for 5 different thresholds of γ0. In each of these networks, only the species with the highest degree, was positively perturbed from a very low density, Ni < 0.005, for a duration of 500 time points with a forcing strength of 0.5, while the rest of the species remained unperturbed. Shown here are data from 115 networks. σi was fixed at 0.02 for the high trait variation case and 0.005 for the low trait variation case respectively. Initial mean trait values were sampled according to parameter values given in Table 1 in main text. In all these simulations, we considered initial starting species densities to be Ni < 0.005 and trait variation for all species to be at a moderate level of σi = 0.02. We observe that network recovery is delayed and was achieved at only higher thresholds of mutualistic strength γ0>1.25. Different colored lines represent generalized linear model fitting with quasibinomial error distributions. Underlying data and R scripts for reproducing this figure can be found in https://doi.org/10.5281/zenodo.13598906. (TIF) [file pbio.3002826.s019.tif]

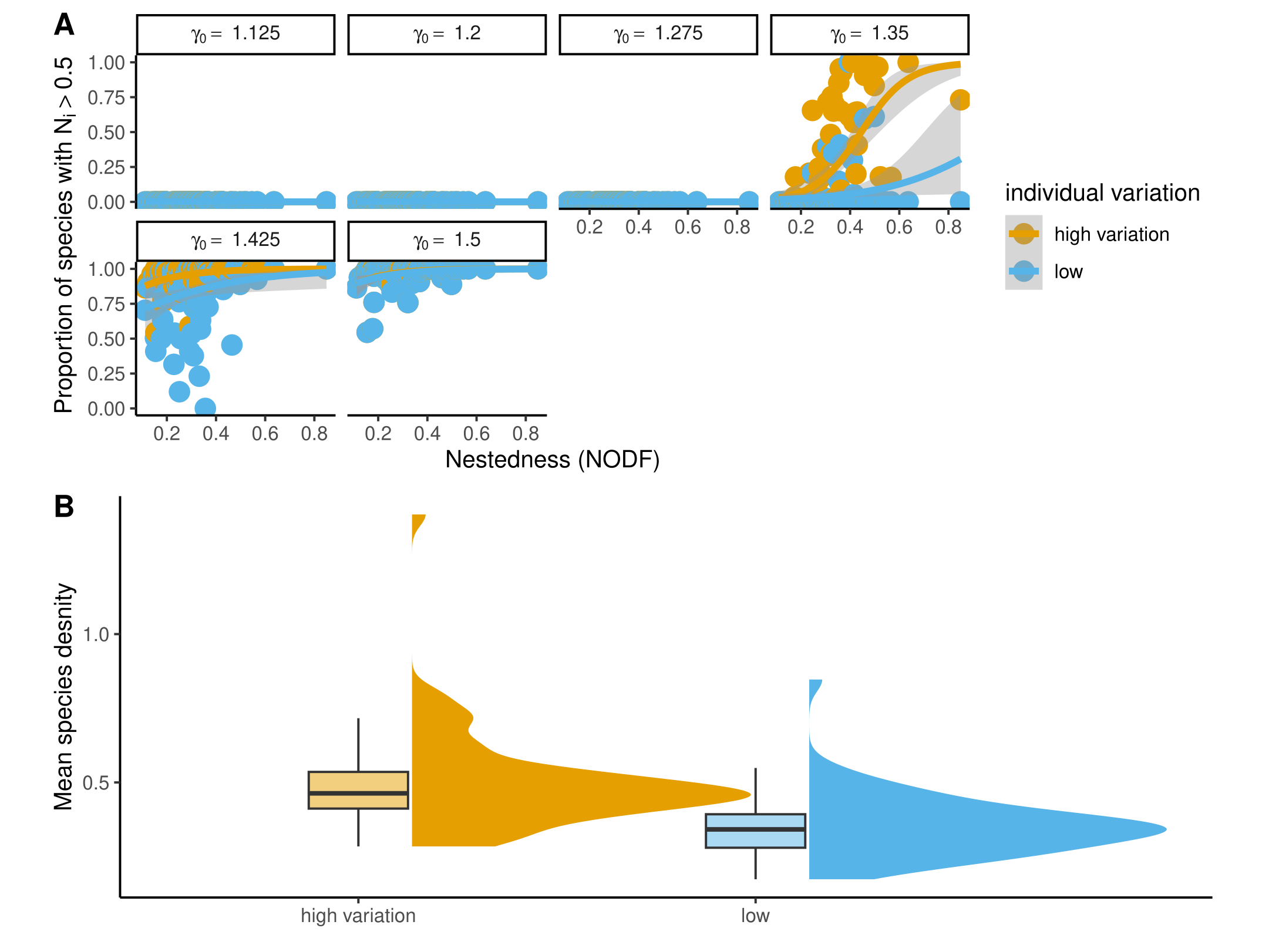

Supplement: S19 Fig — (A) As nestedness increased proportion of species with density greater than 0.5 increased after species-specific perturbation was stopped and more so when species had high trait variation, but the threshold at which species achieved densities higher than 0.5 was only at γ0>1.25, which was not the case when species interacted in Gaussian manner. (B) On average, mean pollinator density across all networks was high when species had high trait variation after recovery for γ0>1. In (A) to start with, the networks were collated from web-of-life database and species had an initial density of Ni < 0.005, and perturbation/forcing strength of 0.5 was applied to the species with the highest degree for a duration of 500 time points. Different colored lines with confidence interval represent generalized linear model fitting with quasibinomial error distributions. Note that in contrast to Fig 4 in main text, asymmetric species interaction impacts network recovery. Initial mean trait values were sampled as given in Table 1. Underlying data and R scripts for reproducing this figure can be found in https://doi.org/10.5281/zenodo.13598906. (TIF) [file pbio.3002826.s020.tif]

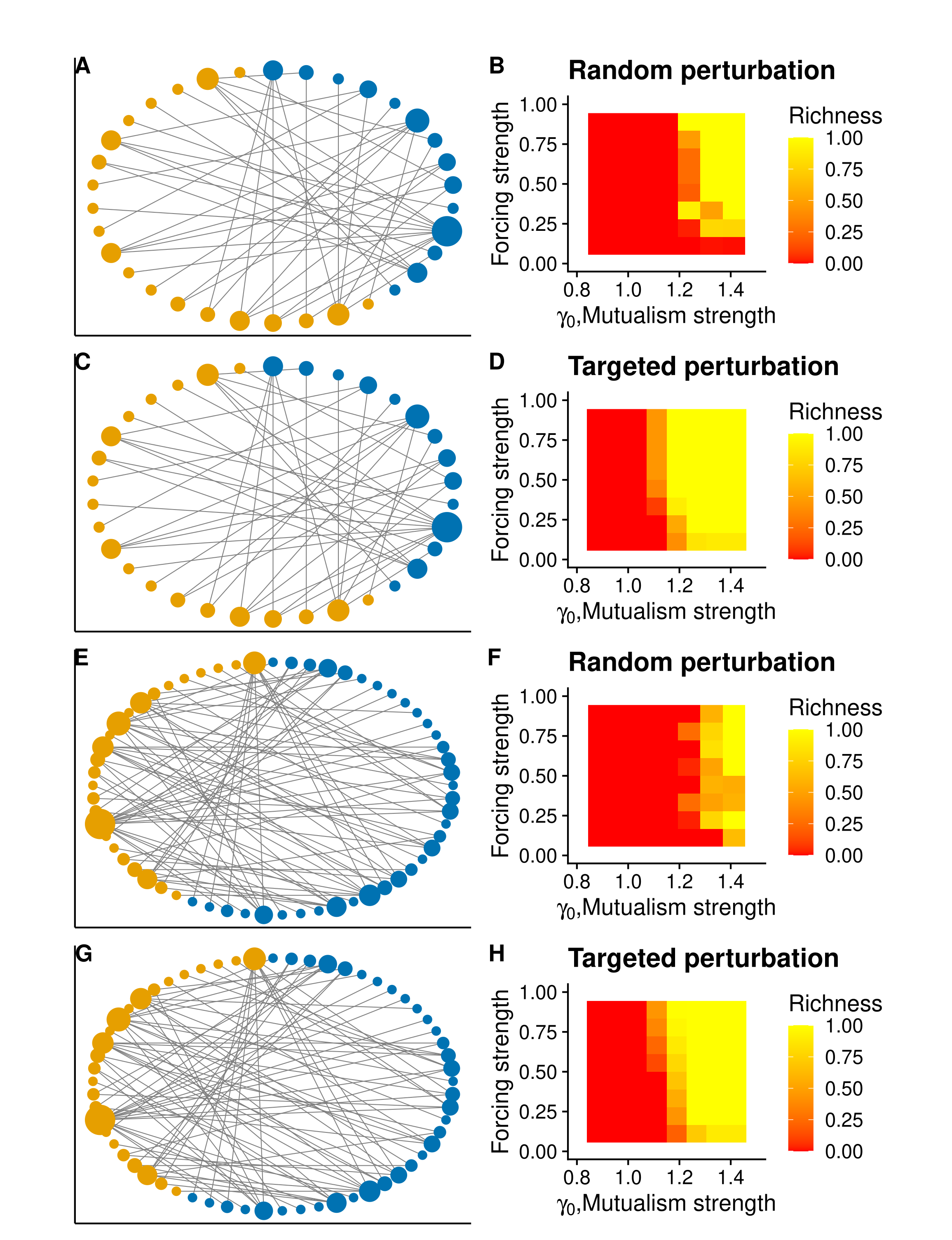

Supplement: S20 Fig — (A, B) The parameter space for successful resurrection for a network with 63 species differed but with a random perturbation of a species for different levels of forcing strength and γ0. Only at high threshold γ0>1.3 we observed complete recovery but at high forcing strength of 0.5. (C, D) For the same network, however, a targeted species-specific perturbation by selecting 2 species that had the highest betweenness centrality score. When perturbing this 2 specific species, we observed signficant differences in network recovery even at low levels of γ0<1.1. (E, F) For a slightly larger network with 102 species, we observed similar results for random species perturbation and for targeted perturbation (G, H). Targeted perturbation of 2 species based on betweenness centrality scores outperformed random targeting of species in a network. In all these simulations, we considered initial starting species densities to be Ni < 0.005 and trait variation for all species to be at a moderate level of σi = 0.02. Underlying data and R scripts for reproducing this figure can be found in https://doi.org/10.5281/zenodo.13598906. (TIF) [file pbio.3002826.s021.tif]

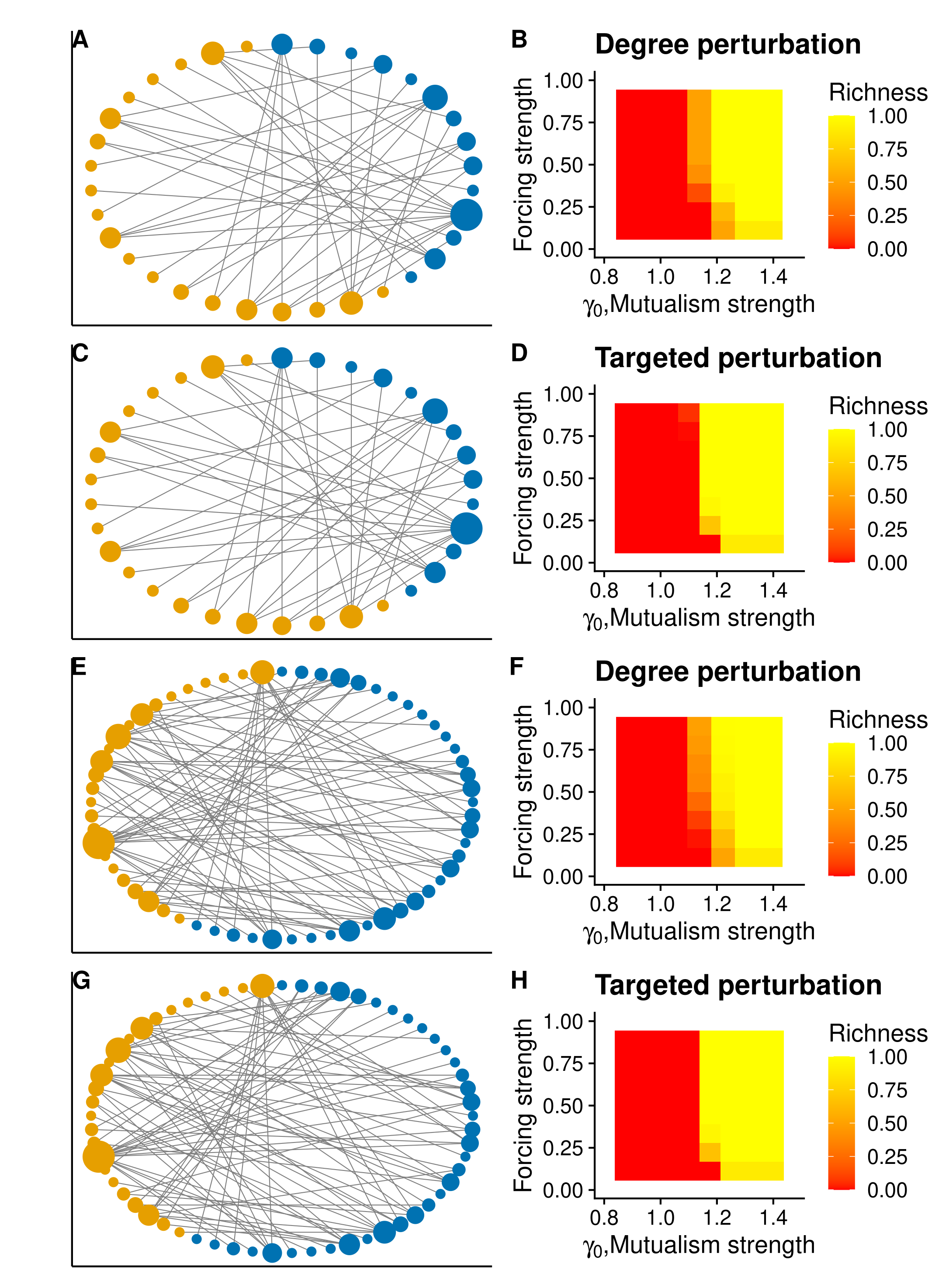

Supplement: S21 Fig — (A–D) The parameter space for successful resurrection for a network with 63 species slightly differed between targeted and degree-based perturbation for different levels of forcing strength and γ0. Only at high threshold γ0>1.3 we observed complete recovery but at high forcing strength of 0.5. (E–H) For a slightly larger network with 102 species, we observed similar results for targeted and degree-based species perturbation. Targeted perturbation of 2 species based on betweenness centrality scores and degree-based perturbation only applied to one single species with the highest degree. In all these simulations, we considered initial starting species densities to be Ni < 0.005 and trait variation for all species to be at a moderate level of σi = 0.02. Underlying data and R scripts for reproducing this figure can be found in https://doi.org/10.5281/zenodo.13598906. (TIF) [file pbio.3002826.s022.tif]

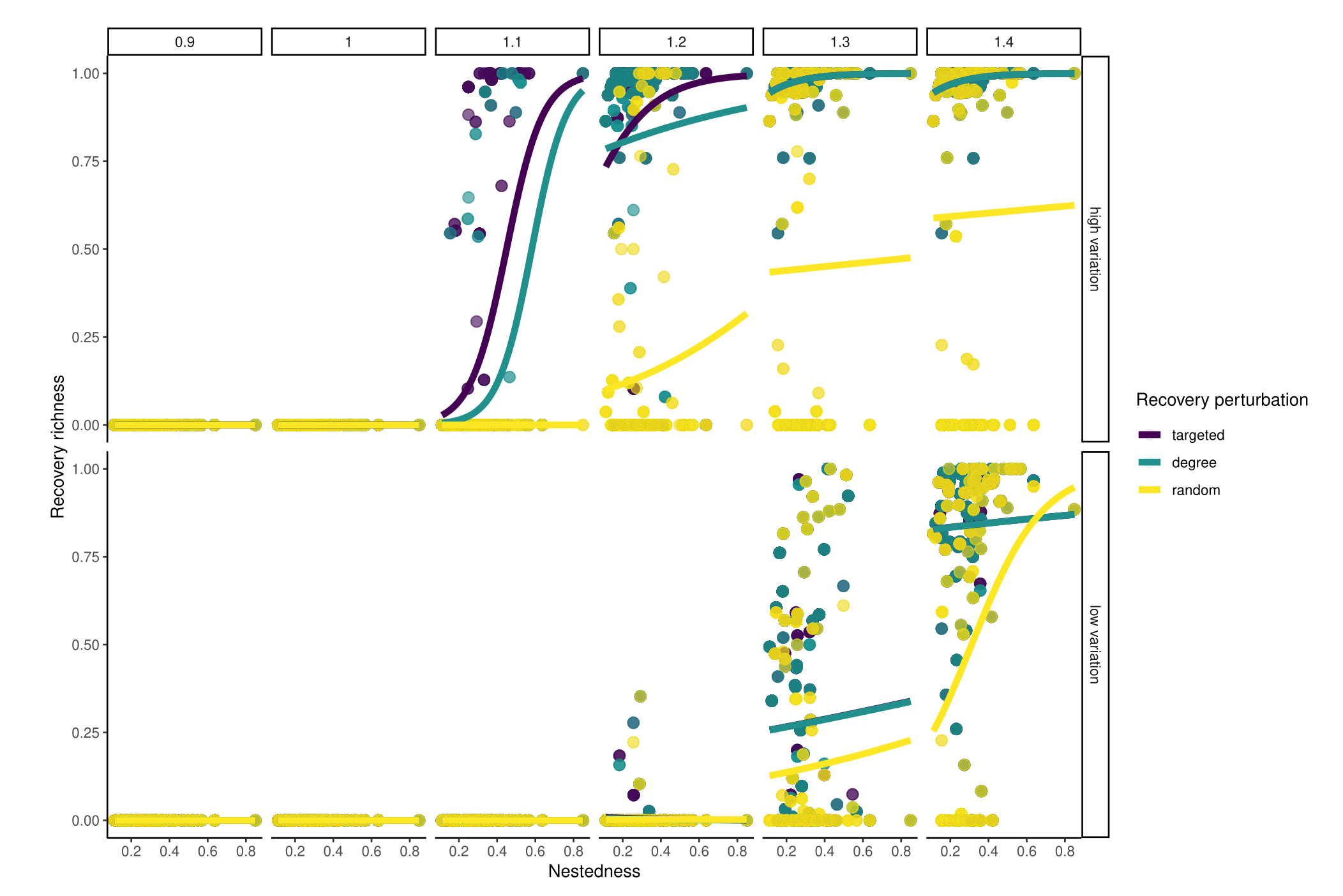

Supplement: S22 Fig — Degree-based was based on perturbation that is used in the main text. Random perturbation entailed randomly choosing a species in a network and perturbing the species for 500 time points for a forcing strength of 0.5. Finally, targeted perturbation entailed choosing 2 species based on betweenness centrality measure and perturbing those 2 species for a period of 500 time points with strength of 0.5. As nestedness (NODF) increased proportion of species with density greater than 0.5 increased specifically for targeted and degree-based. To start with, the networks were collated from web-of-life database and species had an initial density of Ni < 0.005, and perturbation/forcing strength of 0.5 was applied to the species with the highest degree for a duration of 500 time points. For high trait variation σi for all species was fixed at 0.02. Initial mean trait values were sampled as given in Table 1. Different colored lines representing generalized linear model fitting with quasibinomial error distributions. Underlying data and R scripts for reproducing this figure can be found in https://doi.org/10.5281/zenodo.13598906. (TIF) [file pbio.3002826.s023.tif]

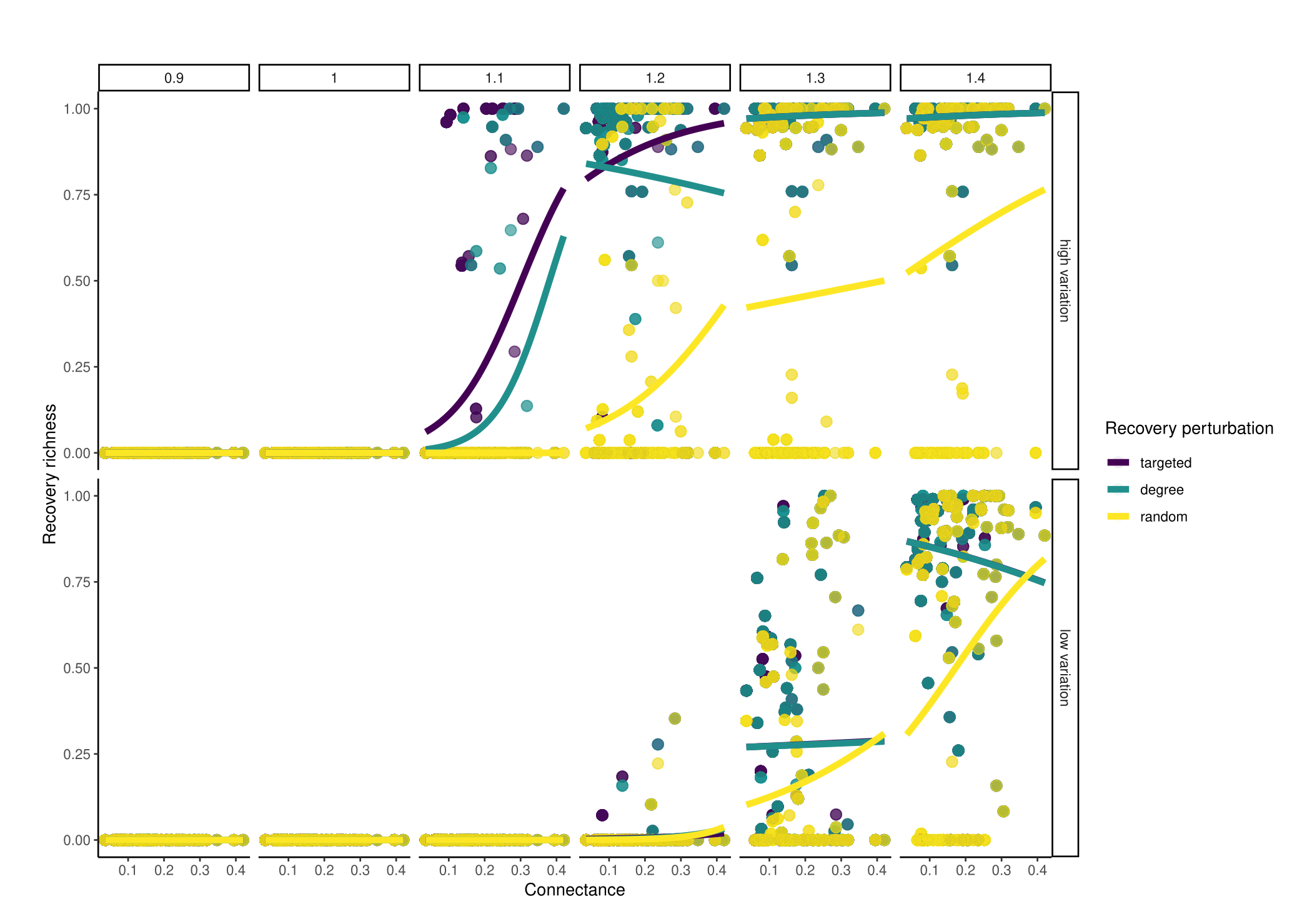

Supplement: S23 Fig — Degree-based was based on perturbation that is used in the main text. Random perturbation entailed randomly choosing a species in a network and perturbing the species for 500 time points for a forcing strength of 0.5. Finally, targeted perturbation entailed choosing 2 species based on betweenness centrality measure and perturbing those 2 species for a period of 500 time points with strength of 0.5. As nestedness (NODF) increased proportion of species with density greater than 0.5 increased specifically for targeted and degree-based. To start with, the networks were collated from web-of-life database and species had an initial density of Ni < 0.005, and perturbation/forcing strength of 0.5 was applied to the species with the highest degree for a duration of 500 time points. For high trait variation σi for all species was fixed at 0.02. Initial mean trait values were sampled as given in Table 1. Different colored lines representing generalized linear model fitting with quasibinomial error distributions. Underlying data and R scripts for reproducing this figure can be found in https://doi.org/10.5281/zenodo.13598906. (TIF) [file pbio.3002826.s024.tif]

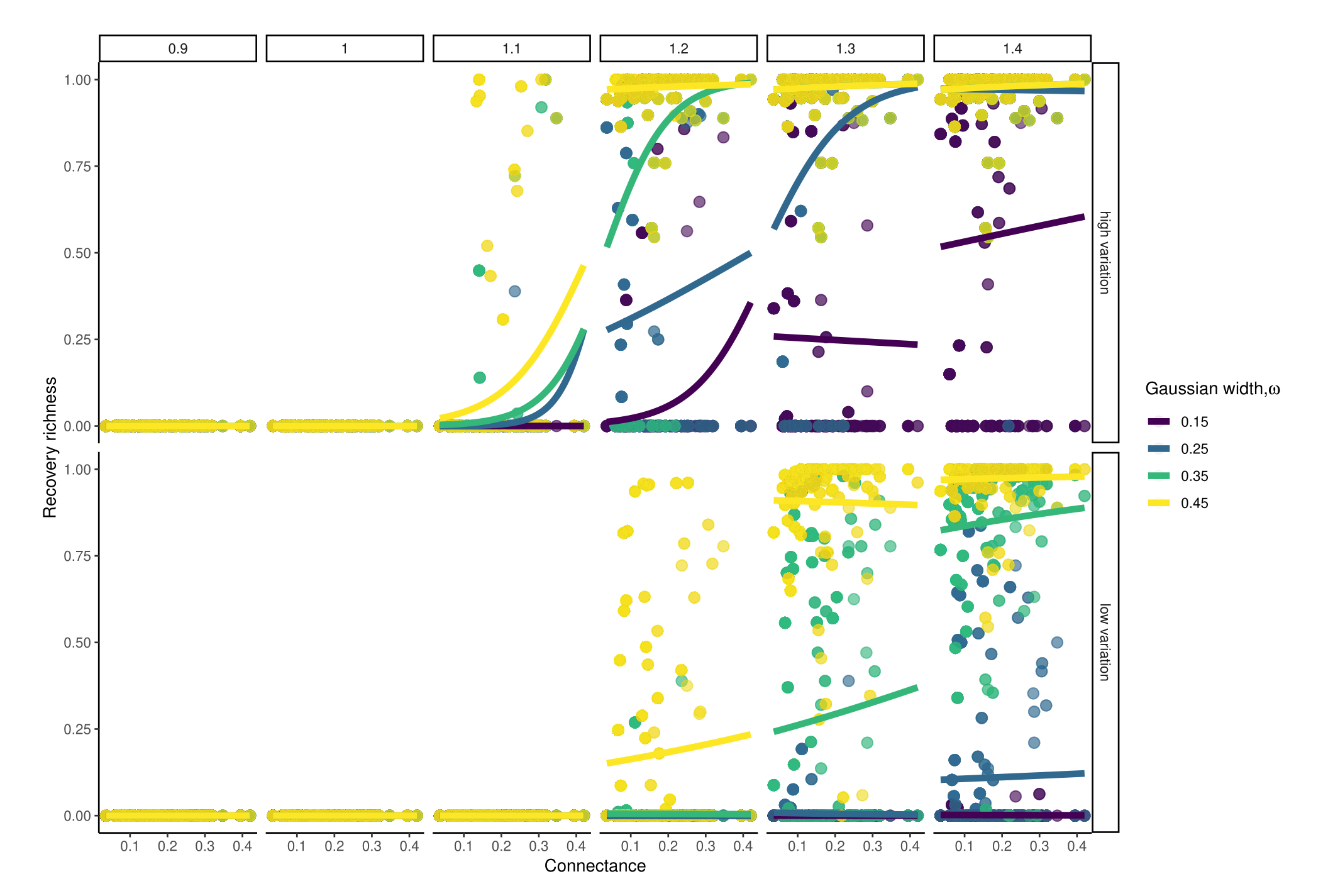

Supplement: S24 Fig — Network recovery was positively impacted by nestedness shown for 2 levels of trait variation (high variation and low variation), for 4 levels of mutualistic strength, γ0, of 0.9, 1, 1.1, 1.2, 1.3, 1.4, and for 4 different Gaussian function width ω. In all these networks, only the species with the highest degree, was positively perturbed from a very low density, Ni < 0.005, for a duration of 500 time points with a forcing strength of 0.5, while the rest of the species remained unperturbed. Shown here are data from 115 networks. σi was fixed at 0.02 for the high trait variation case and 0.005 for the low trait variation case, respectively. We observed that higher Gaussian kernel width, ω>0.15, leads to higher recovery. Initial mean trait values were sampled according to parameter values given in Table 1. Different colored lines representing generalized linear model fitting with quasibinomial error distributions. Underlying data and R scripts for reproducing this figure can be found in https://doi.org/10.5281/zenodo.13598906. (TIF) [file pbio.3002826.s025.tif]

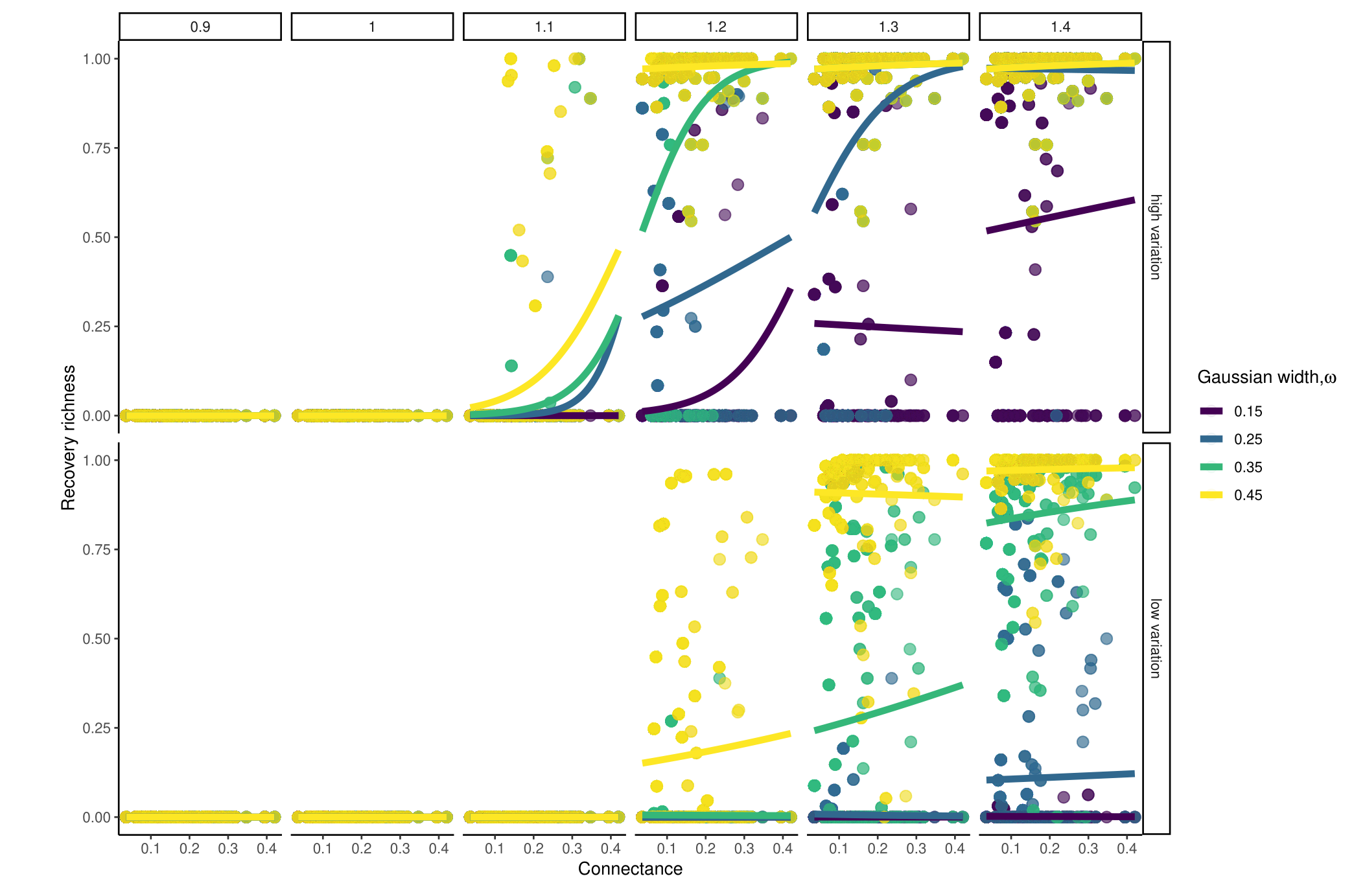

Supplement: S25 Fig — Network recovery from perturbing a single species was positively impacted by connectance shown for 2 levels of trait variation (high variation and low variation), for 4 levels of threshold mutualistic strength, γ0, of 0.9, 1, 1.1, 1.2, 1.3, 1.4, and for 4 different Gaussian function widths ω. In all these networks, only the species with the highest degree was positively perturbed from a very low density, Ni < 0.005, for a duration of 500 time points with a forcing strength of 0.5, while the rest of the species remained unperturbed. Shown here are data from 115 networks. σi was fixed at 0.02 for the high trait variation case and 0.005 for the low trait variation case, respectively. We observed that higher Gaussian kernel width, ω>0.15, leads to higher recovery. Initial mean trait values were sampled according to parameter values given in Table 1. Different colored lines representing generalized linear model fitting with quasibinomial error distributions. Underlying data and R scripts for reproducing this figure can be found in https://doi.org/10.5281/zenodo.13598906. (TIF) [file pbio.3002826.s026.tif]

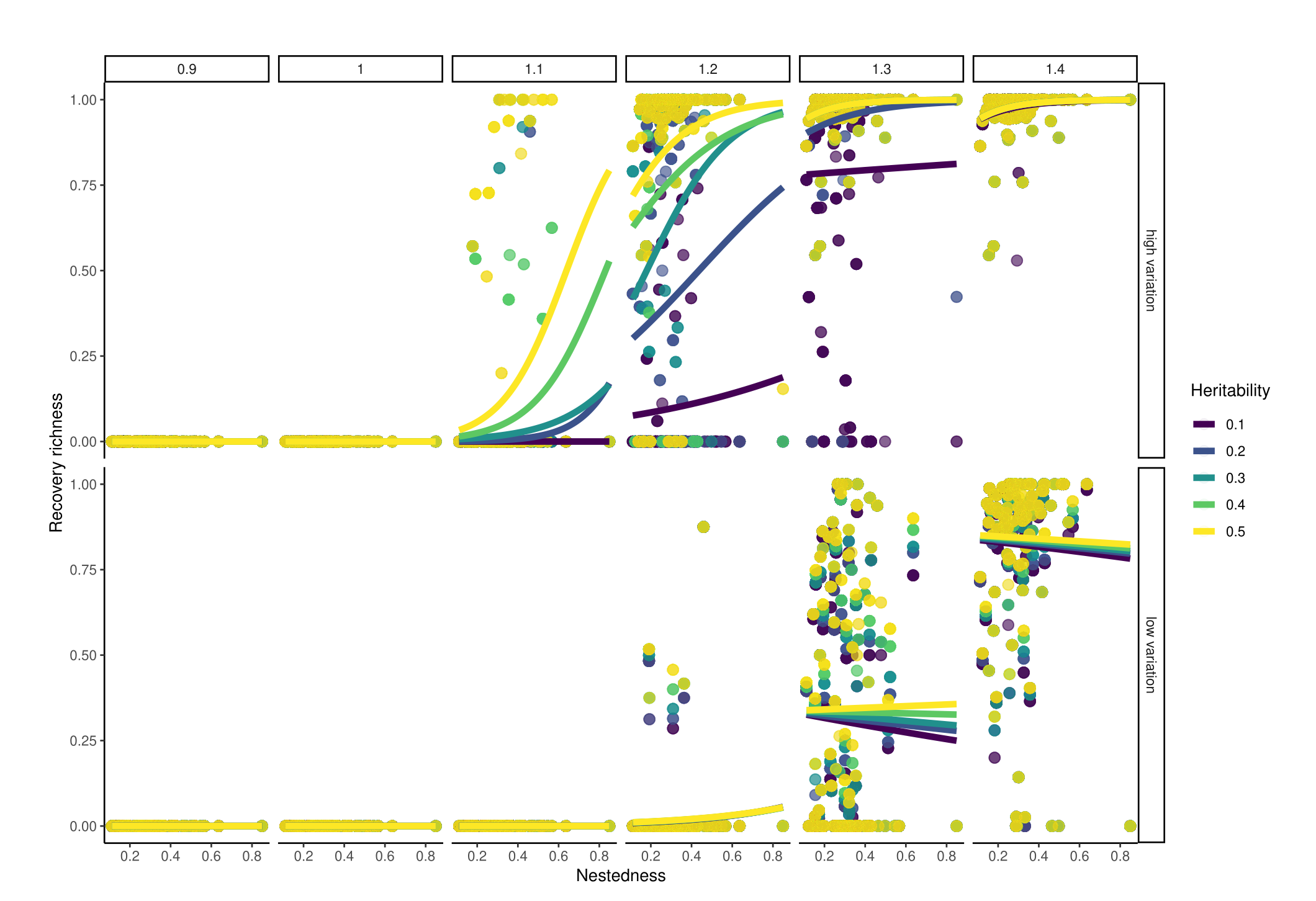

Supplement: S26 Fig — Network recovery from perturbing a single species was positively impacted by nestedness shown for 2 levels of trait variation (high variation and low variation), for 4 levels of threshold mutualistic strength, γ0, of 0.9, 1, 1.1, and 1.2, 1.3, 1.4, and for 5 different heritability values of 0.1, 0.2, 0.3, 0.4, and 0.5. In all these networks, only the species with the highest degree, was positively perturbed from a very low density, Ni < 0.005, for a duration of 500 time points with a forcing strength of 0.5, while the rest of the species remained unperturbed. Shown here are data from 115 networks. σi was fixed at 0.02 for the high trait variation case and 0.005 for the low trait variation case, respectively. We observed that higher heritability leads to better recovery. Initial mean trait values were sampled according to parameter values given in Table 1. Different colored lines representing generalized linear model fitting with quasibinomial error distributions. Underlying data and R scripts for reproducing this figure can be found in https://doi.org/10.5281/zenodo.13598906. (TIF) [file pbio.3002826.s027.tif]

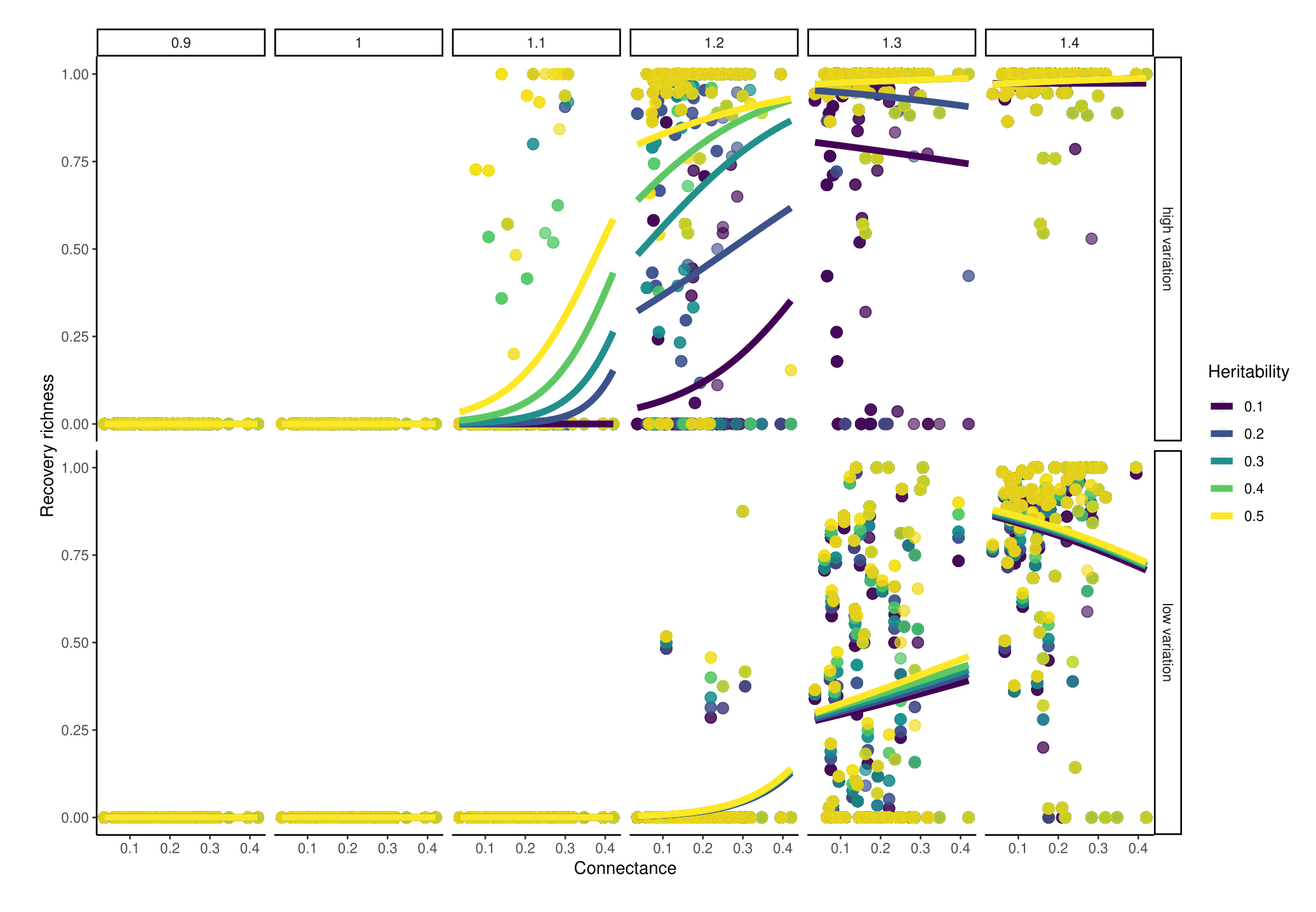

Supplement: S27 Fig — Network recovery from perturbing a single species was positively impacted by connectance shown for 2 levels of trait variation (high variation and low variation), for 4 levels of threshold mutualistic strength, γ0, of 0.9, 1, 1.1, and 1.2, 1.3, 1.4 and for 5 different heritability values of 0.1, 0.2, 0.3, 0.4, and 0.5. In all these networks, only the species with the highest degree was positively perturbed from a very low density, Ni < 0.005, for a duration of 500 time points with a forcing strength of 0.5, while the rest of the species remained unperturbed. Shown here are data from 115 networks. σi was fixed at 0.02 for the high trait variation case and 0.005 for the low trait variation case, respectively. We observed that higher heritability leads to better recovery. Initial mean trait values were sampled according to parameter values given in Table 1. Different colored lines representing generalized linear model fitting with quasibinomial error distributions. Underlying data and R scripts for reproducing this figure can be found in https://doi.org/10.5281/zenodo.13598906. (TIF) [file pbio.3002826.s028.tif]

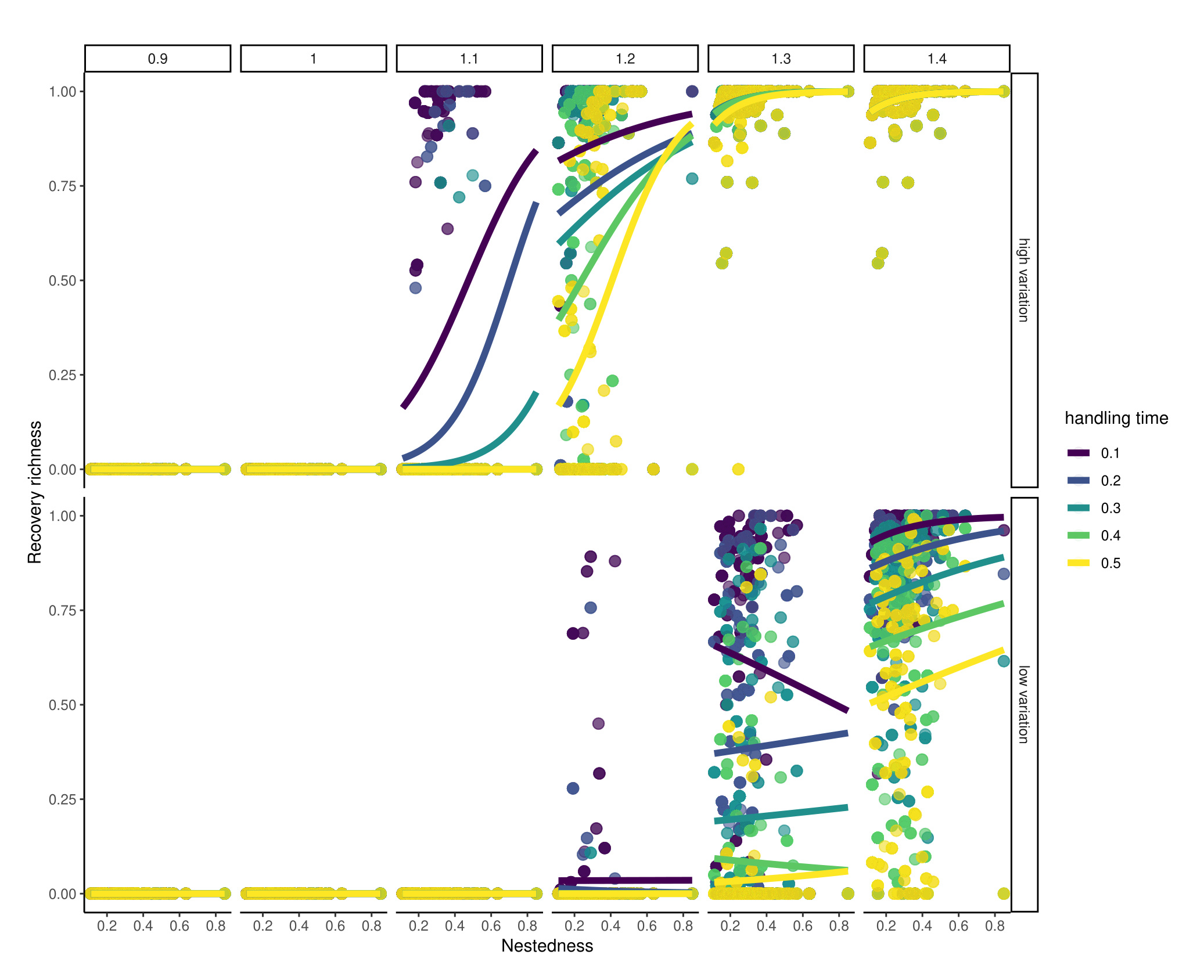

Supplement: S28 Fig — As nestedness (NODF) increased proportion of species with density greater than 0.5 increased after species-specific perturbation was stopped and more so when species had high trait variation. To start with, the networks were collated from web-of-life database and species had an initial density of Ni < 0.005, and perturbation/forcing strength of 0.5 was applied to the species with the highest degree for a duration of 500 time points. For high trait variation σi for all species was fixed at 0.02. Initial mean trait values were sampled as given in Table 1. Different colored lines representing generalized linear model fitting with quasibinomial error distributions. Underlying data and R scripts for reproducing this figure can be found in https://doi.org/10.5281/zenodo.13598906. (TIF) [file pbio.3002826.s029.tif]

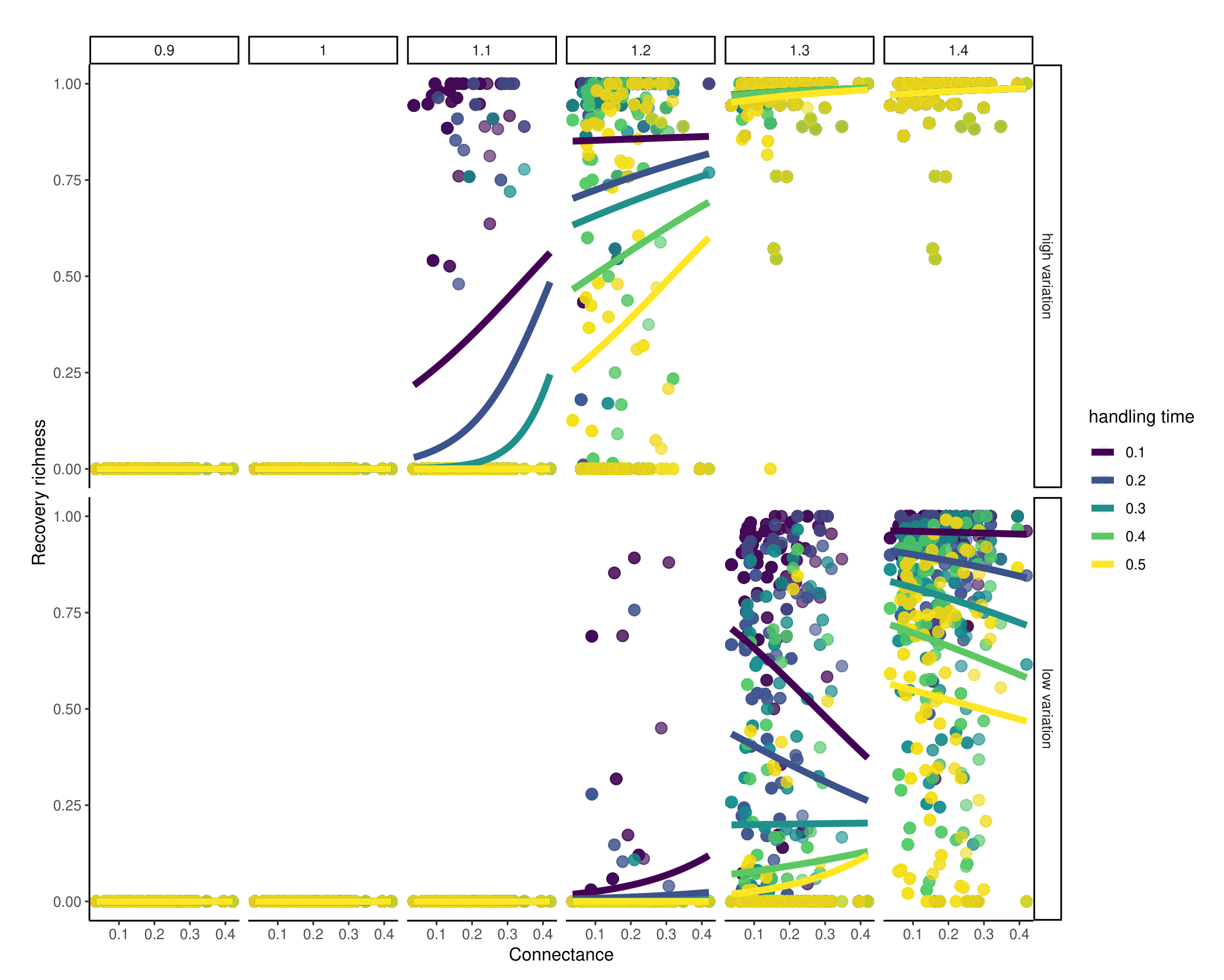

Supplement: S29 Fig — As connectance increased proportion of species with density greater than 0.5 increased after species-specific perturbation was stopped and more so when species had high trait variation. To start with, the networks were collated from web-of-life database and species had an initial density of Ni < 0.005, and perturbation/forcing strength of 0.5 was applied to the species with the highest degree for a duration of 500 time points. For high trait variation σi for all species was fixed at 0.02. Initial mean trait values were sampled as given in Table 1. Different colored lines representing generalized linear model fitting with quasibinomial error distributions. Underlying data and R scripts for reproducing this figure can be found in https://doi.org/10.5281/zenodo.13598906. (TIF) [file pbio.3002826.s030.tif]

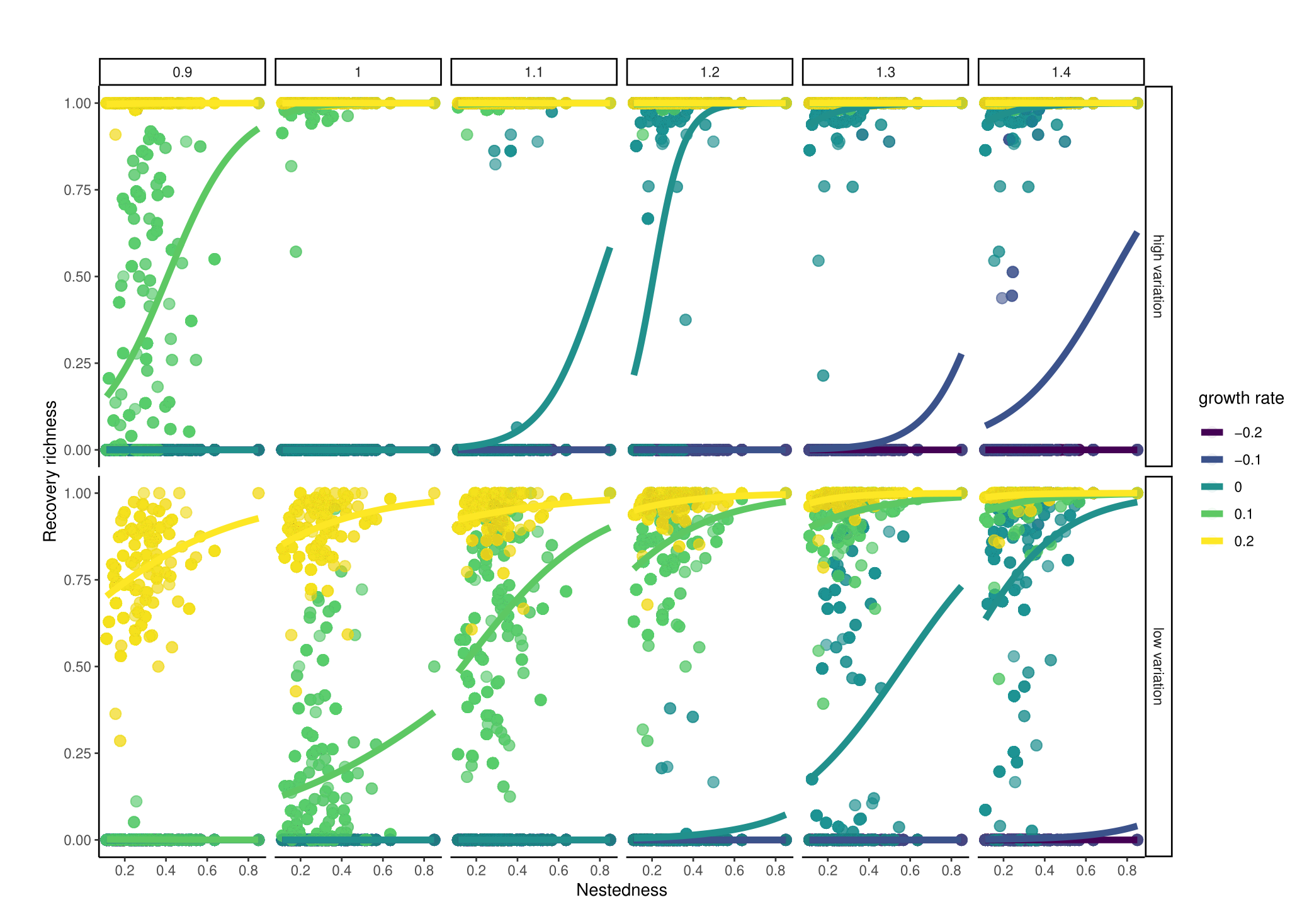

Supplement: S30 Fig — Network recovery from perturbing a single species was positively impacted by nestedness shown for 2 levels of trait variation (high variation and low variation), for 5 levels of threshold mutualistic strength, γ0, of 0.9, 1, 1.1, 1.2, 1.3, 1.4, and for 5 different growth rate values of −0.2, −0.1, 0, 0.1, and 0.2. Higher positive growth rate leads to better network recovery. Negative and zero growth rates would indicate that species are obligate mutualists. In all these networks, only the species with the highest degree was positively perturbed from a very low density, Ni < 0.005, for a duration of 500 time points with a forcing strength of 0.5, while the rest of the species remained unperturbed. Shown here are data from 115 networks. σi was fixed at 0.02 for the high trait variation case and 0.005 for the low trait variation case, respectively. Initial mean trait values were sampled according to parameter values given in Table 1. Different colored lines representing generalized linear model fitting with quasibinomial error distributions. Underlying data and R scripts for reproducing this figure can be found in https://doi.org/10.5281/zenodo.13598906. (TIF) [file pbio.3002826.s031.tif]

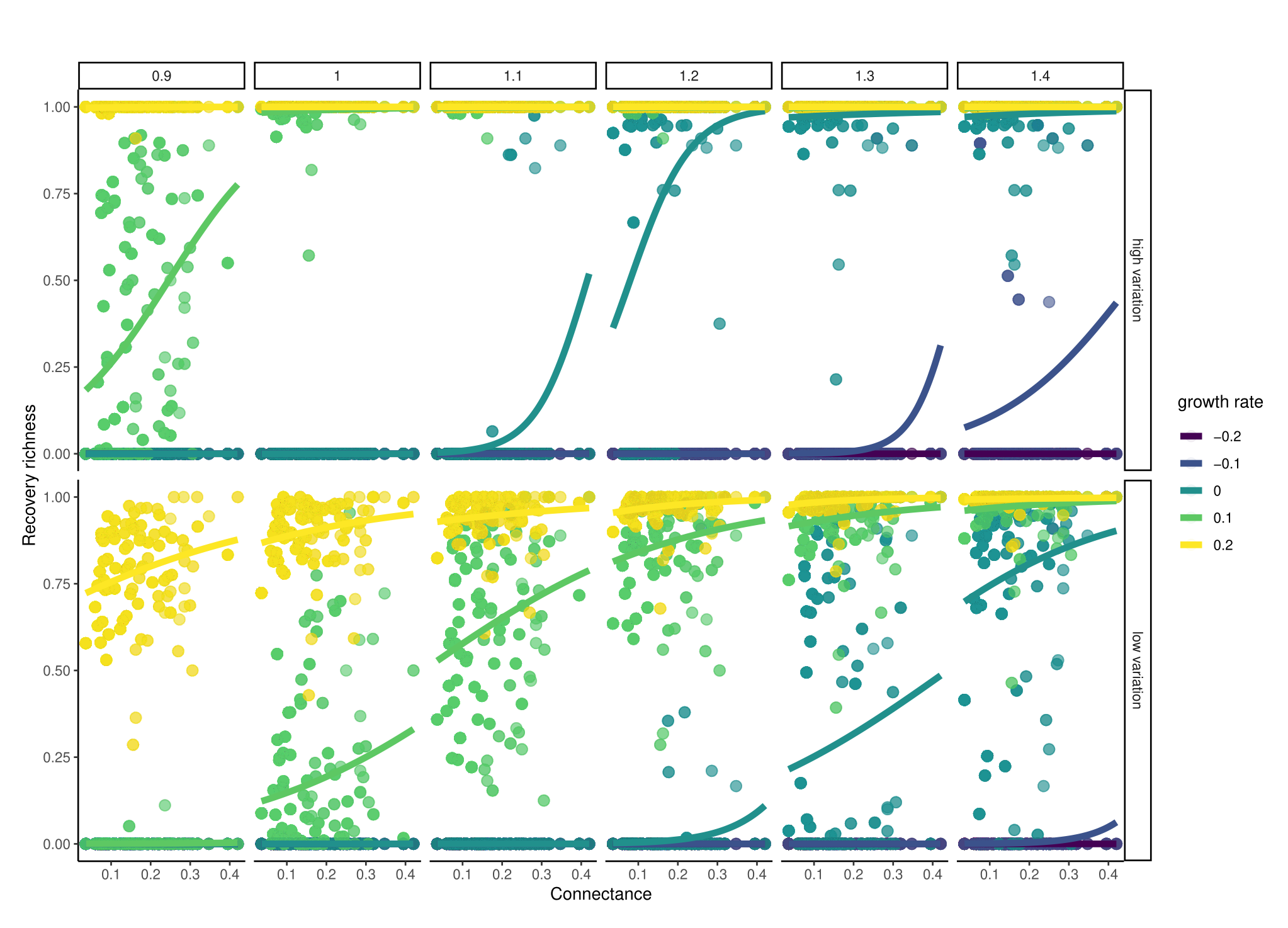

Supplement: S31 Fig — Network recovery from perturbing a single species was positively impacted by connectance shown for 2 levels of trait variation (high variation and low variation), for 5 levels of threshold mutualistic strength, γ0, of 0.9, 1, 1.1, 1.2, 1.3, 1.4, and for 5 different growth rate values of −0.2, −0.1, 0, 0.1, and 0.2. Higher positive growth rate leads to better network recovery. Negative growth rates would indicate that species are obligate mutualists. In all these networks, only the species with the highest degree was positively perturbed from a very low density, Ni < 0.005, for a duration of 500 time points with a forcing strength of 0.5, while the rest of the species remained unperturbed. Shown here are data from 115 networks. σi was fixed at 0.02 for the high trait variation case and 0.005 for the low trait variation case, respectively. Initial mean trait values were sampled according to parameter values given in Table 1. Different colored lines representing generalized linear model fitting with quasibinomial error distributions. Underlying data and R scripts for reproducing this figure can be found in https://doi.org/10.5281/zenodo.13598906. (TIF) [file pbio.3002826.s032.tif]

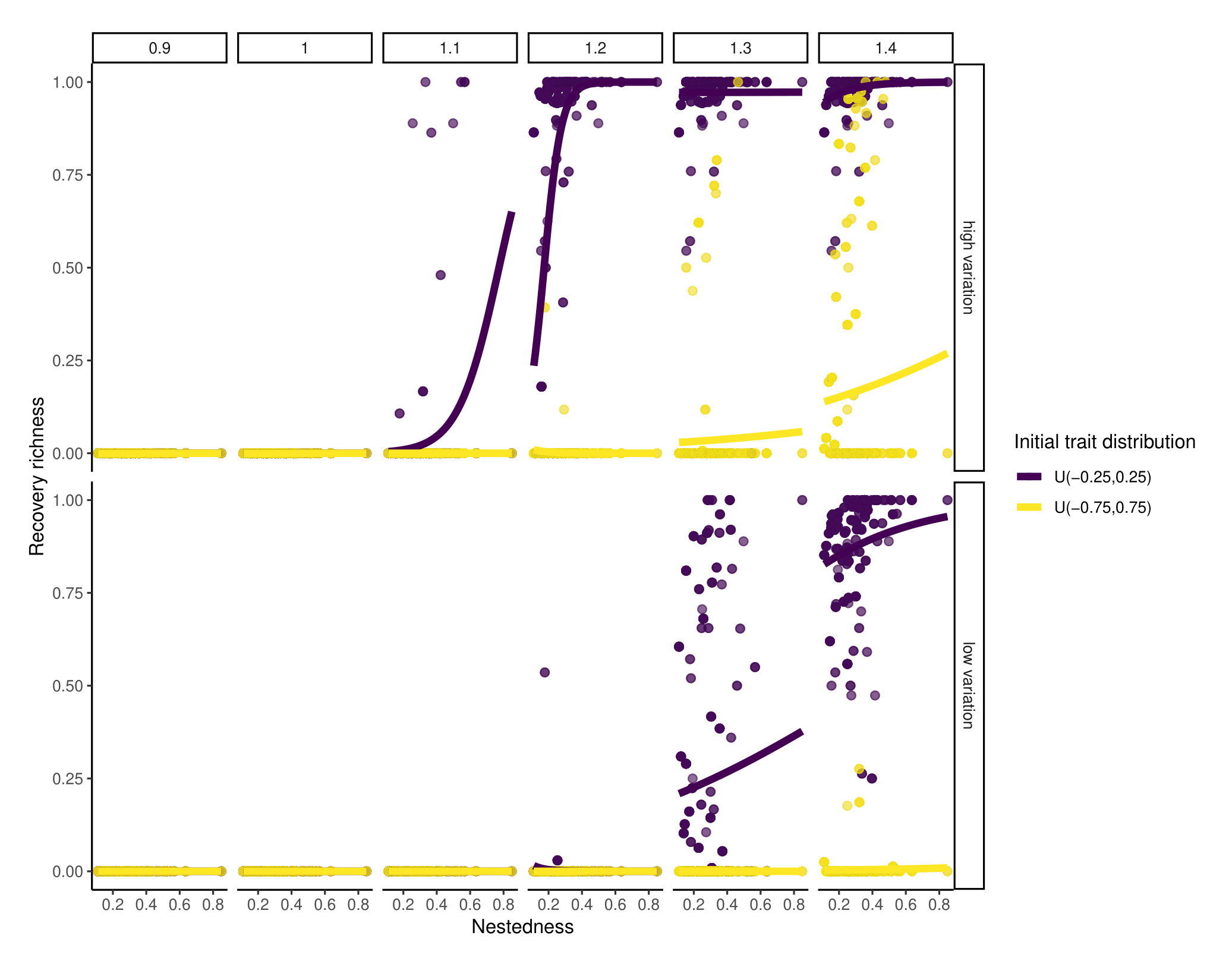

Supplement: S32 Fig — Network recovery from perturbing a single species was positively impacted by nestedness shown for 2 levels of trait variation (high variation and low variation), for different levels of threshold mutualistic strength, γ0, of 0.9, 1, 1.1, 1.2, 1.3, and 1.4 for 2 different initial trait distributions of U[–0.25, 0.25] and U[–0.75, 0.75]. Sampling of trait values from a wider trait distribution of U[–0.75, 0.75] leads to poor network recovery from collapse particularly when species have low trait variation. In all these networks, only the species with the highest degree was positively perturbed from a very low density, Ni < 0.005, for a duration of 500 time points with a forcing strength of 0.5, while the rest of the species remained unperturbed. Shown here are data from 115 networks. σi was fixed at 0.02 for the high trait variation case and 0.005 for the low trait variation case, respectively. Different colored lines representing generalized linear model fitting with quasibinomial error distributions. Underlying data and R scripts for reproducing this figure can be found in https://doi.org/10.5281/zenodo.13598906. (TIF) [file pbio.3002826.s033.tif]

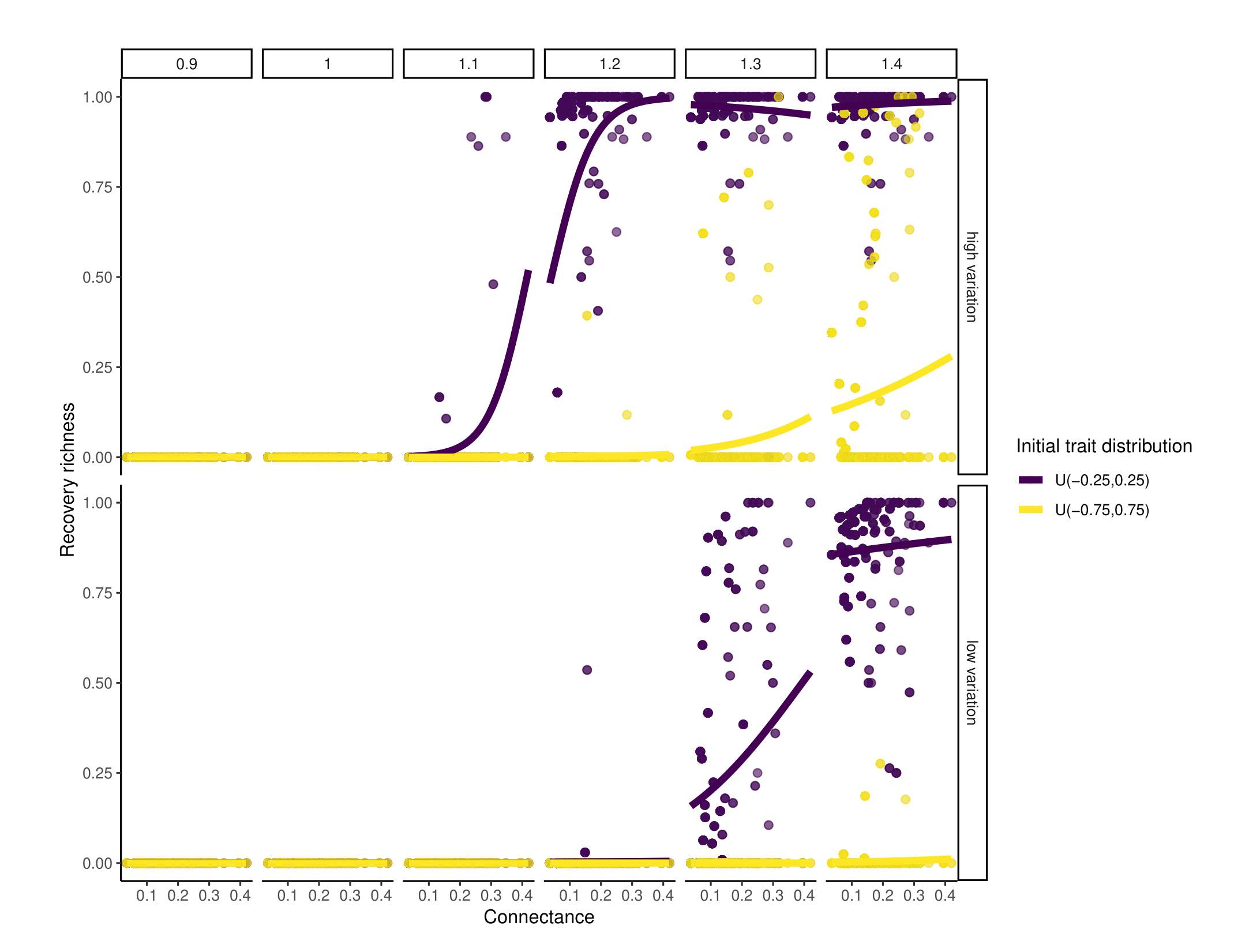

Supplement: S33 Fig — Network recovery from perturbing a single species was impacted positively impacted by connectance shown for 2 levels of trait variation (high variation and low variation), for different levels of threshold mutualistic strength, γ0, of 0.9, 1, 1.1, 1.2, 1.3, and 1.4 for 2 different initial trait distribution values of U[–0.25, 0.25] and U[–0.75, 0.75]. Sampling of trait values from a wider trait distribution of U[–0.75, 0.75] leads to poor network recovery from collapse particularly when species have low trait variation. In all these networks, only the species with the highest degree was positively perturbed from a very low density, Ni < 0.005, for a duration of 500 time points with a forcing strength of 0.5, while the rest of the species remained unperturbed. Shown here are data from 115 networks. σi was fixed at 0.02 for the high trait variation case and 0.005 for the low trait variation case, respectively. Different colored lines representing generalized linear model fitting with quasibinomial error distributions. Underlying data and R scripts for reproducing this figure can be found in https://doi.org/10.5281/zenodo.13598906. (TIF) [file pbio.3002826.s034.tif]

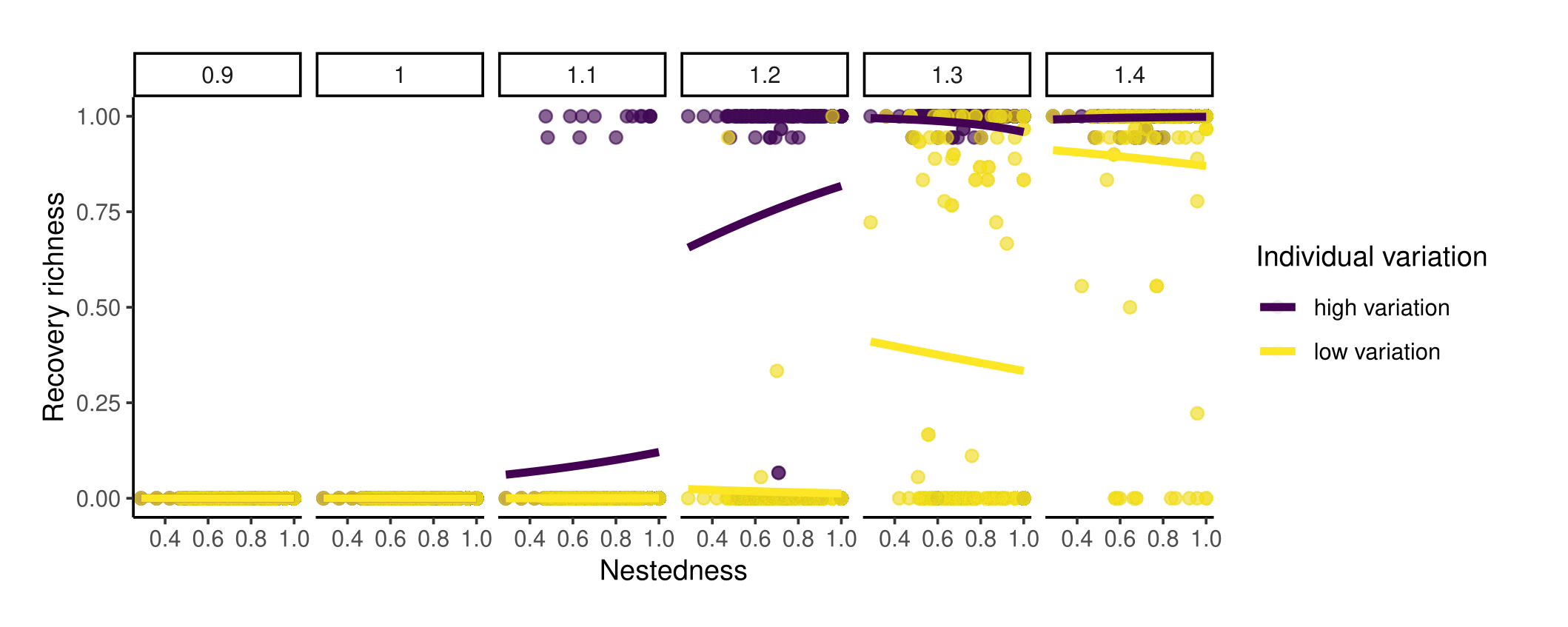

Supplement: S34 Fig — In all these networks, only the species with the highest degree was positively perturbed from a very low density, Ni < 0.005, for a duration of 500 time points with a forcing strength of 0.5, while the rest of the species remained unperturbed. Shown here are data from a range of 92 artificially generated networks that vary from a low nestedness of 0.28 to a maximum nestedness of 1 while connectance was fixed at 0.42. σi was fixed at 0.02 for the high trait variation case and 0.005 for the low trait variation case, respectively. Here, the network size was 30 species and connectance was 0.42, and then nestedness matrices keeping the connectance and network size constant were created that varied from low of 0.28 to high of 1. Different colored lines representing generalized linear model fitting with quasibinomial error distributions. Underlying data and R scripts for reproducing this figure can be found in https://doi.org/10.5281/zenodo.13598906. (TIF) [file pbio.3002826.s035.tif]

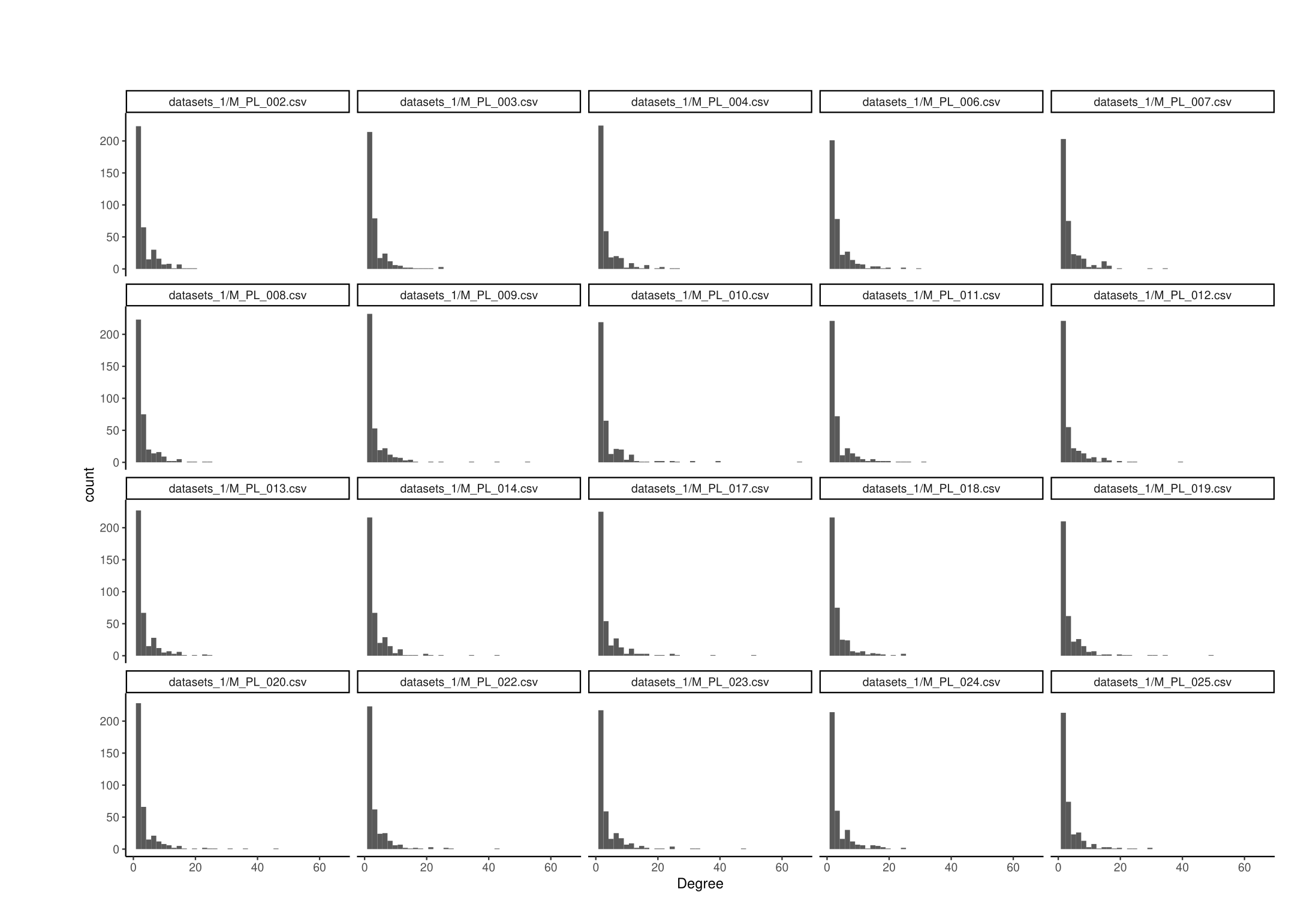

Supplement: S35 Fig — Data sets and their detailed information could be available in web-of-life database as well as attached in the GitHub repository. Underlying data and R scripts for reproducing this figure can be found in https://doi.org/10.5281/zenodo.13598906. (TIF) [file pbio.3002826.s036.tif]

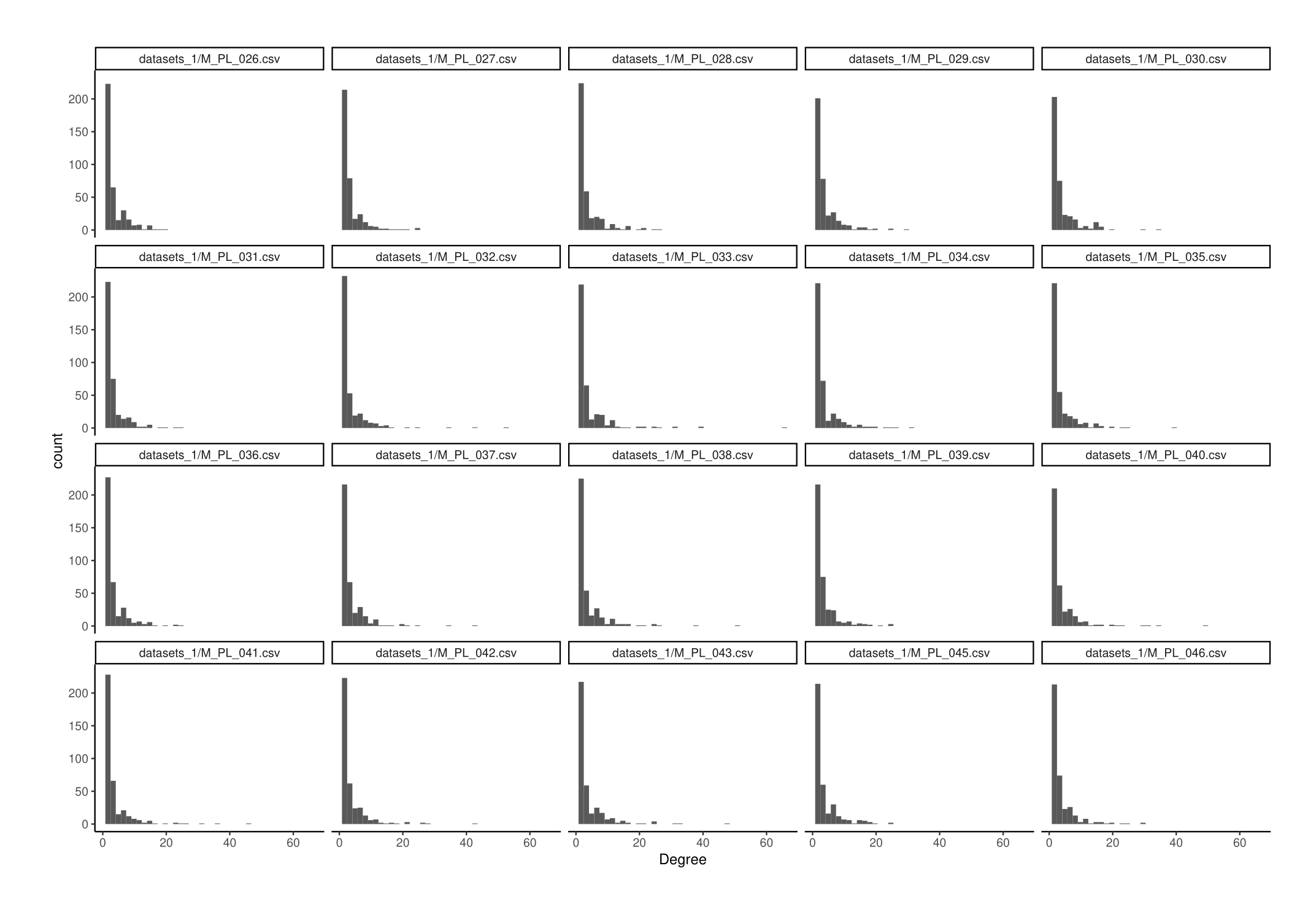

Supplement: S36 Fig — Data sets and their detailed information could be available in web-of-life database as well as attached in the Github repository. Underlying data and R scripts for reproducing this figure can be found in https://doi.org/10.5281/zenodo.13598906. (TIF) [file pbio.3002826.s037.tif]

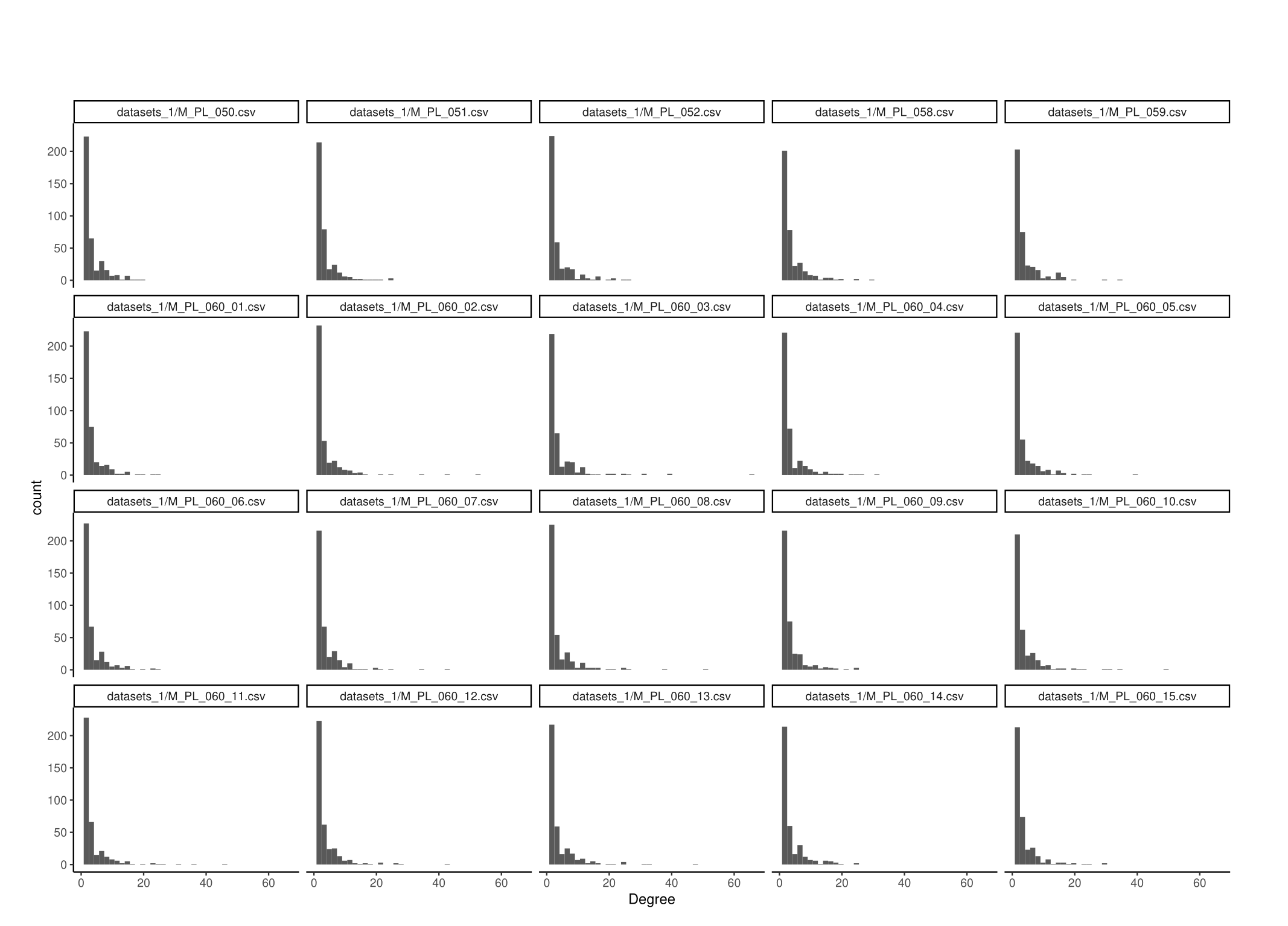

Supplement: S37 Fig — Data sets and their detailed information could be available in web-of-life database as well as attached in the Github repository. Underlying data and R scripts for reproducing this figure can be found in https://doi.org/10.5281/zenodo.13598906. (TIF) [file pbio.3002826.s038.tif]

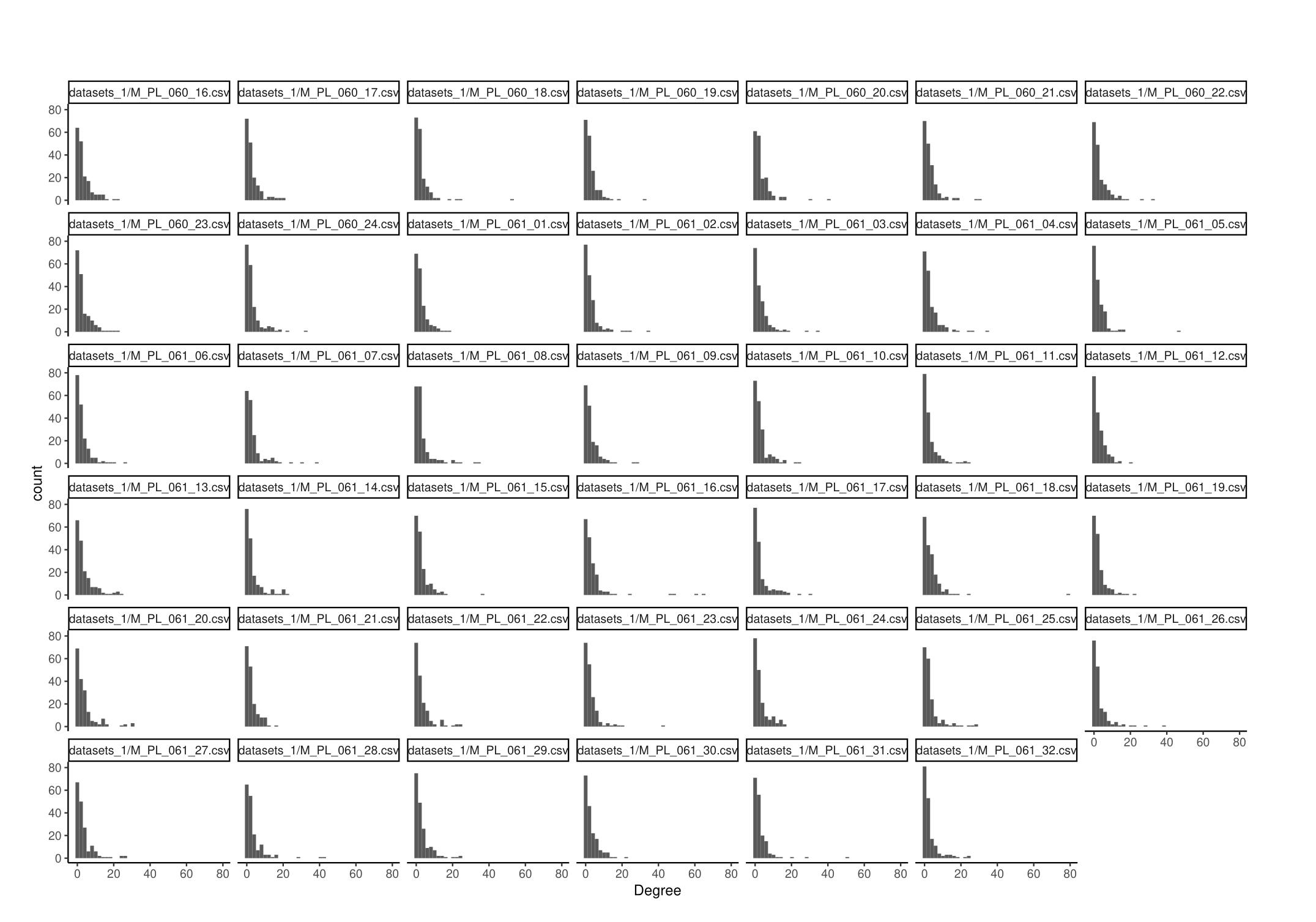

Supplement: S38 Fig — Data sets and their detailed information could be available in web-of-life database as well as attached in the Github repository. Underlying data and R scripts for reproducing this figure can be found in https://doi.org/10.5281/zenodo.13598906. (TIF) [file pbio.3002826.s039.tif]
