# Supplementary material for: Reviving collapsed plant–pollinator networks from a single species
Source: PLoS Biol. 2024 Oct 4;22(10):e3002826. doi: 10.1371/journal.pbio.3002826 (PMC11482677; doi:10.1371/journal.pbio.3002826)
Supplement: Attachment — Submitted filename: Network_resurrection_reply_letter (1).pdf [file pbio.3002826.s041.pdf]

Response to reviewers

June 25, 2024

Dear Dr. Roland Roberts and the Academic Editor,

Please find attached a revised version of the manuscript titled **"Reviving collapsed plant-pollinator networks from a single species"** for consideration for publication in **PLOS Biology**. This paper is an original, unpublished work, which has not been submitted to other journals for review.

We would like to thank the academic editor and the two reviewers for their detailed and constructive comments on the manuscript. We have carefully addressed the concerns raised by the academic editor and both reviewers. In line with the academic editor's comments, we have added additional supplementary analyses on the indirect effects on recovery biomass of networks and provided a detailed explanation of the methodology in supplementary appendix 4. Additionally, we have conducted robustness analyses on different parameters affecting our model outcomes, explored various perturbation scenarios, discussed potential limitations and assumptions of our modeling approach, and outlined future research directions based on Reviewer 1's recommendations. Following Reviewer 2's suggestions, we have included sensitivity analyses of different non-zero growth rate values, investigated the impact of nestedness by controlling for connectance and network size, and added references for heritability parameter values.

We hope you find this revised version of the manuscript acceptable for publication in **PLOS Biology**. Please find attached a point-by-point responses to the reviewer comments below, the main manuscript and a track changed document. Please refer to the clean main manuscript to match the lines numbers mentioned in the response letter.

Sincerely,

Gaurav Baruah and Meike Wittmann

Detailed responses to comments:

Editor's comments to the author(s):

1. The manuscript addresses the problem of restoration of full ecological networks from a theoretical perspective based on signal propagation theory and an eco-evolutionary model based on network structures. The authors explore the idea of hysteresis and how that could define different recovery trajectories during networks rebuilding. Hysteresis itself may work as a brake for functional recovery; this point is original and worth pursuing. Some of the ideas presented are truly innovative, and with potential general interest. Thus I'm positive to offer the possibility for a resubmission.
I've myself read the MS in detail and concur with the two referees comments. Overall the MS is well written and structured. Authors should pay attention to the reviewers' suggestions.

Reply: We thank the editor for their appreciation and their suggestions on the manuscript. We have to our best ability addressed the comments and suggestions of the reviewers as well as of the editor. Please see below for our detailed responses.

2. Theoretical-based approaches usually demand more realism, yet this also results in much greater model complexity and is often impossible to obtain. However, I think the authors could do a better leverage of their results by providing some assessment of sensitivity analysis and robustness to variations of some key aspects e.g., using a Gaussian kernel to estimate interaction strengths, binary-based nestedness estimates, or the biological bases to set initial parameter values, etc. I mean, I don't like to be over-demanding in this respect, yet a better discussion and added caution with these key parameters would be welcome.

Reply: We agree with the editor and have now addressed both the reviewer's comments by doing sensitivity and robustness analyses of our general results. We have added sensitivity analyses of all the parameters the reviewers have mentioned such as width of Gaussian interaction kernel (Fig. S24-25), changes in heritability of the mean phenotypic trait (Fig. S26-27), changes in handling time (Fig. S28-29), usage of a asymmetric species interaction kernel (Fig. S16-19), different growth rates (Fig. S30-31), usage of weighted nestedness instead of binary-based nestedness estimates (Fig. S10) etc. We have also justified the usage of certain parameter such as heritability and handling time values based on estimates observed in empirical studies. In addition, we have added different estimates of network metrics apart from just nestedness and connectance such as network modularity and mean betweenness centrality based on the recommendation of reviewer 1 (Fig. S10). Finally, we have discussed potential issues related with using such parameters on outcome of our results, as well as gaps that our model cannot address.

3. A missing issue not really addressed in the model approach is the question of indirect effects within networks and how a recovery trajectory may or may not adequately recover such indirect effects. This may be complex, as such indirect effects grow very quickly with increasing number of species and interactions. I don't mean this would require re-analysis, yet, might be a point worth deserving some discussion. For example, if indirect effects are frequent in these complex networks (any they are, certainly), then this could buffer the responses against hysteresis and somehow increase the likelihood of recovery.

Reply: We thank the editor for this insightful point, which we did not consider in our manuscript. We agree that indirect effects are pervasive in plant-pollinator networks, as previous studies by Guimarães et al. (2017) have shown that these effects constrain the coevolutionary dynamics of such networks. However, the impact of indirect effects on network recovery remains unknown. In our modeling framework, we included both competition and mutualistic interactions, so the net interactions as well as indirect interactions would be a combination of both. After careful consideration, we addressed the editor's point by quantifying the mean indirect effects faced by species in a network relative to the mean species mutualistic strength (γ_0). This analysis provides insights into the strength of mean indirect effects and their potential role in recovery (see figure attached below). We have included a supplementary analysis quantifying

these indirect effects and their impact on the recovery biomass of plant-pollinator networks (appendix 4).

The analytical approach required certain assumptions, detailed in two paragraphs below, explaining how we quantified indirect effects on recovery biomass and the associated assumptions. One of the assumptions we used was that the system was at an some kind of an equilibrium state, which was possible given the plant-pollinator systems before the start of any kind of species-specific perturbation, i.e., at $t = 0$, was at an undesirable stable state (all $N_i < 0.005$). The second assumption was that although our model follows a type-2 functional curve, but at very low species density, interactions between plant and pollinator species follow a type-1 response curve (Guimarães et al. 2011). When species-specific perturbation starts, species gain density, and the assumption of plant-pollinator interaction following a type-1 curve fails. We used this assumption of type-1 curve because otherwise the derivation of the indirect effects is not possible and would be beyond the scope of this manuscript. These assumptions then allows us to analytically quantify net and indirect interactions. Nevertheless, we can still get an insight of the impact of indirect effects that could be prevalent in such complex plant-pollinator networks.

In summary, we found that indirect interaction effects were mainly negative at low levels of γ_0, indicating a negative impact on recovery and thereby promoting hysteresis at low γ_0 values (please see the figure below). At higher value of γ_0 the derivation fails because the spectral density (the maximum of the eigenvalues) of the net interaction matrix goes beyond 1. When this happens, disentangling net interaction effects to direct and indirect interactions does not make sense (Zelnik et al. 2024). Unlike the study by Guimarães et al. (2017), our framework included competition within both species guilds, which likely led to higher net negative indirect effects, than positive effects. We have added this result to the supplementary appendix 4 and in detail in the supplementary appendix line 72-136. We also present in the results section 3.1 of the main manuscript in lines 310-313. Below is a small excerpt of the result with a figure attached (the below excerpt is detailed in the supplementary appendix section 4):

> "Having now the information of the coefficients of $\mathbf{B}$, we can finally derive the net indirect effects. For this, the first assumption is steady state of the system. The steady state can be written as: $(\boldsymbol{I} - \mathbf{B})^{-1}$ is the matrix of net interactions that contains all interactions ranging from direct to indirect interactions. Now $(\boldsymbol{I} - \mathbf{B})^{-1}$ is a Neumann's series (Guimarães et al. 2017, Zelnik et al. 2024, Sasane 2017).

$$(\boldsymbol{I} - \mathbf{B})^{-1} = \boldsymbol{I} + \mathbf{B} + \mathbf{B}^2 + \mathbf{B}^3 + ... \tag{13}$$

where, $\mathbf{B}$ is the matrix of direct indirections and $\mathbf{B}^2$ is a matrix of second-order indirect interactions, $\mathbf{B}^3$ is the matrix of third-order indirect interactions. From this, equation 13, can be further separated to give us the net indirect interactions, which is:

$$(\boldsymbol{I} - \mathbf{B})^{-1} - (\boldsymbol{I} + \mathbf{B}) = \mathbf{B}^2 + \mathbf{B}^3 + ... \tag{14}$$

$$\mathbf{B}^2(\boldsymbol{I} - \mathbf{B})^{-1} = \mathbf{B}^2 + \mathbf{B}^3 + ... \tag{15}$$

Thus, L.H.S of equation 14 thus captures all the indirect interactions present in a system that follows equation 6 and is at a equilibria.

With this, we further calculated mean indirect effects faced by a species j i.e., B_j, per network as:

$$\beta = \frac{\sum_{i,j}^{S} B_{ij}}{S} = \frac{\sum_{i,j}^{S} B_{ij}^2 (1 - B_{ij})^{-1}}{S} \tag{16}$$

Finally, we further quantified mean indirect effects of order 3 and order 4 i.e., $\mathbf{B}^3$ and $\mathbf{B}^4$ as β^3 and β^4 respectively. Finally, quantifying indirect effects will only make sense if the spectral radius, i.e., the absolute maximum of the eigenvalue of the matrix $\mathbf{B}$ is less than 1 Zelnik et al. (2024). If spectral radius becomes greater than 1, then net interactions is not equal to the sum of direct and indirect interactions or equation 13 does not converge. In that sense, estimating β will not make sense.

In Fig. S14 and S15, we observe that indirect interaction effects are mostly negative, indicating that at low γ_0 values, indirect interactions promotes hysteresis. Indirect effects being negative is indicative of competitive effects that we modelled within each guild of species. At higher values of $\gamma_0 > 1$, we observe that spectral radius, i.e., the absolute maximum of the eigenvalue of the matrix $\mathbf{B}$ becomes greater than 1, indicating that our linearity assumption with type-1 functional curve might have failed, as at higher γ_0 values species densities slightly increase, which might have led

to spectral radius being greater than 1. Note that Guimarães et al. (2017) did not have competitive interactions modeled in contrast to our study.”

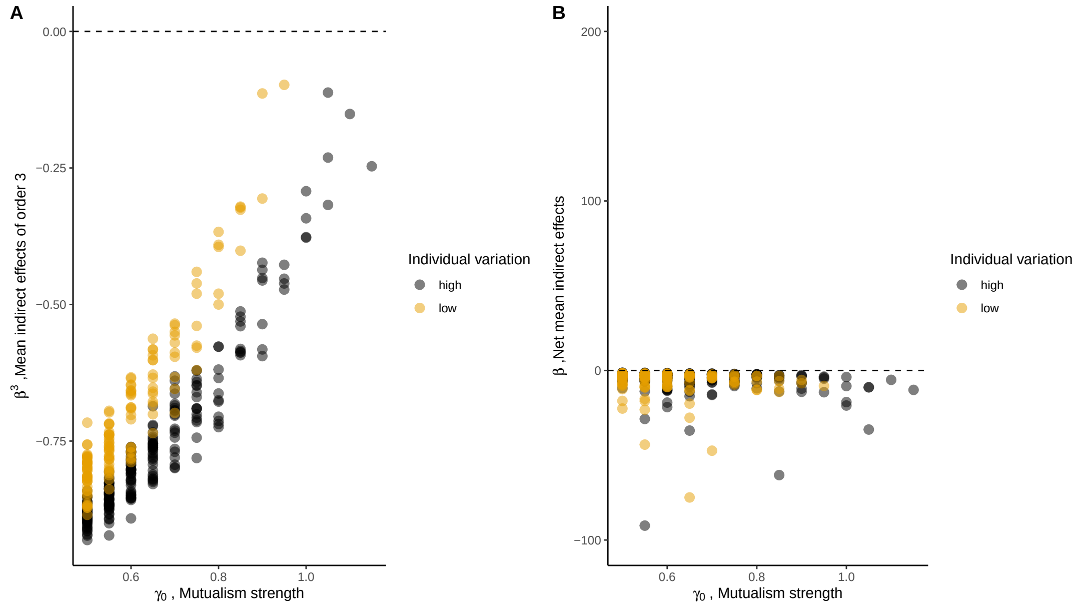

Figure S15: Mean indirect effects faced by a species in a network in relation to mean threshold interaction strength γ_0. (A) Mean indirect effects of order 3, β^3. At low γ_0, mean indirect effects are mostly negative and remains below 0, but became less negative as γ_0 increased indicating a positive impact of higher γ_0. (B) Similarly, net mean indirect effects of all orders, β, in relation to γ_0. Net indirect effects are mostly below zero. To be noted that indirect effects shown here fulfills the condition of equation 11 in supplementary appendix, and it happens when spectral radius of **B** are less than 1.

4. The authors use a dynamical eco-evolutionary framework, to revive mutualistic networks from an undesirable alternative stable state to a high-functioning stable state at unfavorable environmental conditions. The starting point is a single species scenario that may look too simplistic from an empirical, biologically realistic perspective. The idea of setting the focus on a few species whose dynamics could steer the entire network to resurrection is interesting, yet I miss further explanation of how such a distinct subset of species might be a priori identified.

Reply: We thank the editor for pointing this out. We agree that focusing on a single species to revive collapsed networks could be overly simplistic. The aim of our manuscript was to demonstrate that a detailed survey of demographic information may not be necessary for reviving

a collapsed plant-pollinator network. Using just binary interaction data or frequency interaction data, one can make a preliminary judgment by selecting the species with the highest degree as the focal point for network revival. We have now explained this in greater detail. Furthermore, we investigated the potential benefits of selecting a subset of species for network revival. We conducted additional analyses measuring the betweenness centrality of species within the network and chose three species with the highest betweenness centrality for a "targeted perturbation" scenario, as suggested by the editor. These results are discussed in our manuscript, highlighting the potential of using a subset of species to steer the dynamics of a collapsed network towards a high-functioning state. We now discussed this on lines 472-499 :

> “Our study suggests that, without detailed demographic data, increasing the survival of a super generalist species might be the simplest approach to recovering a collapsed network. However, implementing a strong perturbation on a generalist species poses challenges. If the generalist is a pollinator, it may not be effective in pollen transfer, potentially hindering recovery (Menz et al. 2011). Additionally, simply increasing generalist pollinator abundance may not suffice, as they need more than floral resources to thrive (Harmon-Threatt 2020, Roulston and Goodell 2011, Brosi and Briggs 2013). Conversely, increasing the density of generalist plants could be key, as they support diverse pollinator populations and aid the persistence of rare plants (Harmon-Threatt and Hendrix 2015, Wilkerson et al. 2014). Focusing on a single species is less resource-intensive than targeting multiple species, which involves higher financial and ecological costs. However, targeting multiple species increases the likelihood of successful revival. If a targeted perturbation of one species fails due to unforeseen circumstances, other species may still support the recovery process. Therefore, one effective strategy may be a targeted perturbation, selecting species based on their roles in the community, such as their centrality. In our study, targeted perturbation of the three species with the highest centrality in the plant-pollinator network led to faster recovery than the baseline approach of choosing a single species with the most interactions (Fig. S20-S23). Out of the 115 networks analyzed, 106 had plants as the most generalist species. Plants, being immobile, are easier to handle for targeted perturbation compared to pollinators. Therefore, pollinators are not ideal for targeted perturbation. Instead, focusing on increasing the density of generalist

plants could be a more effective strategy for recovering collapsed networks (Menz et al. 2011, Devoto et al. 2012) in addition to restoration of habitats (Kaiser-Bunbury et al. 2017, Williams 2011). As shown in empirical studies, increasing the density of one or more generalist plant (Harmon-Threatt and Hendrix 2015, Wilkerson et al. 2014), or choosing plant species based on high centrality values could be key, as they support diverse pollinator populations and could aid the persistence of rare plants (Wilkerson et al. 2014). In comparison to our modelling study, previous studies have indicated that introducing species that could be generalists has positive impact on the structure of plant-pollinator communities (Russo et al. 2014, Bjerknes et al. 2007), and further could help in restoring these communities (Devoto et al. 2012)."

5. Minor NOTES:

1. In Fig 1, panel A will look too much dense. Try to set thinner line thickness and/or increase a transparency alpha value for the line color. See for instance the adequate line thickness in Fig 3, panels I and L. Also well set in Fig S8 of Suppl Mat.

Reply: We have now corrected this and decreased the line thickness.

Referees' comments to the author(s):

Referee 1

Summary: The authors investigate how to restore mutualistic ecological networks that have been disrupted by environmental changes. They apply a dynamical eco-evolutionary model informed by empirical plant-pollinator networks and signal propagation theory to examine how network structure, trait variation, and species-specific perturbation influence network recovery. They show that reverting to the original environmental conditions is insufficient for network recovery, but that manipulating a single generalist species can revive the network even in adverse conditions. They also show that network nestedness and trait variation enhance network revival. They suggest that their findings have implications for the conservation and restoration of mutualistic networks.

Review: The authors address an important and timely question in ecology, namely the resilience and restoration of mutualistic networks. They use a novel combination of theoretical and empirical methods to explore how to restore collapsed networks from a single species. The manuscript is well-written, clear, and concise. The methods are well-explained and the results are supported by figures and tables. The discussion is insightful and connects the findings to the existing literature. The manuscript makes a significant contribution to the field and has the potential to stimulate further research on network dynamics and restoration. However, I have some comments that I think the authors should address before the manuscript can be accepted for publication.

Reply: We thank the reviewer for their comments and suggestions on the manuscript. We have addressed their comments to the best of our ability in the current version of the manuscript.

(1) The authors introduce a new model for the problem of interest, but they do not provide any analytical discussion or justification for their approach. They simply set all the parameters according to Table 1 and run numerical simulations. However, they do not explain how they selected the parameter values, nor how they assessed the quality of the simulations. Therefore, the robustness and validity of their results are questionable. The authors should either derive some theoretical analysis or approximation for their model, or conduct more comprehensive and rigorous numerical simulations to demonstrate its performance and limitations under different conditions and assumptions.

Reply: In this study, we derived a modified version of the generalized Lotka-Volterra equations incorporating a type-2 functional response, which includes both mean trait dynamics and population dynamics. This model builds on previous studies. We acknowledge the need to provide detailed information about the parameter values used in our study. We have now included this information, along with justifications for the parameters used such as heritability values, and handling time values. We also do a robustness check with different parameter values on the outcome of our general results. Please refer to lines 183-188:

> "In plant-pollinator empirical studies, heritability of morphological traits of pollinators that are involved in pollination such as the proboscis was as high as 0.75 with

other morphological traits ranged from 0.1 to 0.9 (Koffler et al. 2017). For plant floral morphology, heritability was also very high with a median value of 0.55, which thus was around the value we used in our modelling framework (Romero-Bravo and Castellanos, Kaczorowski et al. 2008)."

And after lines 153 and equation 3 in the main-text:

"H is the handling time and $H > 0$, which means increases in growth rate due to mutualistic plant-pollinator interaction follows a type 2 functional response curve. We have fixed H at 0.25, which is within the range that was observed in plant-pollinator interactions (Klumpers et al. 2019) (but we do a robustness check for different handling time values in the appendix Fig. S28-29)."

In addition, we also have now done rigorous numerical simulations to demonstrate that our overall result of perturbing one single species to recover collapsed network remains fairly robust despite variation in different parameter values as detailed in the next responses.

(2) The authors do not perform any sensitivity analysis or robustness checks on their model results. They only vary one parameter at a time (the average mutualistic strength, the forcing strength, or the duration of perturbation) and report the outcomes. However, it is possible that the model behavior and the network revival depend on the interactions and combinations of multiple parameters, as well as on the initial conditions and the randomness of the model. It would be useful to explore how the model results change under different scenarios and parameter values, and to test the effects of parameter uncertainty and variability on the model predictions.

Reply: We thank the reviewer for their comment and suggestion. We acknowledge that in the previous version of the manuscript we did not examine the model behavior in detail. However, a prior study by Baruah (2022) conducted a robustness check of similar model behavior under different parameter combinations, specifically different competition strengths. We have now conducted a robustness check of parameter uncertainty on the overall results regarding collapsed network recovery. This check includes variations in parameters such as heritability of the mean phenotypic trait, initial mean species trait distribution, handling time, the width of the

species interaction kernel, type of species interaction kernel etc. Overall, the main result—that a network can be revived from a single species—remains robust. However, recovery is easier with some parameter values and more challenging with others. For example, when heritability is very high ($h^2 = 0.5$), network recovery is faster compared to when heritability is low ($h^2 = 0.1$) (see the figure below and additional figures in the appendix). We further detail the results in the supplementary appendix Fig. S28-29 for handling time, Fig. S24-25 for Gaussian kernel width, Fig. S16-19 asymmetric interaction kernel, Fig. S26-27 for heritability, Fig. S32-33 for initial trait distribution. We discuss these results in the result section now in lines 381-387.

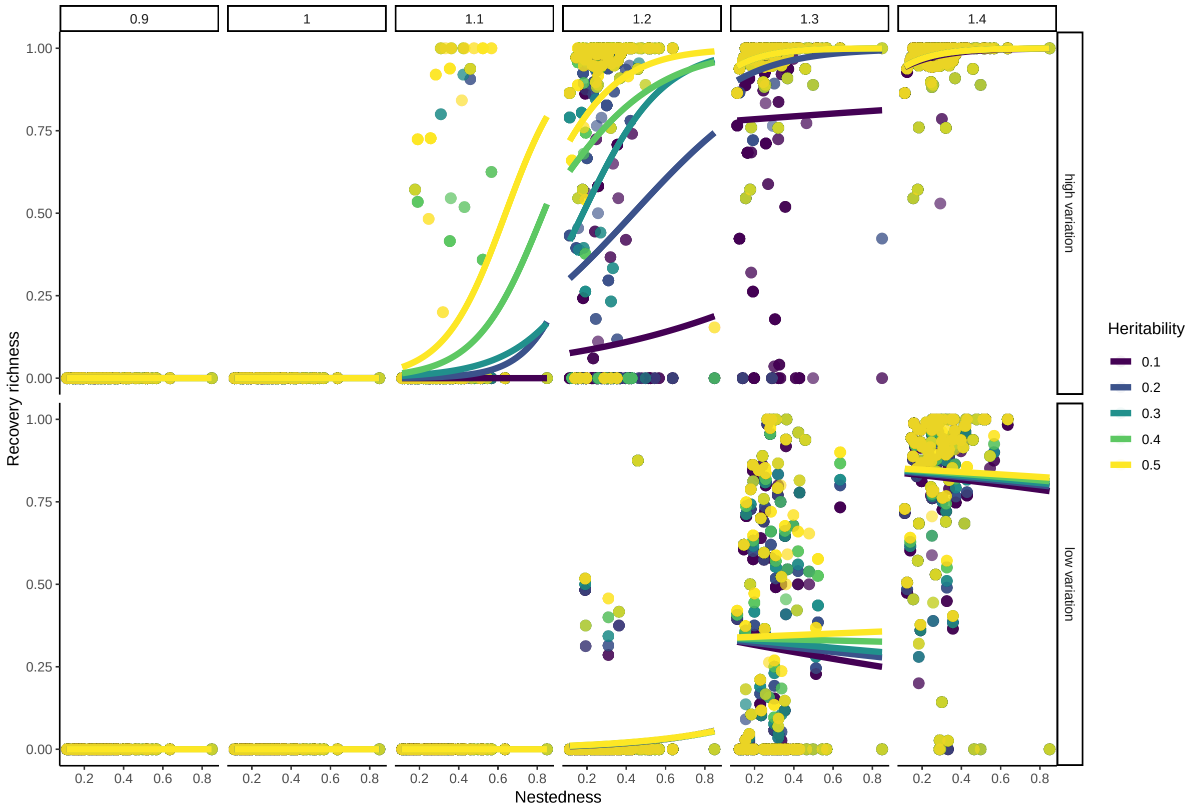

Figure S26: Impact of variation in heritability on network recovery from perturbing a single species. Network recovery from perturbing a single species was positively impacted by nestedness (NODF) shown for two levels of trait variation (high variation, low variation), for four levels of threshold mutualistic strength, γ_0, of 0.9, 1, 1.1, and 1.2, 1.3, 1.4, and for five different heritability values of 0.1, 0.2, 0.3, 0.4, 0.5. In all these networks, only the species with the highest degree, was positively perturbed from a very low density, $N_i < 0.005$, for a duration of 500 time points with a forcing strength of 0.5, while the rest of the species remained unperturbed. Shown here are data from 115 networks. σ_i was fixed at 0.02 for the high trait variation case and 0.005 for the low trait variation case respectively. We observed that higher heritability leads to better recovery. Initial mean trait values were sampled according to parameter values given in table 1. Different colored lines representing generalised linear model fitting with quasibinomial error distributions.

(3) The authors adopt a phenomenological model of mutualistic interactions that assumes a Gaussian interaction kernel and a type-2 functional response. While

this model has been widely used in previous studies, it has some limitations and assumptions that may affect the generality and applicability of the results. For instance, the model does not account for the effects of spatial structure, dispersal, density-dependence, or frequency-dependence on network dynamics and stability. Furthermore, the model assumes that the phenotypic traits of the species are continuous and normally distributed, which may not be realistic for some plant-pollinator systems. The authors should discuss these limitations and assumptions in more detail and justify their choice of model. They should also investigate the robustness and sensitivity of their results to different model parameters and assumptions, such as the shape and width of the interaction kernel, the handling time, the heritability, and the initial trait distribution.

Reply: We thank the reviewer for raising this point. We will address it in four separate points:

1. We agree with the reviewer that our model is a modified version of the phenomenological Lotka-Volterra model with a type-2 functional response. We also acknowledge that our model does not incorporate any spatial structure or species dispersal within such a formulation. While it is possible to include these aspects, doing so would require additional modeling efforts that are beyond the scope of this manuscript. We recognize this limitation and discuss it in the discussion section (see lines 527-535).

> "Our modeling framework did not account for species dispersal or spatial structure, both of which can significantly influence revival strategies for collapsed plant-pollinator networks. Dispersal is known to rescue declining populations, enhance productivity, and provide spatial insurance in fragmented landscapes (Loreau et al. 2003, Thompson and Gonzalez 2017). Incorporating dispersal into our model could potentially lead to faster recovery. Additionally, the spatial arrangement of habitat patches could impact recovery, as the structure of habitat patches influences the metacommunity dynamics and processes (Thompson and Gonzalez 2017). One important avenue for future research would be to evaluate how spatial arrangement of habitat patches could interacts with restoration strategies. "

2. Additionally, we do have density dependence through intraspecific competition, which was fixed at 1 for all species, ensuring that intraspecific competition exceeded interspecific competition. This was done to ensure that interspecific competition did not play a major role in community dynamics where mutualistic interactions are dominant. Furthermore, we have now included references for the competition values used, which qualitatively reflect those observed in empirical studies. Now written in main-text in lines 177-182. However, we have to admit that we were unsure what the reviewer meant by frequency-dependence in our model.

> "This specific distribution indicates that species competition was not too strong to dictate dynamics in a mutualistic community. These distributions were particularly used such that local dynamics became feasible and stable (Barabás et al. 2017, Baruah 2022). In addition, the strength of intraspecific competition was generally stronger than interspecific competition strengths, which were within the distribution empirically measured in plant-pollinator communities (Johnson et al. 2022) or in plant communities only (Wiegand et al. 2021)."

3. Thirdly, we acknowledge the reviewer's point that our model, based on quantitative genetics, assumes normally distributed phenotypic traits, which may not be entirely realistic. Addressing this issue would require a detailed individual-based model that does not specify trait distributions or another dynamical trait variable that is the variance in such distribution that would be under selection through mutualistic interaction and perturbation, which is beyond the scope of this study. However, we have discussed this limitation in the discussion section on lines now 536-546:

> "We also assumed that trait distributions remain normal, based on our quantitative genetics framework where variance stays constant despite selection on mean traits. In reality, trait distributions could deviate from normality. Relaxing this assumption would require a computationally intensive individual-based approach. Furthermore, we assumed that trait variance is fixed and does not evolve (Barabás et al. 2022). This assumption may not hold under strong selective pressures to match partner traits. We used low trait variance to mimic low population density conditions. However, if phenotypic variance evolves under strong species-focused perturbation, selection to match multiple partners' traits could increase variance, potentially aiding in faster recovery. Once the network recovers and species traits align, stabilizing

selection on the mean could reduce variance. This hypothesis requires further detailed evaluation, particularly on how species' trait variance evolves in response to targeted or species-focused perturbation."

4. Finally, in accordance with the reviewer's suggestion, we have now investigated our sensitivity of our modelling results by varying the width of the interaction kernel, and also trying a different species interaction kernel that is asymmetric in nature by using a Gamma distribution. Now written in the main-text lines 157-169 and also in appendix 3 of the supplementary document. We detail this in the supplementary and discuss it in our results section (lines 364-365) (also see Fig. S16, S17, S18, S19 in the appendix 3). Also attached below is one example Fig. S17 of a comparison between Gaussian interaction kernel and asymmetric interaction kernel.

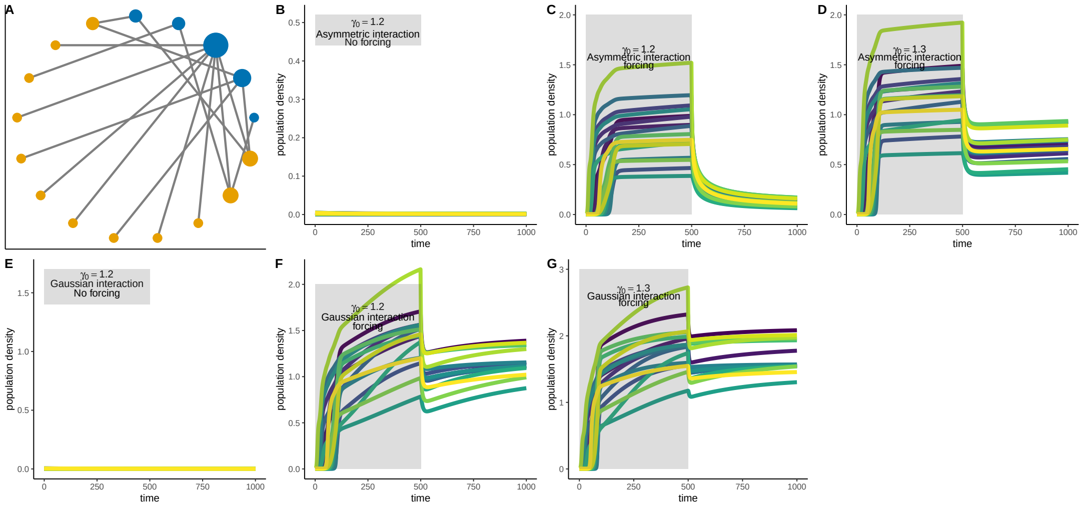

Figure S17: Network resurrection from perturbing a single species in a 17 species plant-pollinator network where species interaction was either mediated by Gaussian interaction function as in main-text or by an assymetric interaction kernel (see figure S16) . B) When there is no species specific forcing, at $\gamma_0 = 1.2$, the network remains at the undesirable state. C) At forcing strength of 0.5 for the species with the highest degree, and with species interaction dictated by a assymetric kernel, we do not observe network recovery after the perturbation was stopped at 500 time points. D) However, at $\gamma_0 = 1.3$, we do see a network recovery, but the overall biomass achieved was low. E) Same network, with Gaussian kernel, without forcing, the network remains at the undesirable state. F) However, at $\gamma_0 = 1.2$, and species specific forcing for a duration of 500 time points, we see the network recover, while achieving slightly higher biomass than the case of asymmetric species interaction. G) At higher γ_0 if 1.3, the same network with Gaussian interaction kernel readily recovers with an overall higher biomass on average. In all these simulations, w e considered initial starting species densities to be $N_i < 0.005$ and trait variation for all species to be at a moderate level of $\sigma_i = 0.02$.

(4) The authors use a measure of network nestedness (NODF) that is based on the binary adjacency matrix of the network. However, this measure does not

account for the variation in interaction strength or frequency among the species, which may also affect the network dynamics and resilience. The authors should consider using a weighted measure of network nestedness that incorporates the interaction strength or frequency. In addition, they should also report and analyze the values of other relevant network metrics, such as modularity, degree distribution, and centrality.

Reply: We thank the reviewer for highlighting this issue. We acknowledge that our initial analysis used binary-based nestedness measures. In response to the reviewer's suggestion, we have now performed additional analyses using weighted measures of network nestedness. Additionally, we have measured other relevant network metrics such as modularity and betweenness centrality. Below, we provide a figure illustrating these metrics, including weighted nestedness, betweenness centrality, and modularity (Fig. S10). We have also reported the degree distribution values of the analyzed networks in appendix figure S35-S38. These additions have been discussed in the methods section on lines 290-297 and added further in the supplementary appendix section 2.

> "In asking this question, we also, in an additional supplementary analyses, tried to investigate the role of indirect effects in recovery biomass of plant-pollinator networks (see appendix 4 for details). As number of species in a plant-pollinator network increases indirect effects will increase (Guimarães et al. 2017, Zelnik et al. 2024). Finally, we also tested the impacts of network recovery richness with other network metrics such as modularity (Newman 2006), mean betweenness centrality and weighted nestedness (Almeida-Neto et al. 2008) (see appendix 2). We also report the degree distribution of the plant-pollinator networks in the appendix Fig. S35-S38."

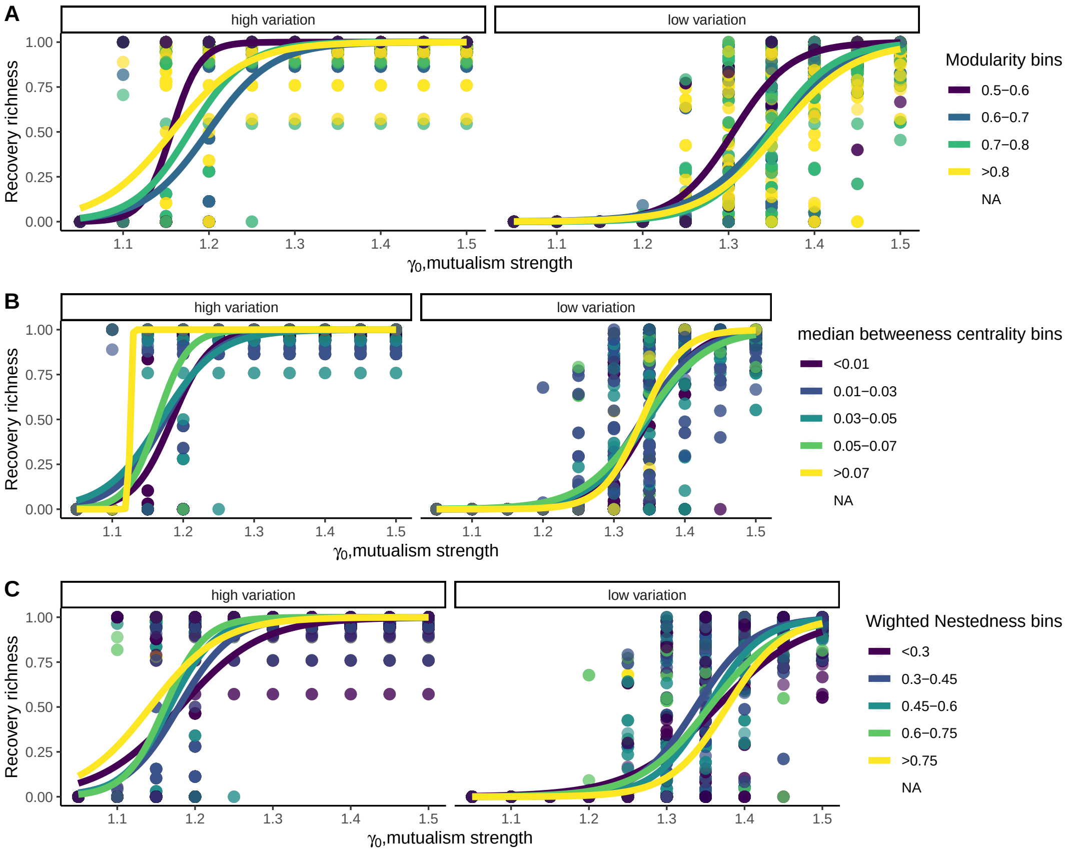

Figure S10: Network recovery from perturbing a single species was impacted moderately by network modularity (A), positively impacted by mean betweenness centrality in a network (B), and positively by weighted nestedness (C), particularly when species had higher trait variation. In each of these networks, only the species with the highest degree, was positively perturbed from a very low density, $N_i < 0.005$, for a duration of 500 time points with a forcing strength of 0.5, while the rest of the species remained unperturbed. Shown here are data from 115 networks. σ_i was fixed at 0.02 for the high trait variation case and 0.005 for the low trait variation case respectively. Initial mean trait values were sampled according to parameter values given in table S1.

(5) The authors use a perturbation regime that consists of increasing the density of a single species by a constant factor for a fixed duration of time. However, this perturbation regime may not be very realistic or representative of the types of disturbances that occur in natural or human-modified systems. For example, the perturbation may be stochastic, spatially heterogeneous, or affect multiple species simultaneously. The authors should explore the effects of different types of perturbations on network recovery, such as random, targeted, or cascading perturbations, and compare them with their baseline perturbation regime. They should also discuss the ecological and management implications of their perturbation regime and how it relates to real-world scenarios of network restoration.

Reply: The reviewer has raised an important point. In accordance with the reviewer's point, we conducted additional simulations comparing our generalist-species-based perturbation with targeted, and random perturbations. However, we were unclear about the reviewer's definition of cascading perturbation. In our single-species-focused perturbation, the perturbation cascades over time, spreading to different species due to positive feedback loops. Thus, our baseline one-species perturbation could be considered a cascading perturbation.

We compared our single-species perturbation with targeted and random perturbations. For the targeted perturbation, we selected the three species with the highest betweenness centrality in the network and used these as the focal species for perturbation over a fixed duration. For the random perturbation, we randomly chose one species from the plant-pollinator network and perturbed it for a fixed duration. As expected, the targeted perturbation performed better than our baseline perturbation (degree in figure below), while the random perturbation was the least effective in resurrecting collapsed networks. We present a figure illustrating these results below and discuss them in the methods section now after two lines after equation 4 in the main-text:

> "To provide a comparison, we also implemented two other forms of perturbation: "targeted perturbation" and "random perturbation." In the targeted perturbation, we selected the three species with the highest centrality values for the perturbation regime. In the random perturbation, we randomly chose one species to perturb ."

And we discuss this in detail in the discussion section in lines 480-499:

> "Focusing on a single species is less resource-intensive than targeting multiple species, which involves higher financial and ecological costs. However, targeting multiple species increases the likelihood of successful revival. If a targeted perturbation of one species fails due to unforeseen circumstances, other species may still support the recovery process. Therefore, one effective strategy may be a targeted perturbation, selecting species based on their roles in the community, such as their centrality. In our study, targeted perturbation of the three species with the highest centrality in the plant-pollinator network led to faster recovery than the baseline approach of choosing a single species with the most interactions (Fig. S20-S23). Out of the 115 networks analyzed, 106 had plants as the most generalist species. Plants, being immobile, are easier to handle for targeted perturbation compared to pollinators. Therefore, pollinators are not ideal for targeted perturbation. Instead, focusing on increasing

the density of generalist plants could be a more effective strategy for recovering collapsed networks (Menz et al. 2011, Devoto et al. 2012) in addition to restoration of habitats (Kaiser-Bunbury et al. 2017, Williams 2011). As shown in empirical studies, increasing the density of one or more generalist plant (Harmon-Threatt and Hendrix 2015, Wilkerson et al. 2014), or choosing plant species based on high centrality values could be key, as they support diverse pollinator populations and could aid the persistence of rare plants (Wilkerson et al. 2014). In comparison to our modelling study, previous studies have indicated that introducing species that could be generalists has positive impact on the structure of plant-pollinator communities (Russo et al. 2014, Bjerknes et al. 2007), and further could help in restoring these communities (Devoto et al. 2012)."

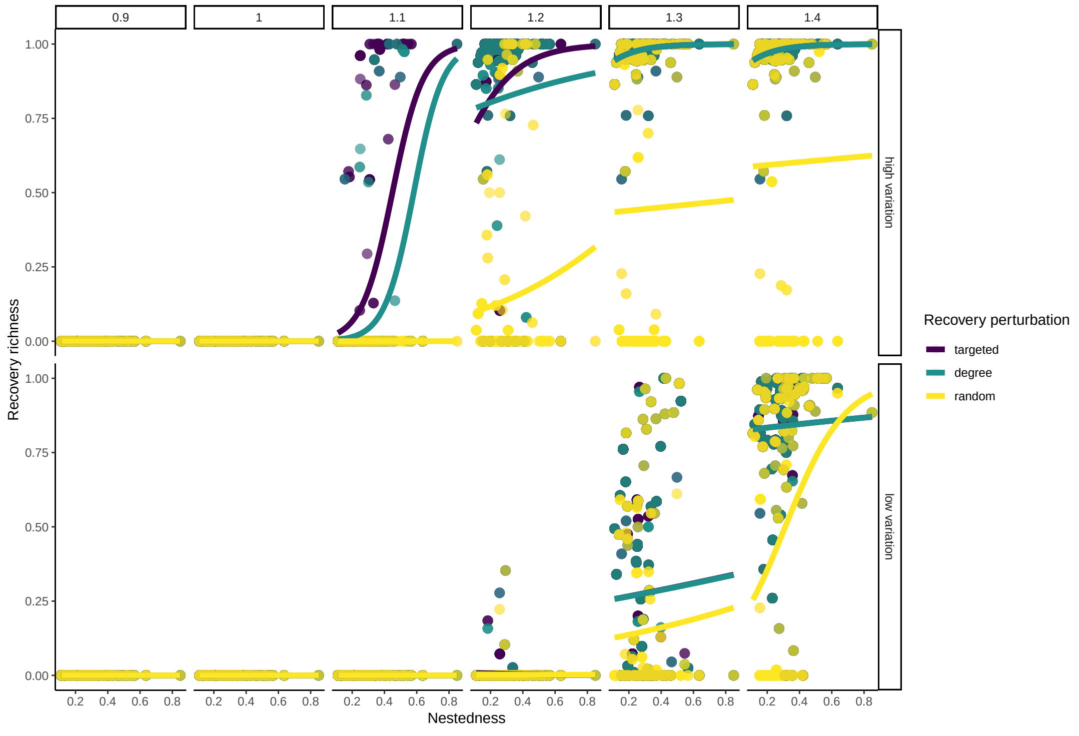

Figure S22: Impact of plant-pollinator network nestedness and trait variation on plant-pollinator density for three different types of perturbation (degree-based, random and targeted), for different thresholds of mutualistic strength γ_0 of 0.9, 1,1.1 and 1.2, 1.3, 1.4, and for two levels of trait-variation. Degree-based was based on perturbation that is used in the main-text. Random perturbation entailed randomly choosing a species in a network and perturbing the species for 500 time points for a forcing strength of 0.5. Finally, targeted perturbation entailed choosing two species based on betweenness centrality measure and perturbing those two species for a period of 500 time points with strength of 0.5. As nestedness (NODF) increased proportion of species with density greater than 0.5 increased specifically for targeted and degree-based. To start with, the networks were collated from web-of-life database and species had an initial density of $N_i < 0.005$, and perturbation/forcing strength of 0.5 was applied to the species with the highest degree for a duration of 500 time points. For high trait variation σ_i for all species was fixed at 0.02. Initial mean trait values were sampled as given in table 1.Different colored lines representing generalised linear model fitting with quasibinomial error distributions.

(6) The authors do not provide a clear definition or operationalization of the concept of network revival which is the first keyword. They seem to use different criteria and thresholds to measure the recovery of the network, such as the proportion of species with density greater than 0.5, the mean pollinator density, the mean plant density, and the mean trait values. However, they do not justify why they chose these criteria and thresholds, and how they relate to the original functionality and diversity of the network. The authors should provide a clear and consistent definition of network revival and explain how they measured it.

Reply: We thank the reviewer for this comment. We apologise for the inconsistency. We have now clarified in the methods section what we mean by a species recovery in a network. Now in lines 274-278 in the main-text:

> "We count a species that was not directly perturbed as recovered if the density of the species reached 0.5 from a density of less than 0.005, after the perturbation was stopped. In that way, we track the proportion of species that recovered to a density of 0.5 for each of the 115 plant-pollinator networks. In addition, we also tracked species density after the perturbation was stopped, and the mean and total biomass of networks reached."

In addition, given our modelling framework, we were still able to estimate mean plant and pollinator density, even if most species in a network did not reach a density greater than 0.5. We are unsure where we mentioned mean trait values as a criterion for species recovery in a network.

(7) The introduction is well-written, but it could be improved by providing more background and context on the problem of network collapse and restoration. The authors should cite more relevant studies on the causes, consequences, and indicators of network collapse, as well as the challenges and strategies of network restoration. They should also state more clearly the main objectives and hypotheses of their study.

Reply: We now have provided additional background on restoration. In this particular case, we have added new references such as Harmon-Threatt and Hendrix (2015),Kaiser-Bunbury et al. (2017),Harmon-Threatt (2020). The first reference is on habitat restoration, the second and third is on restoration strategies involving pollinators. This is now detailed in lines 43-57:

"In the event of an abrupt transition, one potential solution is to restore the original environmental conditions where feasible. For instance, restoration of habitats by removing invasive plant species could potentially increase pollinator diversity (Kaiser-Bunbury et al. 2017, Menz et al. 2011). However, this approach may not always be effective (Suding and Hobbs 2009, Harmon-Threatt 2020, Harmon-Threatt and Hendrix 2015). Often, we lack sufficient information or understanding of the system's properties at the brink of collapse. Additionally, ecological systems may exhibit hysteresis, where they can exist in both functional and dysfunctional states under the same environmental conditions, complicating the recovery process (Suding and Hobbs 2009, Isbell et al. 2013, Jiang et al. 2019). Consequently, even when previous conditions are restored, the system may never fully recover and could remain in the undesirable state. When a complex ecological community collapses, restoration strategies carry additional risks. For example, attempting to restore or translocate pollinators to revive plant-pollinator interactions may not always be successful, as many pollinators are inefficient at effective pollen transfer, potentially hindering the recovery of these communities (Menz et al. 2011)."

We also have now cited more literature on indicators of population and community collapses in the introduction such as Dai et al. (2012), Drake and Griffen (2010), Wissel (1984), Hastings and Wysham (2010). We have now re-written this in the introduction lines 32-43 :

"Shifts to undesirable states, such as collapses, are primarily caused by external environmental drivers crossing critical thresholds. These pressures include habitat loss, pollution, over-harvesting, and climate change, which can lead to rapid population and community declines. Consequently, there is an urgent need to forecast these rapid declines to preserve biodiversity and the ecosystem functions associated with these ecological communities. Phenomenological statistical tools such as early warning signals have been suggested to help in forecasting such unwanted transitions (Dakos et al. 2008, Scheffer et al. 2012, Baruah 2023, Baruah et al. 2019, Dai et al. 2012, Wissel 1984, Drake and Griffen 2010). These early warning signals have been suggested to be useful in predicting population collapses, or community-wide transitions as environmental conditions cross a definite threshold. However, there are many drawbacks associated with using such tools as most often these signals forecast

transition imperfectly (Boerlijst et al. 2013, Arkilanian et al. 2020).”

In the final paragraph, we have now added the main objectives of the study in lines 111-119 as:

> “In this study, using a dynamical eco-evolutionary framework, we try to revive mutualistic networks from an undesirable alternative stable state to a high-functioning stable state at unfavorable environmental conditions. We investigate how the original architecture of plant-pollinator networks and the presence of trait variation can facilitate the recovery of collapsed networks. Thus the objectives of this study are threefold: first, to evaluate whether complex plant-pollinator networks exhibit hysteresis, thereby affecting their recovery when environmental conditions are restored; second, to determine if these collapsed networks can be revived from a single species even under unfavorable environmental conditions; and third, to assess whether the architecture of plant-pollinator networks and trait variation influence the revival of collapsed networks.”

(8) The discussion section is insightful, but it could be more balanced and critical. The authors should acknowledge the limitations and uncertainties of their study, and discuss the potential sources of error or bias in their model and analysis. They should also compare and contrast their results with those of other studies, and suggest directions for future research.

Reply: We have now added four concise paragraphs: the first details the limitations and uncertainties of our results, and the second and third explores potential pitfalls of our modeling framework and future research directions, and the fourth showcases other studies in line with ours. These additions can be found in the revised manuscript on lines 517-526:

> “It is to be noted that such proposed revival strategies could have unintended consequences, often failing due to unforeseen factors. For instance, restoring generalist plant numbers might impact soil microbial communities, potentially increasing or decreasing competition with other plants and impacting recovery (Hortal et al. 2017, Raynaud and Leadley 2004). In addition, one other possible long-term risk would be

pollinator rewiring (CaraDonna et al. 2017). Increasing generalist plant density could cause pollinators to rewire and specialise on these plants, which in the long-term could reduce their interactions with other specialist plants and ultimately could impact ecosystem diversity. Our study does not address these issues, but future research could explore the consequences of adaptive rewiring of pollinators in plant-pollinator during network restoration."

Further in lines 527-535 in the discussion:

"Our modeling framework did not account for species dispersal or spatial structure, both of which can significantly influence revival strategies for collapsed plant-pollinator networks. Dispersal is known to rescue declining populations, enhance productivity, and provide spatial insurance in fragmented landscapes (Loreau et al. 2003, Thompson and Gonzalez 2017). Incorporating dispersal into our model could potentially lead to faster recovery. Additionally, the spatial arrangement of habitat patches could impact recovery, as the structure of habitat patches influences the metacommunity dynamics and process (Thompson and Gonzalez 2017). One important avenue for future research would be to evaluate how spatial arrangement of habitat patches could interacts with restoration strategies."

And in lines 536-546:

"We also assumed that trait distributions remain normal, based on our quantitative genetics framework where variance stays constant despite selection on mean traits. In reality, trait distributions could deviate from normality. Relaxing this assumption would require a computationally intensive individual-based approach. Furthermore, we assumed that trait variance is fixed and does not evolve (Barabás et al. 2022). This assumption may not hold under strong selective pressures to match partner traits. We used low trait variance to mimic low population density conditions. However, if phenotypic variance evolves under strong species-focused perturbation, selection to match multiple partners' traits could increase variance, potentially aiding in faster recovery. Once the network recovers and species traits align, stabilizing selection on the mean could reduce variance. This hypothesis requires further detailed evaluation, particularly on how species' trait variance evolves in response to targeted or species-focused perturbation."

And finally we compare our results with other studies qualitatively in lines 490-499:

> "Instead, focusing on increasing the density of generalist plants could be a more effective strategy for recovering collapsed networks (Menz et al. 2011, Devoto et al. 2012) in addition to restoration of habitats (Kaiser-Bunbury et al. 2017, Williams 2011). As shown in empirical studies, increasing the density of one or more generalist plant (Harmon-Threatt and Hendrix 2015, Wilkerson et al. 2014), or choosing plant species based on high centrality values could be key, as they support diverse pollinator populations and could aid the persistence of rare plants (Wilkerson et al. 2014). In comparison to our modelling study, previous studies have indicated that introducing species that could be generalists has positive impact on the structure of plant-pollinator communities (Russo et al. 2014, Bjerknes et al. 2007), and further could help in restoring these communities (Devoto et al. 2012)."

Referee: 2

This paper presents a well-written and comprehensive analysis of the revival of collapsed ecological networks, particularly focusing on the intricate dynamics of plant-pollinator interactions. The study addresses a significant ecological problem with depth and relevance, offering valuable insights into strategies for restoring and maintaining the functionality of mutualistic networks in the face of environmental challenges. The authors utilize eco-evolutionary dynamical modeling to investigate the revival of collapsed ecological networks, emphasizing the significance of network architecture and individual variation in managing recovery. Despite efforts to restore original environmental conditions, hysteresis often prevents full recovery, especially in complex mutualistic networks. Instead, the authors propose perturbing species with high interaction numbers, highlighting the importance of trait variation in facilitating network recovery. Overall, focusing on generalist species through positive perturbation emerges as a promising strategy to restore functionality to collapsed networks, even under unfavorable environmental conditions. My comments offer minor suggestions to further refine the quality of the literature.

Reply: We thank the reviewer for their appreciation and comments on the manuscript.

Comment1: In light of the study's focus on positive perturbation strategies targeting generalist species, how do the authors address the potential challenges associated with implementing large perturbations in generalist species compared to smaller perturbations across multiple species, particularly in terms of practical feasibility, ecological risk, and ecosystem resilience? The ecological meaning of forcing/perturbation is not clear in this article. In Figure 2, Figure 3 and other places, the author mentions $\gamma_0 = 1.15$ + forcing; sometimes it misleads. I suggest clarifying by stating 'With forcing at $\gamma_0 = 1.15$,' and another case could be 'without forcing.

Reply: We thank the reviewer for raising this point. Firstly, we have now adjusted both figure 2 and figure 3 mentioning "with forcing" and "with no forcing". Secondly, we have now discussed on the potential challenges on implementing large perturbations focused on a single species compared to multiple species with smaller perturbation in line 472-487:

> "Our study suggests that, without detailed demographic data, increasing the survival of a super generalist species might be the simplest approach to recovering a collapsed network. However, implementing a strong perturbation on a generalist species poses challenges. If the generalist is a pollinator, it may not be effective in pollen transfer, potentially hindering recovery(Menz et al. 2011). Additionally, simply increasing generalist pollinator abundance may not suffice, as they need more than floral resources to thrive (Harmon-Threatt 2020, Roulston and Goodell 2011, Brosi and Briggs 2013). Conversely, increasing the density of generalist plants could be key, as they support diverse pollinator populations and aid the persistence of rare plants (Harmon-Threatt and Hendrix 2015, Wilkerson et al. 2014). Focusing on a single species is less resource-intensive than targeting multiple species, which involves higher financial and ecological costs. However, targeting multiple species increases the likelihood of successful revival. If a targeted perturbation of one species fails due to unforeseen circumstances, other species may still support the recovery process. Therefore, one effective strategy may be a targeted perturbation, selecting species based on their roles in the community, such as their centrality. In our study, targeted perturbation of the three species with the highest centrality in the plant-pollinator

> network led to faster recovery than the baseline approach of choosing a single species with the most interactions (Fig. S20-S23)."

Comment2: The title of the paper, "Reviving collapsed ecological networks from a single species," accurately captures the main focus of the study on the revival of collapsed ecological networks using interventions targeting a single species. However, given that the paper specifically discusses plant-pollinator networks and not broader ecological networks, it may be more appropriate to refine the title to better reflect this specificity. A title such as "Reviving collapsed plant-pollinator networks from a single species" would provide readers with a clearer understanding of the specific ecological context under investigation and align more closely with the content of the paper.

Reply: "We have now changed the title of our paper to : "Reviving collapsed plant-pollinator networks from a single species."

Comment3: How might the effectiveness of positive perturbation strategies vary across different types of ecological networks (e.g., competitive, predator-prey) and under different environmental contexts?

Reply: This is an important point raised by the reviewer. We are currently evaluating theoretically how positive perturbation could impact recovery of collapsed food webs. This is still under preparation. However, we do discuss this in the discussion section. In the case of the predator-prey food-webs we have now discussed in the discussion section in lines 500-516:

> "The knowledge of the system, the type of species interactions, and the presence of feedback loops could be beneficial in managing the recovery of such networks (Suding et al. 2016). However, purely competitive communities or communities with predation could potentially constrain the recovery of collapsed networks. For instance, changes in trophic interactions that entail the removal of predators or prey might be important for a successful recovery of vegetation (Tanentzap et al. 2011, Hidding et al. 2013). The effectiveness of perturbing the most generalist species to revive a collapsed network thus could vary with the type of species interactions predominant in the community. In complex predator-prey food webs, recovery might not be always guaranteed, especially if only one basal resource species was perturbed. Recovery could depend not only on the number of perturbed basal species but also on the

proportion of predation links and the food web structure. Generally, a higher number of predation links could require positively perturbing more basal species for successful recovery from a collapsed state. Communities which harbour negative interactions such as competitive and predator-prey communities, would thus require different restoration strategies, as such communities have higher number of negative-feedback loops that suppresses the propagation of perturbation (Donohue et al. 2017). In addition, under harsh environmental conditions, recovery of such communities might be further hampered."

Comment4: While the author discusses strategies for network revival, it does not address the long-term sustainability or stability of the revived networks. Are there potential risks or challenges associated with the proposed revival strategies, such as the potential for destabilizing feedback loops or unintended consequences for ecosystem functioning?

Reply: We thank the reviewer for this comment. We do agree that there will always be potential unintended consequences in such revival strategies that could have negative impact on ecosystem functioning. We have now discussed this in lines 517-526:

> "It is to be noted that such proposed revival strategies could have unintended consequences, often failing due to unforeseen factors. For instance, restoring generalist plant numbers might impact soil microbial communities, potentially increasing or decreasing competition with other plants and impacting recovery (Hortal et al. 2017, Raynaud and Leadley 2004). In addition, one other possible long-term risk would be pollinator rewiring (CaraDonna et al. 2017). Increasing generalist plant density could cause pollinators to rewire and specialise on these plants, which in the long-term could reduce their interactions with other specialist plants and ultimately could impact ecosystem diversity. Our study does not address these issues, but future research could explore the consequences of adaptive rewiring of pollinators in plant-pollinator during network restoration."

Comment5: It is noteworthy that alterations in nestedness can lead to changes in connectance, modularity, and dimension within ecological networks. Consequently, attributing variations in results solely to nestedness becomes challenging. One potential approach to address this concern could involve controlling for dimension

and connectance while generating nestedness matrices. By verifying that the results remain consistent under these controlled conditions, it would bolster the confidence in attributing observed changes to nestedness. Alternatively, performing a principal component analysis (PCA) and incorporating nestedness, connectance, and dimension as variables could provide further insights into their individual contributions to the observed outcomes. This approach would enhance the robustness and interpretability of the study findings by identifying the main dimensions of variation and their relationships with the outcomes of interest. Additionally, incorporating the results obtained after generating nestedness matrices or discussing this methodological approach in the literature would strengthen the credibility of the study.

Reply: We thank the reviewer for this important comment and suggestion. In accordance with the reviewer's suggestion, we have now generated nestedness matrices keeping connectance and the size of the network constant, to specifically evaluate the impact of nestedness on the revival of collapsed networks. We found that the impact of nestedness was weakened when controlled for network size and connectance (Fig. S34). We have briefly discussed how we created artificial nestedness matrices in the supplementary appendix 5. We have now added this figure in the supplementary (Fig. S34) and discussed this in the discussion sections 461-467:

> "Note that nestedness in such networks correlates with network size and connectance. Thus, changes in nestedness leads to changes in connectance. We conducted additional analyses by fixing network size and connectance, and creating plant-pollinator interaction networks with nestedness varying from 1 to 0.38. Our findings confirmed that higher nestedness is still associated with higher recovery species richness, especially when species exhibit high trait variation. Although, this impact is slightly negative when trait variation was low when controlled for network size and connectance (Fig. S34)."

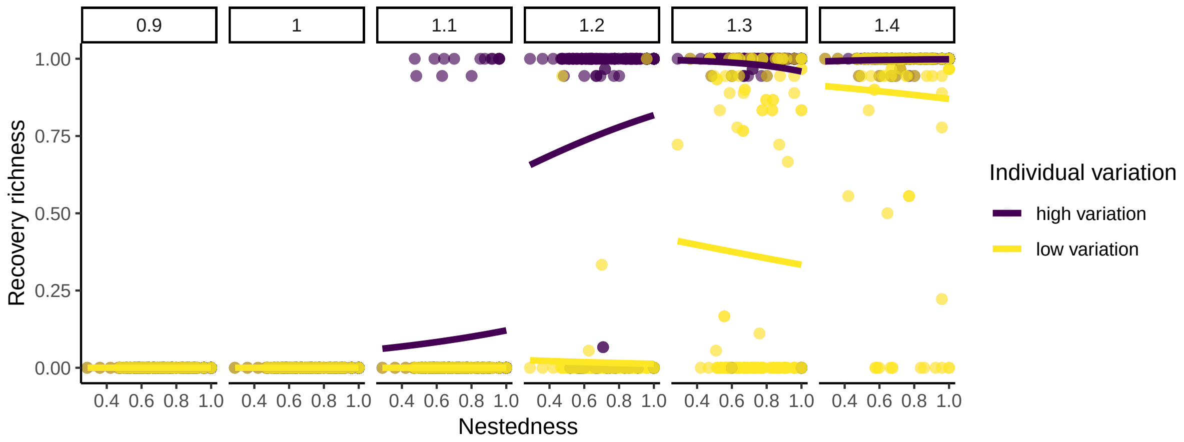

Figure S34: Network recovery from perturbing a single species was impacted positively impacted by nestedness shown for two levels of trait variation (rows: high variation, low variation), for different levels of threshold mutualistic strength , γ_0, of 0.9, 1, 1.1,1.2, 1.3 and 1.4 (columns). In all these networks, only the species with the highest degree, was positively perturbed from a very low density, $N_i < 0.005$, for a duration of 500 time points with a forcing strength of 0.5, while the rest of the species remained unperturbed. σ_i was fixed at 0.02 for the high trait variation case and 0.005 for the low trait variation case respectively. We observed that lower handling times lead to better recovery. Initial mean trait values were sampled according to parameter values given in table 1. Here, the network size was 30 species and connectance was 0.42, and then nestedness matrices keeping the connectance and network size constant were created that varied from a low of 0.38 to a high of 1. Different colored lines representing generalised linear model fitting with quasibinomial error distributions.

Comment6: Discussing the robustness of the results for nonzero b values would enhance the applicability and generalizability of the findings. Including a sensitivity analysis or exploring the effects of nonzero b values on network dynamics could provide valuable insights into how variations in species intrinsic growth rates interact with other factors, such as network architecture and perturbation strategies, to influence network resilience and recovery from collapse.

Reply: We thank the reviewer for this comment. We have now added a sensitivity analyses where we explored the effects of non-zero b values and network architecture on network recovery from a collapse state (attached below a Fig. S30, also in the appendix Fig. S30-S31). We have also discussed this in the results section as (now lines 381-383):

> "In addition, non-zero intrinsic growth rates impacted network recovery, to the point that having a higher growth rate allowed better recovery of collapsed networks than others (Fig. S30-S31)."

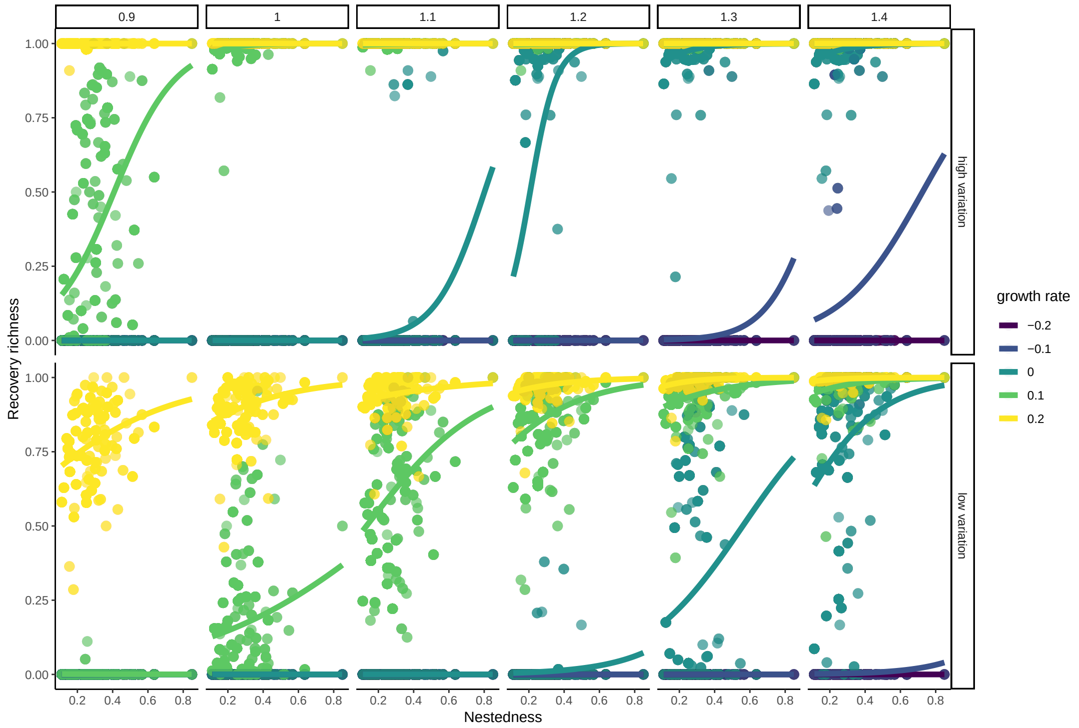

Figure S30: Impact of variation in growth rate, b_i, on network recovery from perturbing a single species. Network recovery from perturbing a single species was positively impacted by nestedness shown for two levels of trait variation (high variation, low variation), for five levels of threshold mutualistic strength, γ_0, of 0.9, 1, 1.1,1.2, 1.3, 1.4, and for five different growth rate values of -0.2,-0.1,0,0.1,0.2. Higher positive growth rate leads to better network recovery. Negative and zero growth rates would indicate that species are obligate mutualists. In all these networks, only the species with the highest degree, was positively perturbed from a very low density, $N_i < 0.005$, for a duration of 500 time points with a forcing strength of 0.5, while the rest of the species remained unperturbed. Shown here are data from 115 networks. σ_i was fixed at 0.02 for the high trait variation case and 0.005 for the low trait variation case respectively. Initial mean trait values were sampled according to parameter values given in table 1.Different colored lines representing generalised linear model fitting with quasibinomial error distributions.

Comment7: The discussion on single-species perturbation lacks clarity regarding whether the perturbation targets pollinators, plants, or both. Clarifying the focus of the perturbation, whether it pertains to pollinators, plants, or both, would provide greater insight into its potential effectiveness and utility in network resurrection. Considering the ecological roles and interactions of both pollinators and plants, specifying the targeted species for perturbation could help elucidate which approach may be more beneficial for network recovery. This clarification would enhance the comprehensibility and applicability of the study findings.

Reply: We thank the reviewer for this comment. In general, plants would be a better focus for species-specific perturbation, as some pollinators could be ineffective in pollen transfer. Also in our study, generalist species were mostly plants. Infact, 106 out of 115 networks had plants

as the generalist species. We have now discussed this and clarified this in the discussion section as (lines 473-499):

> "However, implementing a strong perturbation on a generalist species poses challenges. If the generalist is a pollinator, it may not be effective in pollen transfer, potentially hindering recovery (Menz et al. 2011). Additionally, simply increasing generalist pollinator abundance may not suffice, as they need more than floral resources to thrive (Harmon-Threatt 2020, Roulston and Goodell 2011, Brosi and Briggs 2013). Conversely, increasing the density of generalist plants could be key, as they support diverse pollinator populations and aid the persistence of rare plants (Harmon-Threatt and Hendrix 2015, Wilkerson et al. 2014). Focusing on a single species is less resource-intensive than targeting multiple species, which involves higher financial and ecological costs. However, targeting multiple species increases the likelihood of successful revival. If a targeted perturbation of one species fails due to unforeseen circumstances, other species may still support the recovery process. Therefore, one effective strategy may be a targeted perturbation, selecting species based on their roles in the community, such as their centrality. In our study, targeted perturbation of the three species with the highest centrality in the plant-pollinator network led to faster recovery than the baseline approach of choosing a single species with the most interactions (Fig. S20-S23). Out of the 115 networks analyzed, 106 had plants as the most generalist species. Plants, being immobile, are easier to handle for targeted perturbation compared to pollinators. Therefore, pollinators are not ideal for targeted perturbation. Instead, focusing on increasing the density of generalist plants could be a more effective strategy for recovering collapsed networks (Menz et al. 2011, Devoto et al. 2012) in addition to restoration of habitats (Kaiser-Bunbury et al. 2017, Williams 2011). As shown in empirical studies, increasing the density of one or more generalist plant (Harmon-Threatt and Hendrix 2015, Wilkerson et al. 2014), or choosing plant species based on high centrality values could be key, as they support diverse pollinator populations and could aid the persistence of rare plants (Wilkerson et al. 2014). In comparison to our modelling study, previous studies have indicated that introducing species that could be generalists has positive impact on the structure of plant-pollinator communities (Russo et al. 2014, Bjerknes et al. 2007), and further could help in restoring these communities (Devoto et al. 2012)."

Comment8: The absence of references or empirical data for certain parameter values, such as $h^2 = 0.4$, raises concerns about the validity and reliability of the model assumptions. It would greatly enhance the credibility and transparency of the study's findings if supporting evidence or experimental data for these parameter values were provided.

Reply: We have in accordance with the reviewer added the justifications of using such a heritability value which was within the range of heritabilities observed in empirical plant-pollinator studies in nature. In addition, we have also done sensitivity analyses with different heritability values that could potentially impact network recovery. Now in lines 182-188:

> "We fixed heritability,h^2, at 0.4. In plant-pollinator empirical studies, heritability of morphological traits of pollinators that are involved in pollination such as the proboscis was as high as 0.75 with other morphological traits ranged from 0.1 to 0.9 (Koffler et al. 2017). For plant floral morphology, heritability was also very high with a median value of 0.55, which thus was around the value we used in our modelling framework (Romero-Bravo and Castellanos, Kaczorowski et al. 2008)."

Comment9: The mention of strategies such as maintaining the abundance of an influential pollinator and setting the decay rate of another influential pollinator to zero is attributed to the paper "Harnessing tipping points in complex ecological networks." Please cite this paper and mention the results.

Reply: We apologise for missing this important reference. We have now included this paper in our reference, now in methods two sentences after equation 4 in the main-text.

Comment10: The discussion should include future directions or potential applications of the research problem to provide insights for future studies and practical implementations. Incorporating these considerations can enrich the discussion and enhance the relevance of the study's findings.

Reply: We have now included future direction and potential research questions that could follow from our study in lines 527-535:

> "Our modeling framework did not account for species dispersal or spatial structure, both of which can significantly influence revival strategies for collapsed plant-pollinator networks. Dispersal is known to rescue declining populations, enhance

productivity, and provide spatial insurance in fragmented landscapes (Loreau et al. 2003, Thompson and Gonzalez 2017). Incorporating dispersal into our model could potentially lead to faster recovery. Additionally, the spatial arrangement of habitat patches could impact recovery, as the structure of habitat patches influences the metacommunity dynamics and processes (Thompson and Gonzalez 2017). One important avenue for future research would be to evaluate how spatial arrangement of habitat patches could interacts with restoration strategies."

In addition to the above, we also mention further directions of future research in discussion now in lines 520-526 :

"In addition, one other possible long-term risk would be pollinator rewiring (CaraDonna et al. 2017). Increasing generalist plant density could cause pollinators to rewire and specialise on these plants, which in long-term could reduce their interactions with other specialist plants that ultimately could impact ecosystem diversity. Our study does not address these issues, but future research could explore the consequences of adaptive rewiring of pollinators in plant-pollinator during network restoration."

And finally in lines 536-546:,

"We also assumed that trait distributions remain normal, based on our quantitative genetics framework where variance stays constant despite selection on mean traits. In reality, trait distributions could deviate from normality. Relaxing this assumption would require a computationally intensive individual-based approach. Furthermore, we assumed that trait variance is fixed and does not evolve (Barabás et al. 2022). This assumption may not hold under strong selective pressures to match partner traits. We used low trait variance to mimic low population density conditions. However, if phenotypic variance evolves under strong species-focused perturbation, selection to match multiple partners' traits could increase variance, potentially aiding in faster recovery. Once the network recovers and species traits align, stabilizing selection on the mean could reduce variance. This hypothesis requires further detailed evaluation, particularly on how species' trait variance evolves in response to targeted or species-focused perturbation."

Comment11: **Correction suggested: Adjust the interval for** u_i to $[-0.5, 0.5]$ **in line 230.**

Reply: We have now corrected this.

I also think the authors should make all of their code available, so others can reproduce their work and study variations. I don't think this has been done.

Reply: The code and scripts were all made available in the previous version, and also in this version, please refer to the main page of the manuscript for the Github repository, also attached here `https://github.com/GauravKBaruah/04-Network_revival_git`.

References

Mário Almeida-Neto, Paulo Guimarães, Paulo R. Guimarães, Rafael D. Loyola, and Werner Ulrich. A consistent metric for nestedness analysis in ecological systems: reconciling concept and measurement. *Oikos*, 117(8):1227–1239, 2008. ISSN 1600-0706. doi: 10.1111/j.0030-1299.2008.16644.x. URL `https://onlinelibrary.wiley.com/doi/abs/10.1111/j.0030-1299.2008.16644.x`.

A. A. Arkilanian, C. F. Clements, A. Ozgul, and G. Baruah. Effect of time series length and resolution on abundance- and trait-based early warning signals of population declines. *Ecology*, 101(7):e03040, 2020. ISSN 1939-9170. doi: 10.1002/ecy.3040. URL `https://onlinelibrary.wiley.com/doi/abs/10.1002/ecy.3040`. _eprint: https://onlinelibrary.wiley.com/doi/pdf/10.1002/ecy.3040.

György Barabás, Matthew J. Michalska-Smith, and Stefano Allesina. Self-regulation and the stability of large ecological networks. *Nature Ecology & Evolution*, 1(12):1870–1875, December 2017. ISSN 2397-334X. doi: 10.1038/s41559-017-0357-6. URL `https://www.nature.com/articles/s41559-017-0357-6`. Number: 12 Publisher: Nature Publishing Group.

György Barabás, Christine Parent, Andrew Kraemer, Frederik Van de Perre, and Frederik De Laender. The evolution of trait variance creates a tension between species diversity and functional diversity. *Nature Communications*, 13(1):2521, May 2022. ISSN 2041-1723. doi: 10.1038/s41467-022-30090-4. URL `https://www.nature.com/articles/s41467-022-30090-4`. Number: 1 Publisher: Nature Publishing Group.

Gaurav Baruah. The impact of individual variation on abrupt collapses in mutualistic networks. *Ecology Letters*, 25(1):26–37, 2022. ISSN 1461-0248. doi: 10.1111/ele.13895. URL https://onlinelibrary.wiley.com/doi/abs/10.1111/ele.13895. _eprint: https://onlinelibrary.wiley.com/doi/pdf/10.1111/ele.13895.

Gaurav Baruah. Transitions and its indicators in mutualistic meta-networks: effects of network topology, size of metacommunities and species dispersal. *Evolutionary Ecology*, 37(4):691–708, August 2023. ISSN 1573-8477. doi: 10.1007/s10682-023-10239-3. URL https://doi.org/10.1007/s10682-023-10239-3.

Gaurav Baruah, Christopher F. Clements, Frédéric Guillaume, and Arpat Ozgul. When Do Shifts in Trait Dynamics Precede Population Declines? *The American Naturalist*, 193(5): 633–644, May 2019. ISSN 0003-0147. doi: 10.1086/702849. URL https://www.journals.uchicago.edu/doi/abs/10.1086/702849. Publisher: The University of Chicago Press.

Anne-Line Bjerknes, Ørjan Totland, Stein Joar Hegland, and Anders Nielsen. Do alien plant invasions really affect pollination success in native plant species? *Biological Conservation*, 138(1):1–12, August 2007. ISSN 0006-3207. doi: 10.1016/j.biocon.2007.04.015. URL https://www.sciencedirect.com/science/article/pii/S0006320707001693.

Maarten C. Boerlijst, Thomas Oudman, and André M. de Roos. Catastrophic Collapse Can Occur without Early Warning: Examples of Silent Catastrophes in Structured Ecological Models. *PLoS ONE*, 8(4), 2013.

Berry J. Brosi and Heather M. Briggs. Single pollinator species losses reduce floral fidelity and plant reproductive function. *Proceedings of the National Academy of Sciences*, 110(32): 13044–13048, August 2013. doi: 10.1073/pnas.1307438110. URL https://www.pnas.org/doi/full/10.1073/pnas.1307438110. Publisher: Proceedings of the National Academy of Sciences.

Paul J. CaraDonna, William K. Petry, Ross M. Brennan, James L. Cunningham, Judith L. Bronstein, Nickolas M. Waser, and Nathan J. Sanders. Interaction rewiring and the rapid turnover of plant–pollinator networks. *Ecology Letters*, 20(3):385–394, 2017. ISSN 1461-0248. doi: 10.1111/ele.12740. URL https://onlinelibrary.wiley.com/doi/abs/10.1111/ele.12740.

L. Dai, D. Vorselen, K. S. Korolev, and J. Gore. Generic Indicators for Loss of Resilience Before a Tipping Point Leading to Population Collapse. *Science*, 336(6085):1175–1177, 2012. ISSN 1095-9203 (Electronic)\textbackslashtextbackslashtextbackslashr0036-8075 (Linking). doi: 10.1126/science.1219805.

Vasilis Dakos, Marten Scheffer, Egbert H van Nes, Victor Brovkin, Vladimir Petoukhov, and Hermann Held. Slowing down as an early warning signal for abrupt climate change. *Proceedings of the National Academy of Sciences of the United States of America*, 105(38):14308–12, 2008. ISSN 1091-6490 (Electronic)\textbackslashtextbackslashtextbackslashn0027-8424 (Linking). doi: 10.1073/pnas.0802430105. URL `http://www.pubmedcentral.nih.gov/articlerender.fcgi?artid=2567225&tool=pmcentrez&rendertype=abstract`.

Mariano Devoto, Sallie Bailey, Paul Craze, and Jane Memmott. Understanding and planning ecological restoration of plant–pollinator networks. *Ecology Letters*, 15(4):319–328, 2012. ISSN 1461-0248. doi: 10.1111/j.1461-0248.2012.01740.x. URL `https://onlinelibrary.wiley.com/doi/abs/10.1111/j.1461-0248.2012.01740.x`. _eprint: https://onlinelibrary.wiley.com/doi/pdf/10.1111/j.1461-0248.2012.01740.x.

Ian Donohue, Owen L. Petchey, Sonia Kéfi, Alexandre Génin, Andrew L. Jackson, Qiang Yang, and Nessa E. O'Connor. Loss of predator species, not intermediate consumers, triggers rapid and dramatic extinction cascades. *Global Change Biology*, 23(8):2962–2972, 2017. ISSN 1365-2486. doi: 10.1111/gcb.13703. URL `https://onlinelibrary.wiley.com/doi/abs/10.1111/gcb.13703`. _eprint: https://onlinelibrary.wiley.com/doi/pdf/10.1111/gcb.13703.

John M Drake and Blaine D Griffen. Early warning signals of extinction in deteriorating environments. *Nature*, 467(7314):456–459, 2010. ISSN 0028-0836. doi: 10.1038/nature09389.

Paulo R. Guimarães, Pedro Jordano, and John N. Thompson. Evolution and coevolution in mutualistic networks. *Ecology Letters*, 14(9):877–885, 2011. ISSN 1461-0248. doi: https://doi.org/10.1111/j.1461-0248.2011.01649.x. URL `https://onlinelibrary.wiley.com/doi/abs/10.1111/j.1461-0248.2011.01649.x`.

Paulo R. Guimarães, Mathias M. Pires, Pedro Jordano, Jordi Bascompte, and John N. Thompson. Indirect effects drive coevolution in mutualistic networks. *Nature*, 550(7677):511–514, October 2017. ISSN 1476-4687. doi: 10.1038/nature24273. URL `https://www.nature.com/articles/nature24273`. Publisher: Nature Publishing Group.

Alexandra Harmon-Threatt. Influence of Nesting Characteristics on Health of Wild Bee Communities. *Annual Review of Entomology*, 65(Volume 65, 2020):39–56, January 2020. ISSN 0066-4170, 1545-4487. doi: 10.1146/annurev-ento-011019-024955. URL https://www.annualreviews.org/content/journals/10.1146/annurev-ento-011019-024955. Publisher: Annual Reviews.

Alexandra N. Harmon-Threatt and Stephen D. Hendrix. Prairie restorations and bees: The potential ability of seed mixes to foster native bee communities. *Basic and Applied Ecology*, 16(1):64–72, February 2015. ISSN 1439-1791. doi: 10.1016/j.baae.2014.11.001. URL https://www.sciencedirect.com/science/article/pii/S1439179114001492.

Alan Hastings and Derin B. Wysham. Regime shifts in ecological systems can occur with no warning. *Ecology Letters*, 13(4):464–472, 2010. ISSN 1461-0248. doi: 10.1111/j.1461-0248.2010.01439.x. URL https://onlinelibrary.wiley.com/doi/abs/10.1111/j.1461-0248.2010.01439.x. _eprint: https://onlinelibrary.wiley.com/doi/pdf/10.1111/j.1461-0248.2010.01439.x.

Bert Hidding, Jean-Pierre Tremblay, and Steeve D. Côté. A large herbivore triggers alternative successional trajectories in the boreal forest. *Ecology*, 94(12):2852–2860, 2013. ISSN 1939-9170. doi: 10.1890/12-2015.1. URL https://onlinelibrary.wiley.com/doi/abs/10.1890/12-2015.1. _eprint: https://onlinelibrary.wiley.com/doi/pdf/10.1890/12-2015.1.

S. Hortal, Y. M. Lozano, F. Bastida, C. Armas, J. L. Moreno, C. Garcia, and F. I. Pugnaire. Plant-plant competition outcomes are modulated by plant effects on the soil bacterial community. *Scientific Reports*, 7(1):17756, December 2017. ISSN 2045-2322. doi: 10.1038/s41598-017-18103-5. URL https://www.nature.com/articles/s41598-017-18103-5. Publisher: Nature Publishing Group.

Forest Isbell, David Tilman, Stephen Polasky, Seth Binder, and Peter Hawthorne. Low biodiversity state persists two decades after cessation of nutrient enrichment. *Ecology Letters*, 16(4):454–460, 2013. ISSN 1461-0248. doi: 10.1111/ele.12066. URL https://onlinelibrary.wiley.com/doi/abs/10.1111/ele.12066. _eprint: https://onlinelibrary.wiley.com/doi/pdf/10.1111/ele.12066.

Junjie Jiang, Alan Hastings, and Ying-Cheng Lai. Harnessing tipping points in complex ecological networks. *Journal of The Royal Society Interface*, 16(158):20190345, September 2019. doi:

10.1098/rsif.2019.0345. URL https://royalsocietypublishing.org/doi/10.1098/rsif.2019.0345. Publisher: Royal Society.

Christopher A. Johnson, Proneet Dutt, and Jonathan M. Levine. Competition for pollinators destabilizes plant coexistence. *Nature*, 607(7920):721–725, July 2022. ISSN 1476-4687. doi: 10.1038/s41586-022-04973-x. URL https://www.nature.com/articles/s41586-022-04973-x. Number: 7920 Publisher: Nature Publishing Group.

Rainee L. Kaczorowski, Thomas E. Juenger, and Timothy P. Holtsford. Heritability and Correlation Structure of Nectar and Floral Morphology Traits in Nicotiana Alata. *Evolution*, 62(7):1738–1750, 2008. ISSN 1558-5646. doi: 10.1111/j.1558-5646.2008.00400.x. URL https://onlinelibrary.wiley.com/doi/abs/10.1111/j.1558-5646.2008.00400.x. _eprint: https://onlinelibrary.wiley.com/doi/pdf/10.1111/j.1558-5646.2008.00400.x.

Christopher N. Kaiser-Bunbury, James Mougal, Andrew E. Whittington, Terence Valentin, Ronny Gabriel, Jens M. Olesen, and Nico Blüthgen. Ecosystem restoration strengthens pollination network resilience and function. *Nature*, 542(7640):223–227, February 2017. ISSN 1476-4687. doi: 10.1038/nature21071. URL https://www.nature.com/articles/nature21071. Number: 7640 Publisher: Nature Publishing Group.

Saskia G.T. Klumpers, Martina Stang, and Peter G.L. Klinkhamer. Foraging efficiency and size matching in a plant–pollinator community: the importance of sugar content and tongue length. *Ecology Letters*, 22(3):469–479, 2019. ISSN 1461-0248. doi: 10.1111/ele.13204. URL https://onlinelibrary.wiley.com/doi/abs/10.1111/ele.13204. _eprint: https://onlinelibrary.wiley.com/doi/pdf/10.1111/ele.13204.

Sheina Koffler, Astrid de Matos Peixoto Kleinert, and Rodolfo Jaffé. Quantitative conservation genetics of wild and managed bees. *Conservation Genetics*, 18(3):689–700, June 2017. ISSN 1572-9737. doi: 10.1007/s10592-016-0904-8. URL https://doi.org/10.1007/s10592-016-0904-8.

Michel Loreau, Nicolas Mouquet, and Andrew Gonzalez. Biodiversity as spatial insurance in heterogeneous landscapes. *Proceedings of the National Academy of Sciences*, 100(22):12765–12770, October 2003. doi: 10.1073/pnas.2235465100. URL https://www.pnas.org/doi/full/10.1073/pnas.2235465100. Publisher: Proceedings of the National Academy of Sciences.

Myles H. M. Menz, Ryan D. Phillips, Rachael Winfree, Claire Kremen, Marcelo A. Aizen, Steven D. Johnson, and Kingsley W. Dixon. Reconnecting plants and pollinators: challenges in the restoration of pollination mutualisms. *Trends in Plant Science*, 16(1):4–12, January 2011. ISSN 1360-1385. doi: 10.1016/j.tplants.2010.09.006. URL https://www.sciencedirect.com/science/article/pii/S1360138510001962.

M. E. J. Newman. Modularity and community structure in networks. *Proceedings of the National Academy of Sciences*, 103(23):8577–8582, June 2006. doi: 10.1073/pnas.0601602103. URL https://www.pnas.org/doi/abs/10.1073/pnas.0601602103. Publisher: Proceedings of the National Academy of Sciences.

Xavier Raynaud and Paul W. Leadley. Soil Characteristics Play a Key Role in Modeling Nutrient Competition in Plant Communities. *Ecology*, 85(8):2200–2214, 2004. ISSN 1939-9170. doi: 10.1890/03-0817. URL https://onlinelibrary.wiley.com/doi/abs/10.1890/03-0817. _eprint: https://onlinelibrary.wiley.com/doi/pdf/10.1890/03-0817.

Andrés Romero-Bravo and Maria Clara Castellanos. Nectar and floral morphology differ in evolutionary potential in novel pollination environments. *New Phytologist*, n/a(n/a). ISSN 1469-8137. doi: 10.1111/nph.19780. URL https://onlinelibrary.wiley.com/doi/abs/10.1111/nph.19780. _eprint: https://onlinelibrary.wiley.com/doi/pdf/10.1111/nph.19780.

T'ai H. Roulston and Karen Goodell. The Role of Resources and Risks in Regulating Wild Bee Populations. *Annual Review of Entomology*, 56(Volume 56, 2011):293–312, January 2011. ISSN 0066-4170, 1545-4487. doi: 10.1146/annurev-ento-120709-144802. URL https://www.annualreviews.org/content/journals/10.1146/annurev-ento-120709-144802. Publisher: Annual Reviews.

Laura Russo, Jane Memmott, Daniel Montoya, Katriona Shea, and Yvonne M. Buckley. Patterns of introduced species interactions affect multiple aspects of network structure in plant–pollinator communities. *Ecology*, 95(10):2953–2963, 2014. ISSN 1939-9170. doi: 10.1890/13-2229.1. URL https://onlinelibrary.wiley.com/doi/abs/10.1890/13-2229.1. _eprint: https://onlinelibrary.wiley.com/doi/pdf/10.1890/13-2229.1.

Amol Sasane. *A Friendly Approach to Functional Analysis | Mathematical Association of America*, volume 1. 2017. URL https://maa.org/press/maa-reviews/a-friendly-approach-to-functional-analysis.

M. Scheffer, S. R. Carpenter, T. M. Lenton, J. Bascompte, W. Brock, V. Dakos, J. van de Koppel, I. a. van de Leemput, S. a. Levin, E. H. van Nes, M. Pascual, and J. Vandermeer. Anticipating Critical Transitions. *Science*, 338(6105):344–348, 2012. ISSN 0036-8075. doi: 10.1126/science.1225244.

Katharine Suding, Erica Spotswood, Dylan Chapple, Erin Beller, and Katherine Gross. Ecological Dynamics and Ecological Restoration. In Margaret A. Palmer, Joy B. Zedler, and Donald A. Falk, editors, *Foundations of Restoration Ecology*, pages 27–56. Island Press/Center for Resource Economics, Washington, DC, 2016. ISBN 978-1-61091-698-1. doi: 10.5822/978-1-61091-698-1_2. URL `https://doi.org/10.5822/978-1-61091-698-1_2`.

Katharine N. Suding and Richard J. Hobbs. Threshold models in restoration and conservation: a developing framework. *Trends in Ecology & Evolution*, 24(5):271–279, May 2009. ISSN 0169-5347. doi: 10.1016/j.tree.2008.11.012. URL `https://www.sciencedirect.com/science/article/pii/S0169534709000470`.

Andrew J. Tanentzap, Dawn R. Bazely, Saewan Koh, Mika Timciska, Edward G. Haggith, Terry J. Carleton, and David A. Coomes. Seeing the forest for the deer: Do reductions in deer-disturbance lead to forest recovery? *Biological Conservation*, 144(1):376–382, January 2011. ISSN 0006-3207. doi: 10.1016/j.biocon.2010.09.015. URL `https://www.sciencedirect.com/science/article/pii/S0006320710004143`.

Patrick L. Thompson and Andrew Gonzalez. Dispersal governs the reorganization of ecological networks under environmental change. *Nature Ecology & Evolution*, 1(6):1–8, May 2017. ISSN 2397-334X. doi: 10.1038/s41559-017-0162. URL `https://www.nature.com/articles/s41559-017-0162`.

Thorsten Wiegand, Xugao Wang, Kristina J. Anderson-Teixeira, Norman A. Bourg, Min Cao, Xiuqin Ci, Stuart J. Davies, Zhanqing Hao, Robert W. Howe, W. John Kress, Juyu Lian, Jie Li, Luxiang Lin, Yiching Lin, Keping Ma, William McShea, Xiangcheng Mi, Sheng-Hsin Su, I.-Fang Sun, Amy Wolf, Wanhui Ye, and Andreas Huth. Consequences of spatial patterns for coexistence in species-rich plant communities. *Nature Ecology & Evolution*, 5(7):965–973, July 2021. ISSN 2397-334X. doi: 10.1038/s41559-021-01440-0. URL `https://www.nature.com/articles/s41559-021-01440-0`. Number: 7 Publisher: Nature Publishing Group.

Marit L. Wilkerson, Kimiora L. Ward, Neal M. Williams, Katharina S. Ullmann, and Truman P. Young. Diminishing Returns from Higher Density Restoration Seedings Suggest Trade-offs in Pollinator Seed Mixes. *Restoration Ecology*, 22(6):782–789, 2014. ISSN 1526-100X. doi: 10.1111/rec.12141. URL https://onlinelibrary.wiley.com/doi/abs/10.1111/rec.12141. _eprint: https://onlinelibrary.wiley.com/doi/pdf/10.1111/rec.12141.

Neal M. Williams. Restoration of Nontarget Species: Bee Communities and Pollination Function in Riparian Forests. *Restoration Ecology*, 19(4):450–459, 2011. ISSN 1526-100X. doi: 10.1111/j.1526-100X.2010.00707.x. URL https://onlinelibrary.wiley.com/doi/abs/10.1111/j.1526-100X.2010.00707.x. _eprint: https://onlinelibrary.wiley.com/doi/pdf/10.1111/j.1526-100X.2010.00707.x.

C. Wissel. A universal law of the characteristic return time near thresholds. *Oecologia*, 65(1):101–107, 1984. ISSN 0029-8549. doi: 10.1007/BF00384470.

Yuval R. Zelnik, Nuria Galiana, Matthieu Barbier, Michel Loreau, Eric Galbraith, and Jean-François Arnoldi. How collectively integrated are ecological communities? *Ecology Letters*, 27(1):e14358, 2024. ISSN 1461-0248. doi: 10.1111/ele.14358. URL https://onlinelibrary.wiley.com/doi/abs/10.1111/ele.14358. _eprint: https://onlinelibrary.wiley.com/doi/pdf/10.1111/ele.14358.
