# Supplementary material for: Reviving collapsed plant–pollinator networks from a single species
Source: PLoS Biol. 2024 Oct 4;22(10):e3002826. doi: 10.1371/journal.pbio.3002826 (PMC11482677; doi:10.1371/journal.pbio.3002826)
Supplement: Attachment — Submitted filename: Comment.docx [file pbio.3002826.s040.docx]

Review of the manuscript “Reviving collapsed ecological networks from a single species” (ID: PBIOLOGY-D-24-00019R1) by Gaurav Baruah and Meike Wittmann.

Summary:

The authors investigate how to restore mutualistic ecological networks that have been disrupted by environmental changes. They apply a dynamical eco-evolutionary model informed by empirical plant-pollinator networks and signal propagation theory to examine how network structure, trait variation, and species-specific perturbation influence network recovery. They show that reverting to the original environmental conditions is insufficient for network recovery, but that manipulating a single generalist species can revive the network even in adverse conditions. They also show that network nestedness and trait variation enhance network revival. They suggest that their findings have implications for the conservation and restoration of mutualistic networks.

Review:

The authors address an important and timely question in ecology, namely the resilience and restoration of mutualistic networks. They use a novel combination of theoretical and empirical methods to explore how to restore collapsed networks from a single species. The manuscript is well-written, clear, and concise. The methods are well-explained and the results are supported by figures and tables. The discussion is insightful and connects the findings to the existing literature. The manuscript makes a significant contribution to the field and has the potential to stimulate further research on network dynamics and restoration. However, I have some comments that I think the authors should address before the manuscript can be accepted for publication.

(1) The authors introduce a new model for the problem of interest, but they do not provide any analytical discussion or justification for their approach. They simply set all the parameters according to Table 1 and run numerical simulations. However, they do not explain how they selected the parameter values, nor how they assessed the quality of the simulations. Therefore, the robustness and validity of their results are questionable. The authors should either derive some theoretical analysis or approximation for their model, or conduct more comprehensive and rigorous numerical simulations to demonstrate its performance and limitations under different conditions and assumptions.

(2) The authors do not perform any sensitivity analysis or robustness checks on their model results. They only vary one parameter at a time (the average mutualistic strength, the forcing strength, or the duration of perturbation) and report the outcomes. However, it is possible that the model behavior and the network revival depend on the interactions and combinations of multiple parameters, as well as on the initial conditions and the randomness of the model. It would be useful to explore how the model results change under different scenarios and parameter values, and to test the effects of parameter uncertainty and variability on the model predictions.

(3) The authors adopt a phenomenological model of mutualistic interactions that assumes a Gaussian interaction kernel and a type-2 functional response. While this model has been widely used in previous studies, it has some limitations and assumptions that may affect the generality and applicability of the results. For instance, the model does not account for the effects of spatial structure, dispersal, density-dependence, or frequency-dependence on network dynamics and stability. Furthermore, the model assumes that the phenotypic traits of the species are continuous and normally distributed, which may not be realistic for some plant-pollinator systems. The authors should discuss these limitations and assumptions in more detail and justify their choice of model. They should also investigate the robustness and sensitivity of their results to different model parameters and assumptions, such as the shape and width of the interaction kernel, the handling time, the heritability, and the initial trait distribution.

(4) The authors use a measure of network nestedness (NODF) that is based on the binary adjacency matrix of the network. However, this measure does not account for the variation in interaction strength or frequency among the species, which may also affect the network dynamics and resilience. The authors should consider using a weighted measure of network nestedness that incorporates the interaction strength or frequency. In addition, they should also report and analyze the values of other relevant network metrics, such as modularity, degree distribution, and centrality.

(5) The authors use a perturbation regime that consists of increasing the density of a single species by a constant factor for a fixed duration of time. However, this perturbation regime may not be very realistic or representative of the types of disturbances that occur in natural or human-modified systems. For example, the perturbation may be stochastic, spatially heterogeneous, or affect multiple species simultaneously. The authors should explore the effects of different types of perturbations on network recovery, such as random, targeted, or cascading perturbations, and compare them with their baseline perturbation regime. They should also discuss the ecological and management implications of their perturbation regime and how it relates to real-world scenarios of network restoration.

(6) The authors do not provide a clear definition or operationalization of the concept of network revival which is the first keyword. They seem to use different criteria and thresholds to measure the recovery of the network, such as the proportion of species with density greater than 0.5, the mean pollinator density, the mean plant density, and the mean trait values. However, they do not justify why they chose these criteria and thresholds, and how they relate to the original functionality and diversity of the network. The authors should provide a clear and consistent definition of network revival and explain how they measured it.

(7) The introduction is well-written, but it could be improved by providing more background and context on the problem of network collapse and restoration. The authors should cite more relevant studies on the causes, consequences, and indicators of network collapse, as well as the challenges and strategies of network restoration. They should also state more clearly the main objectives and hypotheses of their study.

(8) The discussion section is insightful, but it could be more balanced and critical. The authors should acknowledge the limitations and uncertainties of their study, and discuss the potential sources of error or bias in their model and analysis. They should also compare and contrast their results with those of other studies, and suggest directions for future research.
